# α-Ketoglutaric acid in Ugi reactions and Ugi/aza-Wittig tandem reactions

**DOI:** 10.3762/bjoc.21.157

**Published:** 2025-10-07

**Authors:** Vladyslav O Honcharov, Yana I Sakhno, Olena H Shvets, Vyacheslav E Saraev, Svitlana V Shishkina, Tetyana V Shcherbakova, Valentyn A Chebanov

**Affiliations:** 1 Institute of Functional Materials Chemistry, State Scientific Institution “Institute for Single Crystals” of National Academy of Sciences of Ukraine, Nauky Ave., 60, 61072, Kharkiv, Ukraine; 2 Faculty of Chemistry, V. N. Karazin Kharkiv National University, Svobody sq., 4, 61022, Kharkiv, Ukraine; 3 Institute of Organic Chemistry, National Academy of Sciences of Ukraine, Akademika Kukharya Street, 5, 02660, Kyiv, Ukraine

**Keywords:** α-ketoglutaric acid, aza-Wittig reaction, multicomponent reaction, quinoxalinone derivative, Ugi reaction

## Abstract

A small library of bis- and tetraamides was synthesized by the Ugi reaction with α-ketoglutaric acid, *tert*-butyl isocyanide, aromatic aldehydes, and aromatic amines. When *o*-azidoanilines were used, azidated peptidomimetics were obtained, the post-cyclization of which by the aza-Wittig reaction yielded a series of substituted 3-(3-oxo-3,4-dihydroquinoxalin-2-yl)propanoic acids containing a pharmacophore quinoxalinone moiety. The tandem Ugi/aza-Wittig combination was also carried out in a one-pot procedure without isolation of the intermediate.

## Introduction

Multicomponent reactions are powerful tools in organic chemistry that enable the synthesis of structurally complex and multifunctional compounds from three or more starting materials in a single synthetic step. They are widely used in drug discovery because, unlike conventional linear synthesis strategies, they enable the preparation of libraries of organic compounds in higher yields and with significantly less time, resources, and chemical waste [1–5].

The Ugi reaction, discovered in 1959 by Ivar Karl Ugi [6], is one of the classic multicomponent reactions, widely used as a green alternative for the synthesis of active pharmaceutical ingredients [7] and opens up the possibility of creating new compounds from the class of peptidomimetics [8–10], which often exhibit diverse biological effects [11] including antidiabetic [12], antiviral [13], antibacterial [8,14–15], and anticancer [16–17] activities.

The use of the Ugi reaction followed by post-cyclization is an effective strategy that yields diverse heterocycle-containing peptidomimetics and requires a minimal number of steps [18]. For example, Mazur et al. [19] developed an efficient method for the preparation of benzodiazepinone derivatives, which showed promising psychotropic effects [20–24], using a tandem combination of Ugi/azide–alkyne cycloaddition reactions. From this point of view, azido amines are promising reagents for use in the Ugi reaction, opening up the possibility of synthesizing various heterocyclic systems such as quinazolines [25], diazepinones [25–26], quinoxalinones [27], diazocinones [28], and imidazolines [29] due to the exceptional reactivity of the azido group. However, the use of azido amines in the Ugi reaction together with oxoacids, which have an additional reaction center for post-cyclizations, is scarcely reported in the literature [25,30–31].

Among the numerous tandem synthetic approaches, the combination of Ugi/aza-Wittig reactions is of particular interest. In recent years, several works have been dedicated to the synthesis of nitrogen-containing heterocyclic compounds using various modifications of this sequence [32–36]. Among others, Yan et al. [31] described a facile way to quinoxalinone derivatives using an Ugi/Staudinger/aza-Wittig tandem combination with pyruvic acid or phenylglyoxylic acid.

**Figure 1:**
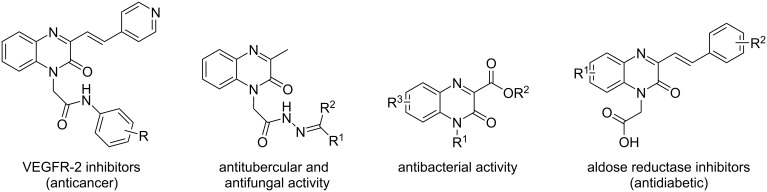
Some biologically active quinoxalinone derivatives.

Quinoxalinones are regarded as privileged heterocyclic moieties in the development of new pharmacologically active organic compounds (Figure 1) [37]. These structures are of great interest to chemists because of their broad spectrum of potential biological effects, including anticancer [38], antibacterial [39] and anti-HIV [40] activities. Furthermore, a quinoxaline-containing commercial drug, caroverine, has been proven effective in the treatment of tinnitus [41]. In addition, in vivo studies in mice and rats with various quinoxalinone derivatives have shown favorable analgesic and anti-inflammatory properties as well as low toxicity of these substances [42]. Biological activity screening also suggests that quinoxalinone-based compounds can inhibit aldose reductase [43], α-amylase and α-glucosidase [44], as well as key enzymes involved in the processes of saturated fatty acid conversion [45] and glycogenolysis [46]. Therefore, these molecules can be considered as potential antidiabetic agents.

**Scheme 1:**
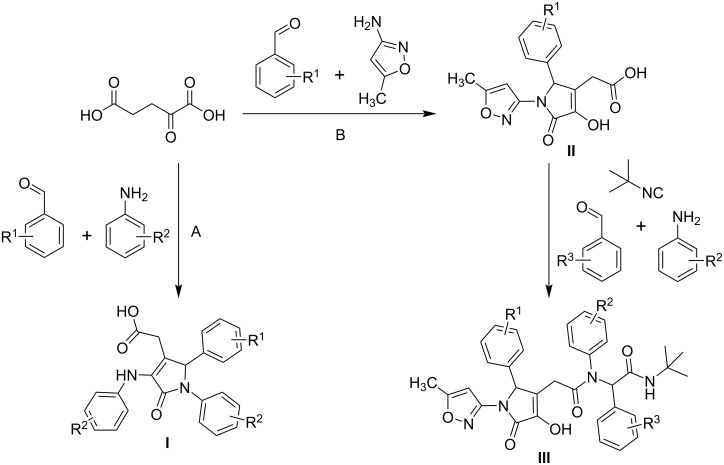
Known multicomponent reactions of KGA.

α-Ketoglutaric acid (KGA) is an attractive precursor for medically oriented syntheses using multicomponent reactions due to its chemical structure as a dibasic keto acid and its role as an important component of numerous biochemical processes [47]. However, there are only a few studies in the literature on multicomponent reactions based on KGA. In 1992, Gein et al. [48] reported a multicomponent reaction involving KGA, aromatic aldehydes, and aromatic amines. This led to the formation of pyrrolone derivatives **I**, which showed anti-inflammatory activity [49–50] (Scheme 1, pathway A). In an earlier study by our research group, Sakhno et al. [51] described a three-component Doebner-type reaction involving KGA, aromatic aldehydes, and 3-amino-5-methylisoxazole, which led to the formation of [2-aryl-4-hydroxy-1-(5-methylisoxazol-3-yl)-5-oxo-2,5-dihydro-1*H*-pyrrol-3-yl]acetic acids **II** (Scheme 1, pathway B). Subsequently, a four-component Ugi reaction of compounds **II** with temperature-controlled formation of diastereomers of peptidomimetics **III** was also studied [52].

Taking into account all mentioned above, this work is dedicated to the synthesis of novel peptidomimetics using the four-component Ugi reaction and the study of a tandem Ugi/aza-Wittig combination based on α-ketoglutaric acid for the preparation of earlier unavailable quinoxalinone derivatives.

## Results and Discussion

**Scheme 2:**
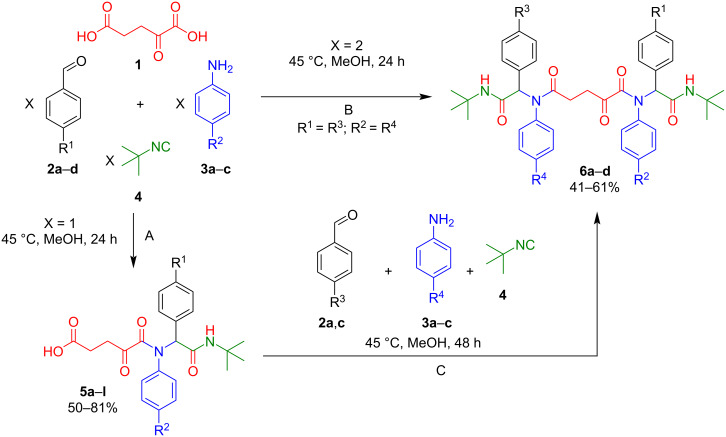
Ugi reaction involving KGA.

We began by studying the behavior of α-ketoglutaric acid in the four-component Ugi reaction with equimolar amounts of the reagents. It was found that stirring of aromatic aldehydes **2a**–**d**, aromatic amines **3a**–**d**, KGA (**1**) and *ter*t-butyl isocyanide (**4**) (in a 1:1:1:1 molar ratio) in methanol for 24 hours at 45 °C resulted in the formation of 5-((aryl)(1-aryl-2-(*tert*-butylamino)-2-oxoethyl)amino)-4,5-dioxopentanoic acids **5a**–**l** in 50–81% yields (Scheme 2, pathway A; Table 1). The compounds **5a**–**l** were isolated easily by adding water to the reaction mass until it became cloudy, whereupon a precipitate of the product was formed and isolated (all experimental procedures can be found in Supporting Information File 1).

**Table 1:** Yields of compounds **5a**–**l**.

Aldehyde	R^1^	Amine	R^2^	Compound	Yield, %

**2a**	4-Cl	**3a**	4-Cl	**5a**	81
**2a**	4-Cl	**3b**	4-CH_3_	**5b**	64
**2a**	4-Cl	**3c**	4-OCH_3_	**5c**	68
**2b**	4-OCH_3_	**3a**	4-Cl	**5d**	50
**2b**	4-OCH_3_	**3b**	4-CH_3_	**5e**	54
**2b**	4-OCH_3_	**3c**	4-OCH_3_	**5f**	52
**2c**	4-COOCH_3_	**3a**	4-Cl	**5g**	57
**2c**	4-COOCH_3_	**3b**	4-CH_3_	**5h**	72
**2c**	4-COOCH_3_	**3c**	4-OCH_3_	**5i**	54
**2d**	4-Br	**3a**	4-Cl	**5j**	77
**2d**	4-Br	**3b**	4-CH_3_	**5k**	73
**2d**	4-Br	**3c**	4-OCH_3_	**5l**	61

Since α-ketoglutaric acid is dibasic, increasing the stoichiometric amounts of the starting materials enables the Ugi reaction involving two carboxyl groups. Thus, mixing KGA (**1**), 4-chlorobenzaldehyde (**2a**), 4-chloroaniline (**3a**), and *tert*-butyl isocyanide (**4**) in a molar ratio of 1:2:2:2 in MeOH and subsequent stirring at a temperature of 45 °C for 24 hours led to the formation of *N*^1^*,N*^5^-bis(2-(*tert*-butylamino)-1-(4-chlorophenyl)-2-oxoethyl)-*N*^1^*,N*^5^-bis(4-chlorophenyl)-2-oxopentanediamide (**6a**) in 55% yield (Scheme 2, pathway B; Table 2).

**Table 2:** Yields of compounds **6a**–**d**.

Acid	Aldehyde	R^3^	Amine	R^4^	Compound	Yield, %

**5a**	**2a**	4-Cl	**3a**	4-Cl	**6a**	45 (55)^a^
	**2a**	4-Cl	**3c**	4-OCH_3_	**6b**	41
	**2a**	4-Cl	**3b**	4-CH_3_	**6c**	61
	**2c**	4-COOCH_3_	**3b**	4-CH_3_	**6d**	42

^a^The yield for the Ugi reaction on two carboxyl groups of KGA is given in parentheses.

Compounds **5** contain a free carboxyl group and thus can also be used as an acidic component in the Ugi reaction. Indeed, it was established that the reaction of 5-((2-(*tert*-butylamino)-1-(4-chlorophenyl)-2-oxoethyl)(4-chlorophenyl)amino)-4,5-dioxopentanoic acid (**5a**) with aldehydes **2a**,**c**, amines **3a**–**c**, and *tert*-butyl isocyanide (**4**) in a molar ratio of 1:1:1 at 45 °C for 48 hours gave *N*^1^,*N*^5^-diaryl-*N*^1^,*N*^5^-bis(1-aryl-2-(*tert*-butylamino)-2-oxoethyl)-2-oxopentanediamides **6a**–**d** in 41–61% yields (Scheme 2, pathway C; Table 2). It is important to note that compounds **6a**–**d** rapidly precipitated from the solution and did not require further purification after filtration.

**Scheme 3:**
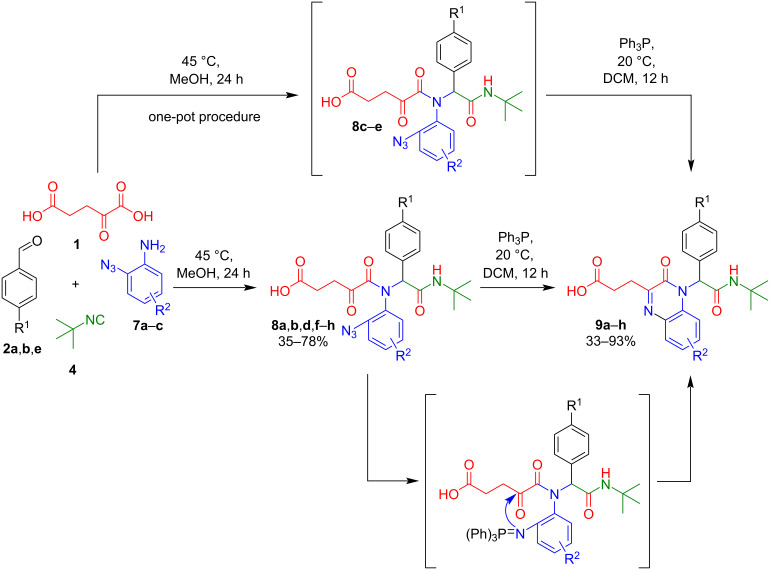
Tandem Ugi/aza-Wittig combination involving KGA.

The use of starting components containing highly reactive groups, for example, an azide group, in the Ugi reaction provides opportunities for various post-cyclizations to obtain nitrogen-containing heterocyclic systems with potential biological activity [18]. To prepare new quinoxalinone derivatives, we studied a tandem combination of Ugi/aza-Wittig reactions involving α-ketoglutaric acid and *o*-azidoanilines. First, 5-((2-arylazido)(1-aryl-2-(*tert*-butylamino)-2-oxoethyl)amino)-4,5-dioxopentanoic acids **8a**,**b**,**d**,**f**–**h** were synthesized by the reaction of KGA (**1**), aldehydes **2**, *o*-azidoanilines **7**, and *tert*-butyl isocyanide (**4)** for 24 hours at 45 °C in 35–78% yields (Scheme 3; Table 3).

**Table 3:** Yields of compounds **8a**,**b**,**d**,**f**–**h** and **9a**–**h**.

Aldehyde	R^1^	Azidoaniline	R^2^	Compound	Yield, %

**2a**	4-Cl	**7a**	4-Cl	**8a**	57
**2a**	4-Cl	**7b**	H	**8b**	54
**2b**	4-OCH_3_	**7b**	H	**8d**	35
**2e**	H	**7a**	4-Cl	**8f**	78
**2e**	H	**7b**	H	**8g**	71
**2e**	H	**7c**	4,6-CH_3_	**8h**	64
**2a**	4-Cl	**7a**	4-Cl	**9a**	82
**2a**	4-Cl	**7b**	H	**9b**	93
**2a**	4-Cl	**7c**	4,6-CH_3_	**9c**	46^a^
**2b**	4-OCH_3_	**7b**	H	**9d**	75 (51)^a^
**2b**	4-OCH_3_	**7c**	4,6-CH_3_	**9e**	35^a^
**2e**	H	**7a**	4-Cl	**9f**	83
**2e**	H	**7b**	H	**9g**	59
**2e**	H	**7c**	4,6-CH_3_	**9h**	47

^a^The yield for the one-pot Ugi/aza-Wittig combination in terms of KGA.

To isolate compounds **8a**,**b**,**d**,**f**–**h**, the reaction mixture was poured onto ice and the resulting precipitate was filtered. These compounds also often had to be additionally purified by column chromatography.

Subsequently, the compounds **8a**,**b**,**d**,**f**–**h** were dissolved in DCM and stirred in the presence of a stoichiometric amount of triphenylphosphine at 20 °C for 12 hours, resulting in the formation of 3-(4-(1-aryl-2-(*tert*-butylamino)-2-oxoethyl)-3-oxo-3,4-dihydroquinoxalin-2-yl)propanoic acids **9a**,**b**,**d**,**f**–**h** in 33–93% yields (Scheme 3; Table 3). According to the literature [31,53], this reaction proceeds through the formation of an iminophosphorane intermediate (Scheme 3), the product of a Staudinger reaction, which, however, was not isolated because it easily undergoes intramolecular cyclization on a sufficiently electrophilic carbonyl carbon atom with the elimination of triphenylphosphine oxide via the aza-Wittig reaction.

It should be noted that the best method for the isolation of quinoxalinones **9** was column chromatography using an elution gradient of hexane/ethyl acetate 3:1 to hexane/ethyl acetate 1:2 with the addition of 0.1% formic acid. The use of other eluents such as acetonitrile and different ratios of hexane/ethyl acetate, dichloromethane/methanol as well as the application of methods for the isolation of triphenylphosphine oxide by complexation with calcium and magnesium salts [54–55] or precipitation from non-polar solvents such as hexane and toluene did not lead to a complete separation of quinoxalinones **9** and Ph_3_PO. We attribute this to the ability of Ph_3_PO to form a strong hydrogen bond between the phosphoryl oxygen and a proton from the donor group of the second molecule [56–58], in this case a carboxyl proton.

In order to avoid the step of isolation of the intermediate azide derivatives **8**, we also studied a one-pot method for the synthesis of compounds **9**. For this KGA **1**, aldehydes **2a**,**b**, azidoanilines **7b**,**c**, and *tert*-butyl isocyanide (**4**) were stirred in methanol at 45 °C for 24 hours. The solvent was then evaporated, the residue dissolved in DCM, and stirred in the presence of Ph_3_P for 12 hours at 20 °C to give compounds **9c**–**e** in 35–51% yields (Table 3).

Identification and structure determination of the compounds obtained was based on elemental analysis, mass spectrometry, ^1^H and ^13^C NMR spectroscopy, and by X-ray diffraction study (see Experimental part). The ^1^H NMR spectra of compounds **5** are characterized by the following signals: a broad carboxyl group proton singlet at 12.10–12.17 ppm, an NH group proton singlet at 7.74–8.02 ppm, an aromatic protons multiplet at 6.52–8.15 ppm, a CH group singlet at 5.93–6.12 ppm, triplets for two CH_2_ groups in the range of 2.04–2.89 ppm, a *tert*-butyl group singlet at 1.23–1.25 ppm, and signals for protons of the functional group. It should be noted that a doubling of the ^1^H NMR signals is observed for the azide derivatives **8** compared to compounds **5**, which can be clearly seen in the spectrum of compound **8h** with both substituted *ortho*-positions of the amine moiety. In our opinion, this indicates the presence of rotamerism [59–60] caused by the restricted rotation of the sterically hindered amide group.

The ^1^H NMR spectra of compounds **6** are marked by the following differences: the absence of the proton signal for the carboxyl group, the presence of two singlets for NH groups in the range of 7.61–7.96 ppm and two singlets for CH groups at 5.96–6.05 ppm, increased integral intensity of the aromatic multiplet in the range of 6.28–7.81 ppm, corresponding to 16 protons, multiplets for CH_2_ groups at 1.78–2.96 ppm, and two signals for *tert*-butyl groups protons at 1.21–1.24 ppm. The increase in signal multiplicity compared to compounds **5** could indicate the presence of diastereomerism and restricted amide rotation in compounds **6**.

**Figure 2:**
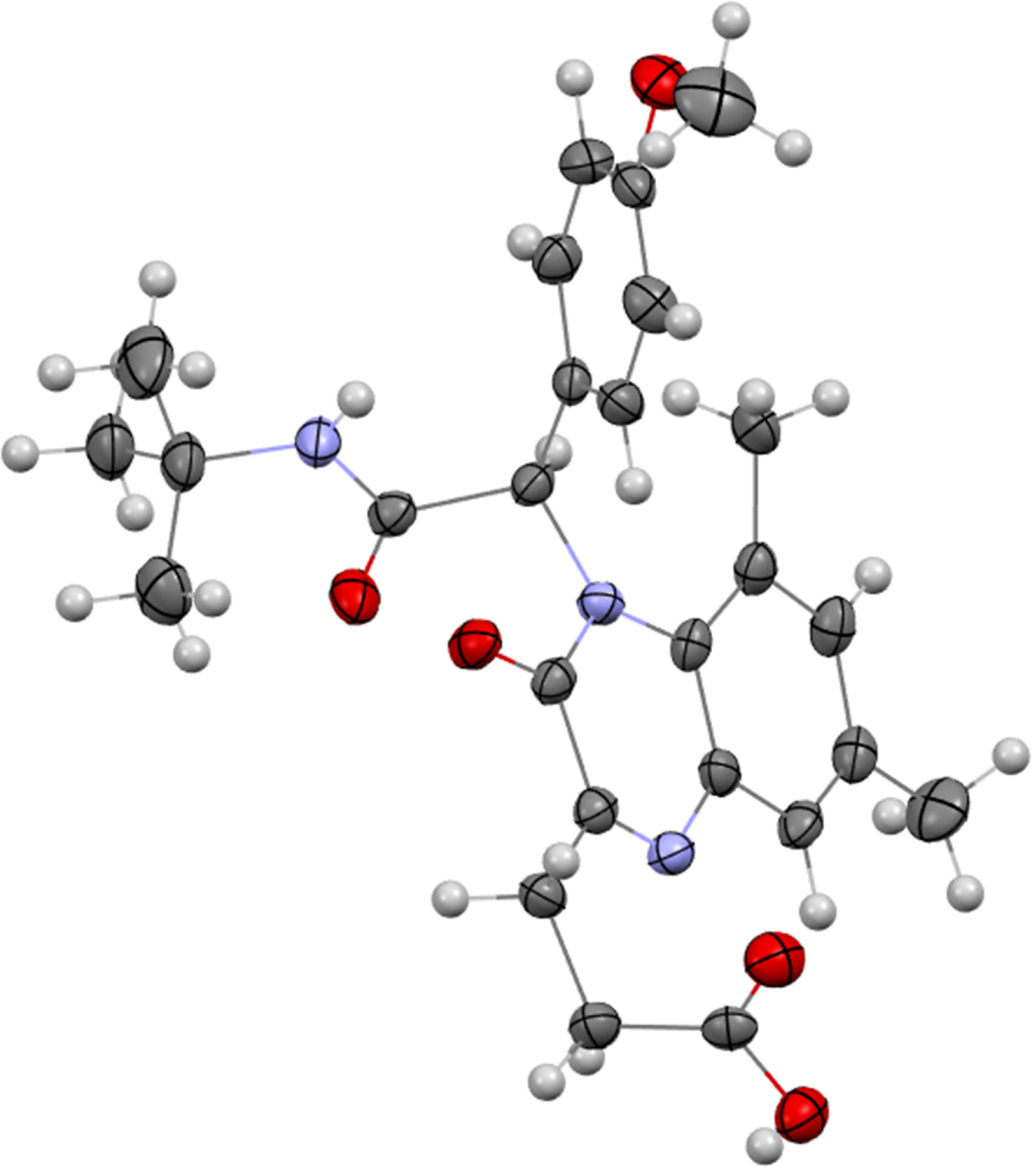
Molecular structure of 3-(4-(2-(*tert*-butylamino)-1-(4-methoxyphenyl)-2-oxoethyl)-5,7-dimethyl-3-oxo-3,4-dihydroquinoxalin-2-yl)propanoic acid (**9e**) according to X-ray diffraction data.

In the ^1^H NMR spectra of quinoxalinones **9**, a shift of the methylene groups signal to a weaker field (2.90–3.14 ppm and 2.63–2.77 ppm) is observed in comparison to compounds **8** (2.60–3.06 ppm and 2.14–2.35 ppm). Furthermore, comparison of the ^13^C NMR spectra of compounds **8** and **9** shows a characteristic shift of the signal in the 198 ppm region, corresponding to the carbonyl carbon of compounds **8**, to a stronger field in quinoxalinones **9**, where cyclization of the keto group has occurred. The structure of quinoxalinones of type **9** was finally assigned based on X-ray diffraction analysis made for 3-(4-(2-(*tert*-butylamino)-1-(4-methoxyphenyl)-2-oxoethyl)-5,7-dimethyl-3-oxo-3,4-dihydroquinoxalin-2-yl)propanoic acid (**9e**) (Figure 2).

## Conclusion

To summarize, we have studied the Ugi reaction and its tandem combination with aza-Wittig reactions involving α-ketoglutaric acid. Depending on the stoichiometric ratio of the reagents, the Ugi reaction involves one or both carboxyl groups of KGA, leading to 5-((aryl)(1-aryl-2-(*tert*-butylamino)-2-oxoethyl)amino)-4,5-dioxopentanoic acids or *N*^1^,*N*^5^-diaryl-*N*^1^,*N*^5^-bis(1-aryl-2-(*tert*-butylamino)-2-oxoethyl)-2-oxopentanediamides, respectively. The tandem combination of Ugi and aza-Wittig reactions involving α-ketoglutaric acid and *o*-azidonanilines is an efficient method for the preparation of earlier unavailable quinoxalinone derivatives and can be carried out as a one-pot procedure without isolation of an intermediate azide-containing Ugi reaction product. Both quinoxalinones and Ugi reaction products may be valuable for organic synthesis and medicinal chemistry because the presence of a free carboxyl group makes them promising for use as building blocks for the construction of complex heterocyclic structures, including those using multicomponent reactions.

## Supporting Information

File 1 General synthetic procedures, characterization of compounds, ^1^H and ^13^C NMR spectra and X-ray data.

## Data Availability

Data generated and analyzed during this study is available from the corresponding author upon reasonable request.
