# Supplementary material for: α-Ketoglutaric acid in Ugi reactions and Ugi/aza-Wittig tandem reactions
Source: Beilstein J Org Chem. 2025 Oct 7;21:2021–9. doi: 10.3762/bjoc.21.157 (PMC12522154; doi:10.3762/bjoc.21.157)
Supplement: File 1 — General synthetic procedures, characterization of compounds, 1H and 13C NMR spectra and X-ray data. [file Beilstein_J_Org_Chem-21-2021-s001.pdf]

BEILSTEIN JOURNAL OF ORGANIC CHEMISTRY

Supporting Information

for

α-Ketoglutaric acid in Ugi reactions and Ugi/aza-Wittig tandem reactions

Vladyslav O. Honcharov, Yana I. Sakhno, Olena H. Shvets, Vyacheslav E. Saraev, Svitlana V. Shishkina, Tetyana V. Shcherbakova and Valentyn A. Chebanov

Beilstein J. Org. Chem. **2025,** *21,* 2021–2029. doi:10.3762/bjoc.21.157

General synthetic procedures, characterization of compounds, ^{1}H and ^{13}C NMR spectra and X-ray data

License and Terms: This is a supporting information file under the terms of the Creative Commons Attribution License (https://creativecommons.org/licenses/by/4.0). Please note that the reuse, redistribution and reproduction in particular requires that the author(s) and source are credited and that individual graphics may be subject to special legal provisions.
The license is subject to the *Beilstein Journal of Organic Chemistry* terms and conditions: (https://www.beilstein-journals.org/bjoc/terms)

General

^{1}H and ^{13}C NMR spectra were recorded with Varian MR-400, Bruker Avance III, Bruker Avance DRX, and Varian Unity INOVA spectrometers (400 and 100 MHz, respectively) in DMSO-d_6. Mass spectra were recorded with a Shimadzu LCMS-2020 spectrometer. Elemental analysis was performed using an EuroVector EA-3000 automatic elemental analyzer. The melting points were measured with a Stuart SMP10 electronic melting point apparatus and are uncorrected. The progress of the reactions and the purity of the compounds obtained were monitored by thin-layer chromatography on ALUGRAM Xtra SIL G UV254 plates developed with mixtures of EtOAc and hexane. The starting *o*-azidoanilines were obtained according to the methods described in the literature [1,2]. Aromatic aldehydes, aromatic amines, α-ketoglutaric acid, and *tert*-butyl isocyanide were commercially available.

X-ray experimental part

The colorless crystals of compound **9e** ($C_{26}H_{31}N_3O_5$, C_2H_6O) are triclinic. At 173 K a = 9.1980(13), b = 9.5476(13), c = 15.400(3) Å, α = 92.334(9)°, β = 94.801(10)°, γ = 90.029(9)°, V = 1346.5(3) Å^3, M_r = 511.60, Z = 2, space group $P\bar{1}$, d_{calc}= 1.262 g/cm^3, μ(Mo K) = 0.089 mm^{-1}, F(000) = 548. Intensities of 15385 reflections (14137 independent, R_{int} = 0.0859) were measured on a Bruker APEX II diffractometer (graphite monochromated Mo Kα radiation, CCD detector, φ- and ω-scanning, $2\Theta_{max}$ = 50°). The structure was solved by direct method using OLEX2 [3] package with SHELXT [4] and SHELXL modules [5]. Positions of the hydrogen atoms were located from electron density difference maps and refined using "riding" model with U_{iso} = nU_{eq} (n = 1.5 for methyl group and n = 1.2 for other hydrogen atoms) of the carrier atom. Full-matrix least-squares refinement against F^2 in anisotropic approximation for non-

hydrogen atoms using 14137 reflections was converged to $wR_2 = 0.2360$ ($R_1 = 0.0774$ for 7589 reflections with $F > 4\sigma(F)$, $S = 1.005$). The final atomic coordinates, and crystallographic data for molecule **9e** have been deposited to with the Cambridge Crystallographic Data Centre, 12 Union Road, CB2 1EZ, UK (fax: +44-1223-336033; e-mail: deposit@ccdc.cam.ac.uk) and are available on request quoting the deposition numbers CCDC 2464193).

Synthetic procedures

Synthesis of compounds 5

General procedure for the synthesis of compounds 5a–l. Amines **3a**–**c** (0.68 mmol) and aldehydes **2a**–**d** (0.68 mmol) were dissolved in 2 mL of methanol in a round-bottomed flask and stirred at 25 °C for 30 minutes. Then, α-ketoglutaric acid (**1**, 0.68 mmol, 100 mg) was added to the reaction mixture and stirred for 30 minutes. Then, *tert*-butyl isocyanide (**4**, 0.68 mmol, 77 µL) was added and the reaction mixture was stirred at 45 °C for 24 hours. The completeness of the reaction was determined by thin-layer chromatography (hexane/ethyl acetate 1:2). The reaction mixture was cooled, and a small amount of water was added until it became cloudy. The precipitate of compounds **5a**–**l** formed after the addition of water was filtered and dried in vacuo.

5-((2-(*tert*-Butylamino)-1-(4-chlorophenyl)-2-oxoethyl)(4-chlorophenyl)amino)-4,5-dioxopentanoic acid (5a). Yield 81% (265 mg), white solid, m.p. = 169-171 °C, Rf = 0.57.

^{1}H NMR spectrum (400 MHz, DMSO-d_6), δ, ppm: 12.17 (s, 1H, COOH), 7.97 (s, 1H, NH), 6.98-7.35 (m, 8H, ArH), 6.04 (s, 1H, CH), 2.88 (t, J = 6.5 Hz, 2H, CH_2), 2.26 (t, J = 6.6 Hz, 2H, CH_2), 1.24 (s, 9H, *t*-Bu).

^{13}C NMR spectrum (100 MHz, DMSO-d_6), δ, ppm: 198.8, 172.9, 167.6, 166.3, 136.3, 133.3, 132.7, 132.6, 132.2, 131.9, 128.3, 128.1, 62.8, 50.6, 35.0, 28.3, 26.6.

Mass spectrum (ESI), m/z: 477 (100) $[M-H]^-$.

Elemental analysis calcd for $C_{23}H_{24}Cl_2N_2O_5$: C 57.63, H 5.05, N 5.84; found: C 57.51, H 5.14, N 5.93.

5-((2-(*tert*-Butylamino)-1-(4-chlorophenyl)-2-oxoethyl)(*p*-tolyl)amino)-4,5-dioxopentanoic acid (5b) Yield 64% (200 mg), white solid, m.p. = 150-152 °C, Rf = 0.65.

^1H NMR spectrum (400 MHz, DMSO-d_6), δ, ppm: 7.89 (s, 1H, NH), 6.84-7.33 (m, 8H, ArH), 6.00 (s, 1H, CH), 2.76 (t, J = 6.8 Hz, 2H, CH_2), 2.19 (t, J = 6.4 Hz, 2H, CH_2), 2.16 (s, 3H, CH_3), 1.24 (s, 9H, *t*-Bu).

^{13}C NMR spectrum (100 MHz, DMSO-d_6), δ, ppm: 199.1, 172.8, 167.6, 166.9, 137.5, 134.4, 133.6, 132.5, 131.8, 130.3, 128.9, 128.0, 62.7, 50.5, 35.1, 28.3, 26.7, 20.6.

Mass spectrum (ESI), m/z: 457 (100) $[M-H]^-$.

Elemental analysis calcd for $C_{24}H_{27}ClN_2O_5$: C 62.81, H 5.93, N 6.10; found: C 62.71, H 6.02, N 6.17.

5-((2-(*tert*-Butylamino)-1-(4-chlorophenyl)-2-oxoethyl)(4-methoxyphenyl)amino)-4,5-dioxopentanoic acid (5c). Yield 68% (220 mg), white solid, m.p. = 155-157 ˚C, Rf = 0.36.

^1H NMR spectrum (400 MHz, DMSO-d_6), δ, ppm: 12.15 (s, 1H, COOH), 7.89 (s, 1H, NH), 6.61-7.33 (m, 8H, ArH), 5.98 (s, 1H, CH), 3.64 (s, 3H, OCH_3), 2.75 (t, J = 7.1 Hz, 2H, CH_2), 2.20 (t, J = 6.6 Hz, 2H, CH_2), 1.24 (s, 9H, *t*-Bu).

^{13}C NMR spectrum (100 MHz, DMSO-d_6), δ, ppm: 199.2, 172.8, 167.8, 167.0, 158.6, 133.6, 132.5, 132.0, 131.9, 129.3, 128.0, 113.4, 62.7, 55.1, 50.5, 35.1, 28.3, 26.7.

Mass spectrum (ESI), m/z: 473 (100) $[M-H]^-$.

Elemental analysis calcd for $C_{24}H_{27}ClN_2O_6$: C 60.70, H 5.73, N 5.90; found: C 60.61, H 5.79, N 5.99.

5-((2-(*tert*-Butylamino)-1-(4-methoxyphenyl)-2-oxoethyl)(4-chlorophenyl)amino)-4,5-dioxopentanoic acid (5d). Yield 50% (160 mg), white solid, m.p. = 144-146 ˚C, Rf = 0.45.

^{1}H NMR spectrum (400 MHz, DMSO-d_6), δ, ppm: 12.14 (s, 1H, COOH), 7.85 (s, 1H, NH), 6.63-7.40 (m, 8H, ArH), 5.99 (s, 1H, CH), 3.65 (s, 3H, OCH_3), 2.85 (t, J = 6.5 Hz, 2H, CH_2), 2.25 (t, J = 6.4 Hz, 2H, CH_2), 1.25 (s, 9H, *t*-Bu).

^{13}C NMR spectrum (100 MHz, DMSO-d_6), δ, ppm: 198.9, 172.9, 168.3, 166.3, 158.8, 136.5, 132.3, 131.4, 128.1, 125.9, 113.5, 63.0, 55.0, 50.5, 35.0, 28.4, 26.6.

Mass spectrum (ESI), m/z: 473 (100) $[M-H]^-$.

Elemental analysis calcd for $C_{24}H_{27}ClN_2O_6$: C 60.70, H 5.73, N 5.90; found: C 60.59, H 5.78, N 5.98.

5-((2-(*tert*-Butylamino)-1-(4-methoxyphenyl)-2-oxoethyl)(*p*-tolyl)amino)-4,5-dioxopentanoic acid (5e). Yield 54% (165 mg), white solid, m.p. = 131-133 ˚C, Rf = 0.38.

^{1}H NMR spectrum (400 MHz, DMSO-d_6), δ, ppm: 12.13 (s, 1H, COOH), 7.78 (s, 1H, NH), 6.58-7.30 (m, 8H, ArH), 5.96 (s, 1H, CH), 3.64 (s, 3H, OCH_3), 2.74 (t, J = 5.9 Hz, 2H, CH_2), 2.04-2.28 (m, 2H, CH_2), 2.15 (s, 3H, CH_3), 1.25 (s, 9H, *t*-Bu).

^{13}C NMR spectrum (100 MHz, DMSO-d_6), δ, ppm: 199.2, 172.8, 168.4, 166.9, 158.6, 137.2, 134.6, 131.3, 130.4, 128.7, 126.2, 113.3, 62.8, 55.0, 50.4, 35.1, 28.4, 26.7, 20.6.

Mass spectrum (ESI), m/z: 453 (100) $[M-H]^-$.

Elemental analysis calcd for $C_{25}H_{30}N_2O_6$: C 66.06, H 6.65, N 6.16; found: C 65.92, H 6.71, N 6.28.

5-((2-(*tert*-Butylamino)-1-(4-methoxyphenyl)-2-oxoethyl)(4-methoxyphenyl)amino)-4,5-dioxopentanoic acid (5f). Yield 52% (165 mg), white solid, m.p. = 135-137 ˚C, Rf = 0.40.

^{1}H NMR spectrum (400 MHz, DMSO-d_6), δ, ppm: 12.10 (s, 1H, COOH), 7.74 (s, 1H, NH), 6.58-7.25 (m, 8H, ArH), 5.93 (s, 1H, CH), 3.65 (s, 3H, OCH_3), 3.64 (s, 3H, OCH_3), 2.71 (t, J = 6.0 Hz, 2H, CH_2), 2.18 (t, J = 6.6 Hz, 2H, CH_2), 1.25 (s, 9H, *t*-Bu).

^{13}C NMR spectrum (100 MHz, DMSO-d_6), δ, ppm: 199.4, 172.8, 168.5, 167.1, 158.7, 158.4, 132.1, 131.3, 129.5, 126.3, 113.4, 113.2, 62.8, 55.1, 55.0, 50.4, 35.1, 28.4, 26.7.

Mass spectrum (ESI), m/z: 469 (100) $[M-H]^-$.

Elemental analysis calcd for $C_{25}H_{30}N_2O_7$: C 63.82, H 6.43, N 5.95; found: C 63.75, H 6.49, N 6.07.

5-((2-(*tert*-Butylamino)-1-(4-(methoxycarbonyl)phenyl)-2-oxoethyl)(4-chlorophenyl)amino)-4,5-dioxopentanoic acid (5g). Yield 57% (195 mg), white solid, m.p. = 156-158 ˚C, Rf = 0.50.

^{1}H NMR spectrum (400 MHz, DMSO-d_6), δ, ppm: 12.15 (s, 1H, COOH), 8.02 (s, 1H, NH), 7.04-8.15 (m, 8H, ArH), 6.12 (s, 1H, CH), 3.79 (s, 3H, $COOCH_3$), 2.89 (t, J = 6.6 Hz, 2H, CH_2), 2.26 (t, J = 6.3 Hz, 2H, CH_2), 1.25 (s, 9H, *t*-Bu).

^{13}C NMR spectrum (100 MHz, DMSO-d_6), δ, ppm: 198.8, 172.9, 167.3, 166.3, 165.8, 139.7, 136.3, 132.1, 130.4, 129.0, 128.8, 128.3, 63.1, 52.2, 50.7, 35.0, 28.3, 26.6.

Mass spectrum (ESI), m/z: 501 (100) $[M-H]^-$.

Elemental analysis calcd for $C_{25}H_{27}ClN_2O_7$: C 59.70, H 5.41, N 5.57; found: C 59.61, H 5.48, N 5.67.

5-((2-(*tert*-Butylamino)-1-(4-(methoxycarbonyl)phenyl)-2-oxoethyl)(*p*-tolyl)amino)-4,5-dioxopentanoic acid (5h). Yield 72% (235 mg), white solid, m.p. = 168-170 ˚C, Rf = 0.52.

^{1}H NMR spectrum (400 MHz, DMSO-d_6), δ, ppm: 12.15 (s, 1H, COOH), 7.95 (s, 1H, NH), 6.81-7.80 (m, 8H, ArH), 6.09 (s, 1H, CH), 3.78 (s, 3H, $COOCH_3$), 2.79 (t, J = 7.0 Hz, 2H, CH_2), 2.20 (t, J = 6.8 Hz, 2H, CH_2), 2.13 (s, 3H, CH_3), 1.24 (s, 9H, *t*-Bu).

^{13}C NMR spectrum (100 MHz, DMSO-d_6), δ, ppm: 199.0, 172.8, 167.4, 166.9, 165.8, 140.0, 137.5, 134.4, 130.4, 130.2, 128.8, 128.7, 63.1, 52.1, 50.6, 35.1, 28.3, 26.7, 20.5.

Mass spectrum (ESI), m/z: 481 (100) $[M-H]^-$.

Elemental analysis calcd for $C_{26}H_{30}N_2O_7$: C 64.72, H 6.27, N 5.81; found: C 64.65, H 6.20, N 5.89.

5-((2-(*tert*-Butylamino)-1-(4-(methoxycarbonyl)phenyl)-2-oxoethyl)(4-methoxyphenyl)amino)-4,5-dioxopentanoic acid (5i). Yield 54% (185 mg), white solid, m.p. = 132-134 ˚C, Rf = 0.54.

^{1}H NMR spectrum (400 MHz, DMSO-d_6), δ, ppm: 12.17 (s, 1H, COOH), 7.94 (s, 1H, NH), 6.52-7.82 (m, 8H, ArH), 6.06 (s, 1H, CH), 3.78 (s, 3H, $COOCH_3$), 3.61 (s, 3H, OCH_3), 2.76 (t, J = 6.8 Hz, 2H, CH_2), 2.20 (t, J = 6.5 Hz, 2H, CH_2), 1.24 (s, 9H, *t*-Bu).

^{13}C NMR spectrum (100 MHz, DMSO-d_6), δ, ppm: 199.2, 172.8, 167.5, 167.1, 165.9, 158.6, 140.0, 131.9, 130.4, 129.4, 128.9, 128.8, 113.4, 63.1, 55.1, 52.1, 50.6, 35.1, 28.3, 26.7.

Mass spectrum (ESI), m/z: 497 (100) $[M-H]^-$.

Elemental analysis calcd for $C_{26}H_{30}N_2O_8$: C 62.64, H 6.07, N 5.62; found: C 62.49, H 5.99, N 5.78.

5-((1-(4-Bromophenyl)-2-(*tert*-butylamino)-2-oxoethyl)(4-chlorophenyl)amino)-4,5-dioxopentanoic acid (5j). Yield 77% (275 mg), white solid, m.p. = 157-159 ˚C, Rf = 0.40.

^{1}H NMR spectrum (400 MHz, DMSO-d_6), δ, ppm: 12.16 (s, 1H, COOH), 7.97 (s, 1H, NH), 6.93-7.45 (m, 8H, ArH), 6.01 (s, 1H, CH), 2.88 (t, J = 6.5 Hz, 2H, CH_2), 2.25 (t, J = 6.4 Hz, 2H, CH_2), 1.24 (s, 9H, *t*-Bu).

^{13}C NMR spectrum (100 MHz, DMSO-d_6), δ, ppm: 198.8, 172.8, 167.5, 166.3, 136.3, 133.7, 132.5, 132.1, 131.0, 128.3, 121.3, 62.8, 50.6, 35.0, 28.3, 26.6.

Mass spectrum (ESI), m/z: 521 (77) $[M-H]^-$.

Elemental analysis calcd for $C_{23}H_{24}BrClN_2O_5$: C 52.74, H 4.62, N 5.35; found: C 52.62, H 4.71, N 5.43.

5-((1-(4-Bromophenyl)-2-(*tert*-butylamino)-2-oxoethyl)(*p*-tolyl)amino)-4,5-dioxopentanoic acid (5k). Yield 73% (250 mg), white solid, m.p. = 133-135 ˚C, Rf = 0.63.

^{1}H NMR spectrum (400 MHz, DMSO-d_6), δ, ppm: 7.90 (s, 1H, NH), 6.87-7.45 (m, 8H, ArH), 5.98 (s, 1H, CH), 2.76 (t, J = 6.8 Hz, 2H, CH_2), 2.10-2.24 (m, 2H, CH_2), 2.16 (s, 3H, CH_3), 1.23 (s, 9H, *t*-Bu).

^{13}C NMR spectrum (100 MHz, DMSO-d_6), δ, ppm: 199.1, 172.9, 167.6, 166.9, 137.5, 134.4, 134.0, 132.2, 130.9, 130.3, 128.9, 121.2, 62.8, 50.5, 35.2, 28.3, 26.9, 20.6.

Mass spectrum (ESI), m/z: 501 (99) $[M-H]^-$.

Elemental analysis calcd for $C_{24}H_{27}BrN_2O_5$: C 57.26, H 5.41, N 5.57; found: C 57.18, H 5.52, N 5.66.

5-((1-(4-Bromophenyl)-2-(*tert*-butylamino)-2-oxoethyl)(4-methoxyphenyl)amino)-4,5-dioxopentanoic acid (5l). Yield 61% (215 mg), white solid, m.p. = 133-135 ˚C, Rf = 0.55.

^{1}H NMR spectrum (400 MHz, DMSO-d_6), δ, ppm: 12.13 (s, 1H, COOH), 7.88 (s, 1H, NH), 6.61-7.44 (m, 8H, ArH), 5.96 (s, 1H, CH), 3.64 (s, 3H, OCH_3), 2.75 (t, *J* = 6.1 Hz, 2H, CH_2), 2.20 (t, *J* = 6.7 Hz, 2H, CH_2), 1.24 (s, 9H, *t*-Bu).

^{13}C NMR spectrum (100 MHz, DMSO-d_6), δ, ppm: 199.2, 172.8, 167.7, 167.0, 158.6, 134.0, 132.2, 132.0, 130.9, 129.3, 121.2, 113.4, 62.7, 55.1, 50.5, 35.1, 28.3, 26.7

Mass spectrum (ESI), m/z: 517 (98) $[M-H]^{-}$.

Elemental analysis calcd for $C_{24}H_{27}BrN_2O_6$: C 55.50, H 5.24, N 5.39; found: C 55.41, H 5.29, N 5.48.

Synthesis of compounds 6

General procedure for the synthesis of compounds 6a–d from acid 5a. Amines **3a**–**c** (0.2 mmol) and aldehydes **2a**,**c** (0.2 mmol) were dissolved in 2 mL of methanol in a round-bottomed flask and stirred at 25 °C for 30 minutes. Subsequently, 5-((2-(*tert*-butylamino)-1-(4-chlorophenyl)-2-oxoethyl)(4-chlorophenyl)amino)-4,5-dioxopentanoic acid (**5a**, 0.2 mmol, 100 mg) was added to the reaction mixture and stirred for 30 minutes. Then, *tert*-butyl isocyanide (**4**, 0.2 mmol, 24 µL) was added and the reaction mixture was stirred at 45 °C for 48 hours until a precipitate was formed. The resulting precipitate of compounds **6a**–**d** was filtered and dried in vacuo.

Synthesis of compound 6a from α-ketoglutaric acid. 4-Chloroaniline (**3a**, 1.36 mmol, 175 mg) and 4-chlorobenzaldehyde (**2a**, 1.36 mmol, 190 mg) were dissolved in 4 mL of methanol and stirred at 25 °C for 30 min. Subsequently, α-ketoglutaric acid (**1**, 0.68 mmol, 100 g) was added to the reaction mixture and stirred for 30 minutes. Then, *tert*-butyl isocyanide (**4**, 1.36 mmol, 154 µL) was added and the

reaction mixture was stirred at 45 °C for 24 hours until a precipitate was formed. The resulting precipitate of compound **6a** was filtered and dried in vacuo.

***N*[1],*N*[5]-bis(2-(*tert*-Butylamino)-1-(4-chlorophenyl)-2-oxoethyl)-*N*[1],*N*[5]-bis(4-chlorophenyl)-2-oxopentanediamide (6a).** Yield 45% (75 mg) from acid **5a**, 55% (305 mg) from α-ketoglutaric acid, white solid, m.p. = 199-201 ˚C.

^{1}H NMR spectrum (400 MHz, DMSO-d_6), δ, ppm: 7.96 (s, 1H, NH), 7.76 (s, 1H, NH), 6.47-7.53 (m, 16H, ArH), 6.01 (s, 1H, CH), 5.99 (s, 1H, CH), 2.65-2.96 (m, 2H, CH_2), 1.80-2.06 (m, 2H, CH_2), 1.24 (s, 9H, *t*-Bu), 1.22 (s, 9H, *t*-Bu).

^{13}C NMR spectrum (100 MHz, DMSO-d_6), δ, ppm: 199.1, 199.0, 169.9, 168.6, 168.5, 167.5, 166.1, 138.2, 136.2, 134.5, 133.2, 132.7, 132.6, 132.5, 132.3, 132.2, 132.1, 131.8, 131.7, 128.5, 128.2, 128.1, 127.9, 62.7, 50.6, 50.4, 35.3, 28.4, 28.3, 27.8.

Mass spectrum (ESI), m/z: 813 (30) $[M+H]^+$, 811 (74) $[M-H]^-$.

Elemental analysis calcd for $C_{41}H_{42}Cl_4N_4O_5$: C 60.60, H 5.21, N 6.89; found: C 60.52, H 5.28, N 6.95.

***N*[1],*N*[5]-bis(2-(*tert*-Butylamino)-1-(4-chlorophenyl)-2-oxoethyl)-*N*[1]-(4-chlorophenyl)-*N*[5]-(4-methoxyphenyl)-2-oxopentanediamide (6b).** Yield 41% (65 mg), white solid, m.p. = 211-213 ˚C.

^{1}H NMR spectrum (400 MHz, DMSO-d_6), δ, ppm: 7.91 (s, 1H, NH), 7.61 (s, 1H, NH), 6.40-7.30 (m, 16H, ArH), 6.01 (s, 1H, CH), 5.96 (s, 1H, CH), 3.66 (s, 3H, OCH_3), 2.69-2.92 (m, 2H, CH_2), 1.82-2.02 (m, 2H, CH_2), 1.24 (s, 9H, *t*-Bu), 1.22 (s, 9H, *t*-Bu).

^{13}C NMR spectrum (100 MHz, DMSO-d_6), δ, ppm: 199.0, 170.4, 168.6, 167.4, 166.2, 158.2, 136.2, 134.9, 133.2, 132.6, 132.5, 132.1, 132.0 131.8, 131.7, 128.1, 128.0, 127.7, 113.5, 62.8, 62.6, 55.0, 50.5, 50.2, 35.3, 28.3, 28.2, 27.7.

Mass spectrum (ESI), m/z: 807 (38) $[M+H]^+$, 843 (100) $[M+K-2H]^-$.

Elemental analysis calcd for $C_{42}H_{45}Cl_3N_4O_6$: C 62.42, H 5.61, N 6.93; found: C 62.31, H 5.72, N 7.04.

***N*[1],*N*[5]-bis(2-(*tert*-Butylamino)-1-(4-chlorophenyl)-2-oxoethyl)-*N*[1]-(4-chlorophenyl)-2-oxo-*N*[5]-(*p*-tolyl)pentanediamide (6c).** Yield 61% (95 mg), white solid, m.p. = 187-189 ˚C.

^{1}H NMR spectrum (400 MHz, DMSO-d_6), δ, ppm: 7.95 (s, 1H, NH), 7.66 (s, 1H, NH), 6.28-7.34 (m, 16H, ArH), 6.01 (s, 1H, CH), 5.96 (s, 1H, CH), 2.64-2.96 (m, 2H, CH_2), 2.18 (s, 3H, CH_3), 1.79-2.05 (m, 2H, CH_2), 1.24 (s, 9H, *t*-Bu), 1.22 (s, 9H, *t*-Bu).

^{13}C NMR spectrum (100 MHz, DMSO-d_6), δ, ppm: 199.0, 170.2, 168.5, 167.4, 166.2, 136.9, 136.7, 136.2, 134.8, 133.2, 132.6, 132.5, 132.1, 132.0, 131.7, 130.4, 128.9, 128.1, 128.0, 127.7, 62.9, 62.6, 50.5, 50.3, 35.3, 28.3, 28.2, 27.8, 20.5.

Mass spectrum (ESI), m/z: 791 (39) $[M+H]^+$, 789 (39) $[M-H]^-$.

Elemental analysis calcd for $C_{42}H_{45}Cl_3N_4O_5$: C 63.68, H 5.73, N 7.07; found: C 63.76, H 5.78, N 7.18.

Methyl 4-(12,13-bis(4-chlorophenyl)-2,2,16,16-tetramethyl-4,7,10,11,14-pentaoxo-6-(*p*-tolyl)-3,6,12,15-tetraazaheptadecan-5-yl)benzoate (6d). Yield 42% (70 mg), white solid, m.p. = 214-216 ˚C.

^{1}H NMR spectrum (400 MHz, DMSO-d_6), δ, ppm: 7.95 (s, 1H, NH), 7.72 (s, 1H, NH), 6.43-7.81 (m, 16H, ArH), 6.05 (s, 1H, CH), 6.01 (s, 1H, CH), 3.78 (s, 3H, $COOCH_3$), 2.67-2.96 (m, 2H, CH_2), 2.16 (s, 3H, CH_3), 1.78-2.03 (m, 2H, CH_2), 1.24 (s, 9H, *t*-Bu), 1.21 (s, 9H, *t*-Bu).

^{13}C NMR spectrum (100 MHz, DMSO-d_6), δ, ppm: 199.1, 170.3, 168.3, 167.5, 166.2, 165.9, 141.3, 137.0, 136.7, 136.2, 136.1, 133.3, 132.7, 132.5, 132.2, 131.8, 130.5, 130.3, 129.0, 128.6, 128.2, 128.1, 63.3, 62.7, 52.1, 50.6, 50.4, 35.3, 28.4, 28.3, 27.8, 20.5.

Mass spectrum (ESI), m/z: 815 (25) $[M+H]^+$, 813 (23) $[M-H]^-$.

Elemental analysis calcd for $C_{44}H_{48}Cl_2N_4O_7$: C 64.78, H 5.93, N 6.87; found: C 64.72, H 5.98, N 6.96.

Tandem Ugi/aza-Wittig reaction

General procedure for the synthesis of compounds 8a–h. Aldehydes **2a**,**b**,**e** (0.68 mmol) and *o*-azidoanilines **7a**–**c** (0.68 mmol) were dissolved in 2 mL of methanol in a round-bottomed flask and stirred at 25 °C for 30 minutes. Then, α-ketoglutaric acid (**1**, 0.68 mmol, 100 mg) was added to the reaction mixture and stirred for 30 minutes. Then, *tert*-butyl isocyanide (**4**, 0.68 mmol, 77 µL) was added and the reaction mixture was stirred at 45 °C for 24 hours. The completeness of the reaction was determined by thin-layer chromatography (hexane/ethyl acetate 1:2). The reaction mixture was cooled and poured onto ice, the precipitate of compounds **8a**–**h** was filtered and dried in vacuo. If necessary, the crude product was purified by column chromatography, eluting with gradient hexane/ethyl acetate 3:1 to hexane/ethyl acetate 1:2 + 0.1% formic acid.

5-((2-Azido-4-chlorophenyl)(2-(*tert*-butylamino)-1-(4-chlorophenyl)-2-oxoethyl)amino)-4,5-dioxopentanoic acid (8a). Yield 57% (200 mg), white solid, m.p. = 137-139 °C, Rf = 0.62.

^{1}H NMR spectrum (400 MHz, DMSO-d_6), δ, ppm: 12.15 (s, 1H, COOH), 7.95 (s, 1H, NH), 7.02–7.82 (m, 7H, ArH), 5.95 (s, 1H, CH), 2.95 (t, J = 6.2 Hz, 2H, CH_2), 2.32 (t, J = 6.0 Hz, 2H, CH_2), 1.25 (s, 9H, *t*-Bu).

^{13}C NMR spectrum (100 MHz, DMSO-d_6), δ, ppm: 197.8, 172.9, 167.7, 165.6, 139.3, 133.9, 133.7, 133.0, 132.0, 131.9, 127.8, 127.5, 124.7, 118.9, 63.2, 50.6, 33.9, 28.3, 26.5.

Mass spectrum (ESI), m/z: 518 (92) $[M-H]^-$.

Elemental analysis calcd for $C_{23}H_{23}Cl_2N_5O_5$: C 53.09, H 4.46, N 13.46; found: C 52.97, H 4.59, N 13.57.

5-((2-Azidophenyl)(2-(*tert*-butylamino)-1-(4-chlorophenyl)-2-oxoethyl)amino)-4,5-dioxopentanoic acid (8b). Yield 54% (180 mg), white solid, m.p. = 127-129 ˚C, Rf = 0.56.

^{1}H NMR spectrum (400 MHz, DMSO-d_6), δ, ppm: 7.92 (s, 1H, NH), 6.89–7.82 (m, 8H, ArH), 5.91 (s, 1H, CH), 2.91 (t, J = 6.5 Hz, 2H, CH_2), 2.26 (t, J = 6.4 Hz, 2H, CH_2), 1.24 (s, 9H, *t*-Bu).

^{13}C NMR spectrum (100 MHz, DMSO-d_6), δ, ppm: 198.0, 172.9, 167.8, 166.0, 137.8, 132.9, 132.8, 132.2, 132.0, 130.5, 130.2, 128.4, 128.2, 127.6, 124.8, 119.0, 63.4, 50.5, 34.2, 28.3, 26.5.

Mass spectrum (ESI), m/z: 484 (69) $[M-H]^-$.

Elemental analysis calcd for $C_{23}H_{24}ClN_5O_5$: C 56.85, H 4.98, N 14.41; found: C 56.78, H 5.12, N 14.52.

5-((2-Azidophenyl)(2-(*tert*-butylamino)-1-(4-methoxyphenyl)-2-oxoethyl)amino)-4,5-dioxopentanoic acid (8d). Yield 35% (115 mg), white solid, m.p. = 63-65 ˚C (with decomposition), Rf = 0.69.

^{1}H NMR spectrum (400 MHz, DMSO-d_6), δ, ppm: 12.12 (s, 1H, COOH), 7.79 (s, 1H, NH), 6.54-7.33 (m, 8H, ArH), 5.85 (s, 1H, CH), 3.61 (s, 3H, OCH_3), 2.90 (t, J = 6.0 Hz, 2H, CH_2), 2.25 (t, J = 6.1 Hz, 2H, CH_2), 1.24 (s, 9H, *t*-Bu).

^{13}C NMR spectrum (100 MHz, DMSO-d_6), δ, ppm: 198.2, 172.9, 168.4, 166.1, 158.8, 137.8, 132.9, 131.4, 130.0, 128.7, 124.9, 124.6, 119.0, 112.9, 63.7, 55.0, 50.4, 34.1, 28.4, 26.5.

Mass spectrum (ESI), m/z: 480 (100) $[M-H]^-$.

Elemental analysis calcd for $C_{24}H_{27}N_5O_6$: C 59.87, H 5.65, N 14.54; found: C 59.67, H 5.73, N 14.68

5-((2-Azido-4-chlorophenyl)(2-(*tert*-butylamino)-2-oxo-1-phenylethyl)amino)-4,5-dioxopentanoic acid (8f). Yield 78% (255 mg), white solid, m.p. = 129-131 ˚C, Rf = 0.67.

^{1}H NMR spectrum (400 MHz, DMSO-d_6), δ, ppm: 12.15 (s, 1H, COOH), 7.93 (s, 1H, NH), 6.94-7.85 (m, 8H, ArH), 5.96 (s, 1H, CH), 2.87-3.04 (m, 2H, CH_2), 2.31 (t, J = 6.2 Hz, 2H, CH_2), 1.25 (s, 9H, *t*-Bu).

^{13}C NMR spectrum (100 MHz, DMSO-d_6), δ, ppm: 197.9, 172.9, 168.0, 165.6, 139.2, 134.0, 133.5, 133.0, 130.1, 128.3, 127.8, 127.7, 124.5, 118.7, 64.1, 50.5, 33.9, 28.3, 26.5.

Mass spectrum (ESI), m/z: 484 (100) $[M-H]^-$.

Elemental analysis calcd for $C_{23}H_{24}ClN_5O_5$: C 56.85, H 4.98, N 14.41; found: C 56.72, H 5.08, N 14.52

5-((2-Azidophenyl)(2-(*tert*-butylamino)-2-oxo-1-phenylethyl)amino)-4,5-dioxopentanoic acid (8g). Yield 71% (220 mg), white solid, m.p. = 121-123 ˚C (with decomposition), Rf = 0.55.

^{1}H NMR spectrum (400 MHz, DMSO-d_6), δ, ppm: 12.15 (s, 1H, COOH), 7.87 (s, 1H, NH), 6.76-7.84 (m, 9H, ArH), 5.93 (s, 1H, CH), 2.92 (t, J = 6.4 Hz, 2H, CH_2), 2.26 (t, J = 6.4 Hz, 2H, CH_2), 1.25 (s, 9H, *t*-Bu).

^{13}C NMR spectrum (100 MHz, DMSO-d_6), δ, ppm: 198.0, 172.8, 168.0, 166.0, 137.7, 133.0, 132.8, 130.1, 129.9, 128.6, 128.0, 127.4, 124.6, 118.8, 64.2, 50.4, 34.1, 28.3, 26.5.

Mass spectrum (ESI), m/z: 450 (100) $[M-H]^-$.

Elemental analysis calcd for $C_{23}H_{25}N_5O_5$: C 61.19, H 5.58, N 15.51; found: C 61.07, H 5.64, N 15.62

5-((2-Azido-4,6-dimethylphenyl)(2-(*tert*-butylamino)-2-oxo-1-phenylethyl)amino)-4,5-dioxopentanoic acid (8h). Mixture of rotamers. Yield 64% (210 mg), white solid, m.p. = 83-85 ˚C, Rf = 0.66.

^{1}H NMR spectrum (400 MHz, DMSO-d_6), δ, ppm: 12.11 (s, 1H, COOH), 7.94 (s, 1H, NH), 7.72 (s, 1H, NH), 6.50-7.54 (m, 7H, ArH), 5.43-5.75 (m, 1H, CH), 2.60-3.06 (m, 2H, CH_2), 2.42 (s, 3H, CH_3), 2.14-2.35 (m, 2H, CH_2), 2.18 (s, 3H, CH_3), 2.09 (s, 3H, CH_3), 2.00 (s, 3H, CH_3) 1.20 (s, 9H, *t*-Bu).

^{13}C NMR spectrum (100 MHz, DMSO-d_6), δ, ppm: 198.0, 172.8, 167.9, 167.5, 167.1, 166.5, 140.6, 139.9, 139.3, 138.3, 138.2, 134.5, 133.3, 129.4, 128.9, 128.2, 128.0, 127.8, 127.7, 127.5, 127.4, 125.1, 116.8, 116.2, 66.5, 65.8, 50.1, 50.0, 33.7, 28.3, 28.1, 26.5, 20.5, 20.4, 18.4, 17.9.

Mass spectrum (ESI), m/z: 478 (100) $[M-H]^{-}$.

Elemental analysis calcd for $C_{25}H_{29}N_5O_5$: C 62.62, H 6.10, N 14.60; found: C 62.51, H 6.17, N 14.71

General procedure for the synthesis of compounds 9a–h.

The azidated derivatives **8a**–**h** (0.2 mmol) were dissolved in 3 mL of dichloromethane in a round-bottomed flask. Triphenylphosphine (0.2 mmol, 52 mg) was added to the solution and the reaction mixture was stirred at 20 °C for 12 hours. Then, the solvent was evaporated under vacuum and the crude product of compounds **9a**–**h** was purified by column chromatography, eluting with gradient hexane/ethyl acetate 3:1 to hexane/ethyl acetate 1:2 + 0.1% formic acid.

General procedure for the one-pot synthesis of compounds 9c–e

The corresponding benzaldehyde **2a** or **2b** (0.68 mmol) and *o*-azidoaniline **7b**,**c** (0.68 mmol) were dissolved in 2 mL of methanol in a round-bottomed flask and stirred at 25 °C for 30 minutes. Then, α-ketoglutaric acid (**1**, 0.68 mmol, 100 mg) was added to the reaction mixture and stirred for 30 minutes. Then, *tert*-butyl isocyanide (**4**, 0.68 mmol, 77 μL) was added and the reaction mixture was stirred at 45 °C for 24 hours. Then, the solvent was evaporated, and the dry residue was dissolved in 4 mL of dichloromethane. Triphenylphosphine (0.68 mmol, 180 mg) was added to the solution and the reaction mixture was stirred at 20 °C for 12 hours. Then, the solvent was evaporated under vacuum and the crude product of compounds **9c**–**e** was purified by column chromatography, elution gradient hexane/ethyl acetate 3:1 to hexane/ethyl acetate 1:2 + 0.1% formic acid.

3-(4-(2-(*tert*-Butylamino)-1-(4-chlorophenyl)-2-oxoethyl)-7-chloro-3-oxo-3,4-dihydroquinoxalin-2-yl)propanoic acid (9a). Yield 82% (80 mg), white solid, m.p. = 78-80 ˚C, Rf = 0.67 (hexane/ethyl acetate 1:2 + 0.1% formic acid).

^{1}H NMR spectrum (400 MHz, DMSO-d_6), δ, ppm: 7.99 (s, 1H, NH), 7.18-7.76 (m, 7H, ArH), 6.86 (s, 1H, CH), 3.12 (t, J = 7.0 Hz, 2H, CH_2), 2.75 (t, J = 7.0 Hz, 2H, CH_2), 1.26 (s, 9H, *t*-Bu).

^{13}C NMR spectrum (100 MHz, DMSO-d_6), δ, ppm: 173.9, 165.2, 160.5, 154.4, 134.2, 133.2, 132.3, 130.9, 129.8, 128.4, 128.1, 127.7, 127.2, 119.0, 59.2, 51.1, 29.5, 28.4.

Mass spectrum (ESI), m/z: 474 (40) $[M-H]^-$.

Elemental analysis calcd for $C_{23}H_{23}Cl_2N_3O_4$: C 57.99, H 4.87, N 8.82; found: C 57.89; H 4.96; N 8.89.

3-(4-(2-(*tert*-Butylamino)-1-(4-chlorophenyl)-2-oxoethyl)-3-oxo-3,4-dihydroquinoxalin-2-yl)propanoic acid (9b). Yield 93% (80 mg), white solid, m.p. = 62-64 ˚C, Rf = 0.46 (hexane/ethyl acetate 1:2 + 0.1% formic acid).

^{1}H NMR spectrum (400 MHz, DMSO-d_6), δ, ppm: 7.86 (s, 1H, NH), 7.21-7.75 (m, 8H, ArH), 6.84 (s, 1H, CH), 3.12 (t, J = 6.5 Hz, 2H, CH_2), 2.76 (t, J = 7.0 Hz, 2H, CH_2), 1.25 (s, 9H, *t*-Bu).

^{13}C NMR spectrum (100 MHz, DMSO-d_6), δ, ppm: 174.0, 165.3, 158.8, 154.6, 134.5, 132.5, 132.2, 131.9, 130.0, 129.0, 128.5, 128.3, 123.3, 117.0, 59.2, 51.1, 29.6, 28.4.

Mass spectrum (ESI), m/z: 440 (39) $[M-H]^-$.

Elemental analysis calcd for $C_{23}H_{24}ClN_3O_4$: C 62.51, H 5.47, N 9.51; found: C 62.42; H 5.57; N 9.62

3-(4-(2-(*tert*-Butylamino)-1-(4-chlorophenyl)-2-oxoethyl)-5,7-dimethyl-3-oxo-3,4-dihydroquinoxalin-2-yl)propanoic acid (9c). Yield 46% (45 mg) in terms of KGA using the one-pot method, white solid, m.p. = 184-186 ˚C (decomposition), Rf = 0.62 (hexane/ethyl acetate 1:2 + 0.1% formic acid).

^{1}H NMR spectrum (400 MHz, DMSO-d_6), δ, ppm: 12.11 (s, 1H, COOH), 7.42 (s, 1H, NH), 6.74-7.41 (m, 6H, ArH), 6.10 (s, 1H, CH), 2.90 (t, J = 7.3 Hz, 2H, CH_2), 2.63 (t, J = 6.6 Hz, 2H, CH_2), 2.41 (s, 3H, CH_3), 2.34 (s, 3H, CH_3), 1.25 (s, 9H, *t*-Bu).

^{13}C NMR spectrum (100 MHz, DMSO-d_6), δ, ppm: 173.8, 165.9, 159.0, 156.1, 135.4, 135.0, 134.1, 132.9, 132.4, 131.9, 130.7, 127.8, 127.4, 124.5, 66.7, 51.0, 29.4, 28.3, 27.8, 21.8, 19.7.

Mass spectrum (ESI), m/z: 468 (100) $[M-H]^-$.

Elemental analysis calcd for $C_{25}H_{28}ClN_3O_4$: C 63.89, H 6.01, N 8.94; found: C 63.76, H 6.05, N 8.98

3-(4-(2-(*tert*-Butylamino)-1-(4-methoxyphenyl)-2-oxoethyl)-3-oxo-3,4-dihydroquinoxalin-2-yl)propanoic acid (9d). Yield 75% (65 mg),51% (150 mg) in terms of KGA using the one-pot method, white solid, m.p. = 77-79 ˚C, Rf = 0.48 (hexane/ethyl acetate 1:2 + 0.1% formic acid).

^{1}H NMR spectrum (400 MHz, DMSO-d_6), δ, ppm: 12.15 (s, 1H, COOH), 7.78 (s, 1H, NH), 6.67-7.74 (m, 9H, ArH+CH), 3.71 (s, 3H, OCH_3), 3.12 (t, *J* = 6.1 Hz, 2H, CH_2), 2.76 (t, *J* = 7.1 Hz, 2H, CH_2), 1.28 (s, 9H, *t*-Bu).

^{13}C NMR spectrum (100 MHz, DMSO-d_6), δ, ppm: 173.8, 165.9, 158.6, 158.5, 154.6, 132.4, 131.9, 129.2, 128.7, 128.2, 127.4, 123.0, 117.5, 113.8, 59.4, 55.0, 50.9, 29.6, 28.4.

Mass spectrum (ESI), m/z: 436 (100) [M-H]$^-$.

Elemental analysis calcd for $C_{24}H_{27}N_3O_5$: C 65.89, H 6.22, N 9.60; found: C 65.77, H 6.28, N 9.71

3-(4-(2-(*tert*-Butylamino)-1-(4-methoxyphenyl)-2-oxoethyl)-5,7-dimethyl-3-oxo-3,4-dihydroquinoxalin-2-yl)propanoic acid (9e). Yield 35% (110 mg) in terms of KGA using the one-pot method, white solid, m.p. = 152-154 ˚C, Rf = 0.56 (hexane/ethyl acetate 1:2 + 0.1% formic acid).

^{1}H NMR spectrum (400 MHz, DMSO-d_6), δ, ppm: 12.10 (s, 1H, COOH), 7.41 (s, 1H, NH), 6.40-7.29 (m, 6H, ArH), 6.00 (s, 1H, CH), 3.74 (s, 3H, OCH_3), 2.91 (t, *J* = 7.2 Hz, 2H, CH_2), 2.63 (br t, 2H, CH_2), 2.41 (s, 3H, CH_3), 2.34 (s, 3H, CH_3), 1.24 (s, 9H, *t*-Bu).

^{13}C NMR spectrum (100 MHz, DMSO-d_6), δ, ppm: 173.7, 166.7, 159.0, 158.4, 156.2, 134.9, 134.0, 132.7, 132.6, 130.0, 127.9, 127.2, 124.6, 113.4, 67.5, 55.0, 50.8, 29.5, 28.2, 27.9, 21.8, 19.7.

Mass spectrum (ESI), m/z: 464 (100) [M-H]$^-$.

Elemental analysis calcd for $C_{26}H_{31}N_3O_5$: C 67.08, H 6.71, N 9.03; found: C 67.01, H 6.74, N 9.11

3-(4-(2-(*tert*-Butylamino)-2-oxo-1-phenylethyl)-7-chloro-3-oxo-3,4-dihydroquinoxalin-2-yl)propanoic acid (9f). Yield 83% (75 mg), white solid, m.p. = 97-99 ˚C, Rf = 0.62 (hexane/ethyl acetate 1:2 + 0.1% formic acid).

^{1}H NMR spectrum (400 MHz, DMSO-d_6), δ, ppm: 12.17 (s, 1H, COOH), 8.07 (s, 1H, NH), 7.11-7.77 (m, 8H, ArH), 6.98 (s, 1H, CH), 3.14 (t, *J* = 7.1 Hz, 2H, CH_2), 2.76 (t, *J* = 7.1 Hz, 2H, CH_2), 1.29 (s, 9H, *t*-Bu).

^{13}C NMR spectrum (100 MHz, DMSO-d_6), δ, ppm: 173.8, 165.5, 160.3, 154.5, 135.4, 133.2, 130.9, 128.5, 127.8, 127.6, 127.0, 119.6, 59.7, 51.1, 29.5, 28.5, 28.4.

Mass spectrum (ESI), m/z: 440 (100) $[M-H]^-$.

Elemental analysis calcd for $C_{23}H_{24}ClN_3O_4$: C 62.51, H 5.47, N 9.51; found: C 62.42, H 5.52, N 9.59

3-(4-(2-(*tert*-Butylamino)-2-oxo-1-phenylethyl)-3-oxo-3,4-dihydroquinoxalin-2-yl)propanoic acid (9g). Yield 59% (50 mg), white solid, m.p. = 78-80 ˚C, Rf = 0.69 (hexane/ethyl acetate 1:2 + 0.1% formic acid).

^{1}H NMR spectrum (400 MHz, DMSO-d_6), δ, ppm: 12.15 (s, 1H, COOH), 7.92 (s, 1H, NH), 7.12-7.76 (m, 9H, ArH), 6.95 (s, 1H, CH), 3.12 (t, *J* = 6.9 Hz, 2H, CH_2), 2.77 (t, *J* = 7.0 Hz, 2H, CH_2), 1.28 (s, 9H, *t*-Bu).

^{13}C NMR spectrum (100 MHz, DMSO-d_6), δ, ppm: 173.8, 165.6, 158.6, 154.6, 135.6, 132.4, 131.9, 128.7, 128.4, 128.1, 127.7, 127.4, 123.1, 117.6, 59.7, 51.0, 29.6, 28.4.

Mass spectrum (ESI), m/z: 406 (100) $[M-H]^-$.

Elemental analysis calcd for $C_{23}H_{25}N_3O_4$: C 67.80, H 6.18, N 10.31; found: C 67.73, H 6.37, N 10.36

3-(4-(2-(*tert*-Butylamino)-2-oxo-1-phenylethyl)-5,7-dimethyl-3-oxo-3,4-dihydroquinoxalin-2-yl)propanoic acid (9h). Yield 47% (40 mg), white solid, m.p. = 186-188˚C, Rf = 0.68 (hexane/ethyl acetate 1:2 + 0.1% formic acid).

^{1}H NMR spectrum (400 MHz, DMSO-d_6), δ, ppm: 12.10 (s, 1H, COOH), 7.42 (s, 1H, NH), 6.56-7.39 (m, 7H, ArH), 6.09 (s, 1H, CH), 2.91 (t, *J* = 7.2 Hz, 2H, CH_2), 2.63 (br t, 2H, CH_2), 2.40 (s, 3H, CH_3), 2.34 (s, 3H, CH_3), 1.24 (s, 9H, *t*-Bu).

^{13}C NMR spectrum (100 MHz, DMSO-d_6), δ, ppm: 173.8, 166.3, 159.1, 156.2, 136.3, 135.0, 134.0, 132.8, 132.6, 128.6, 128.0, 127.4, 127.3, 124.6, 67.7, 50.9, 29.5, 28.3, 27.9, 21.9, 19.7.

Mass spectrum (ESI), m/z: 434 (100) $[M-H]^{-}$.

Elemental analysis calcd for $C_{25}H_{29}N_3O_4$: C 68.95, H 6.71, N 9.65; found: C 68.82, H 6.80, N 9.73

^{1}H NMR and ^{13}C NMR spectra

^{1}H NMR spectrum of compound **5a**

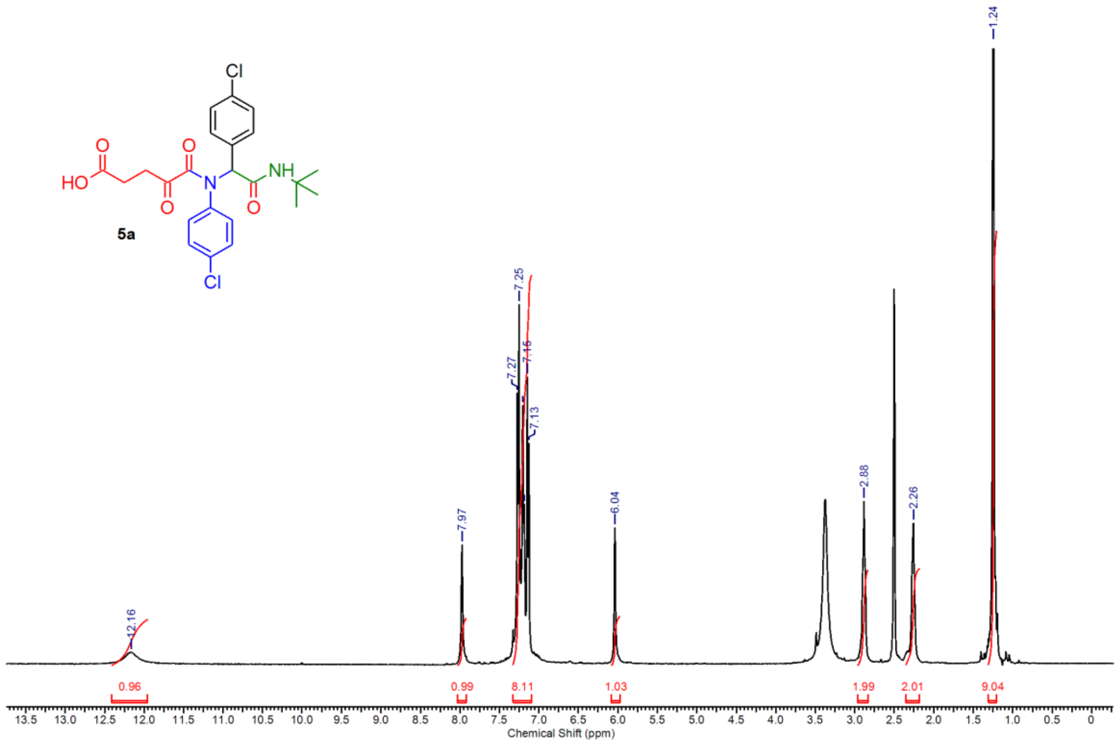

^{13}C NMR spectrum of compound **5a**

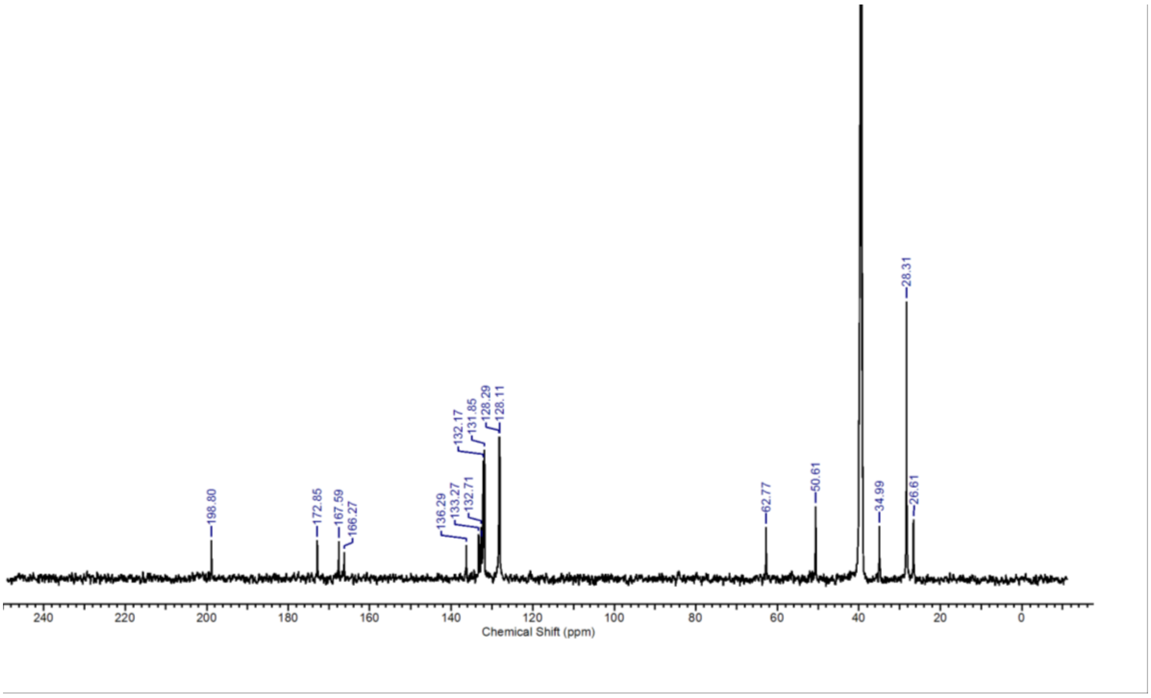

^{1}H NMR spectrum of compound **5b**

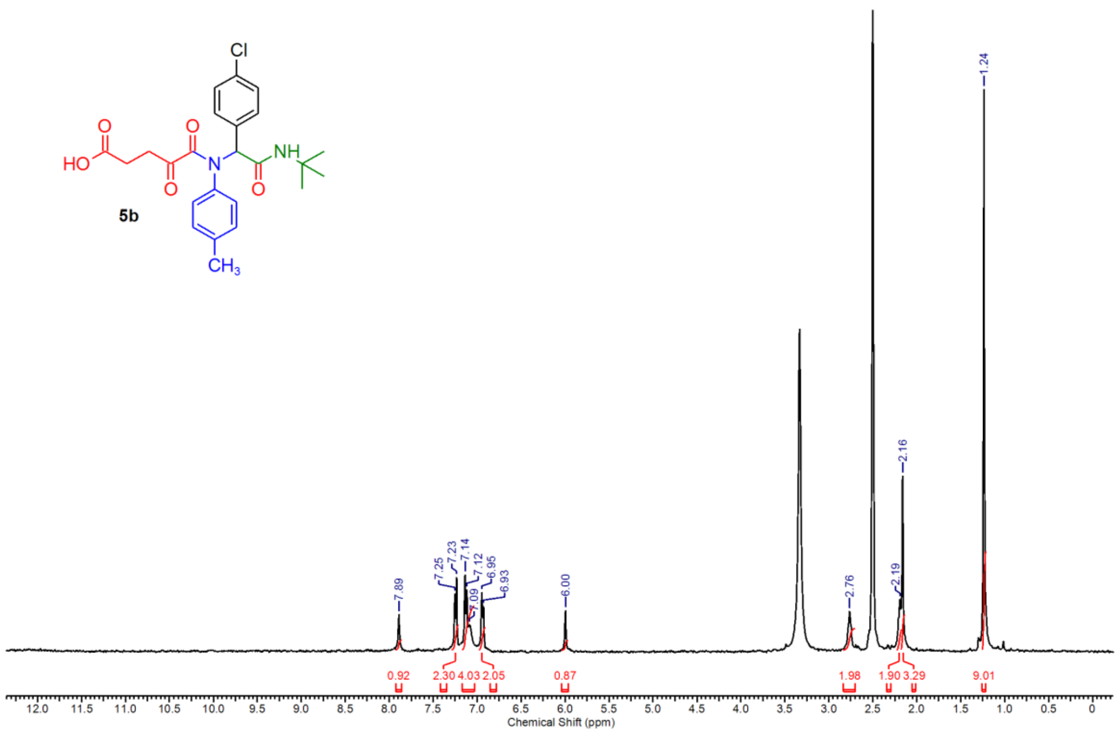

^{13}C NMR spectrum of compound **5b**

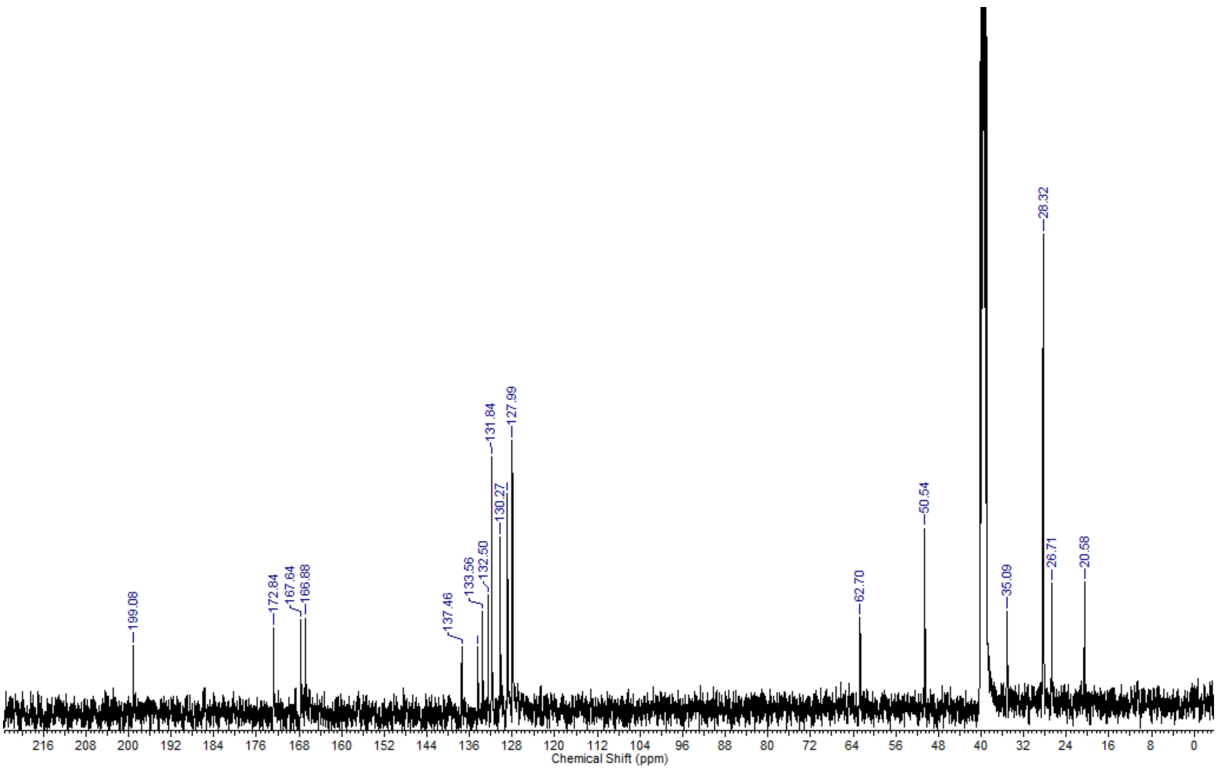

^{1}H NMR spectrum of compound **5c**

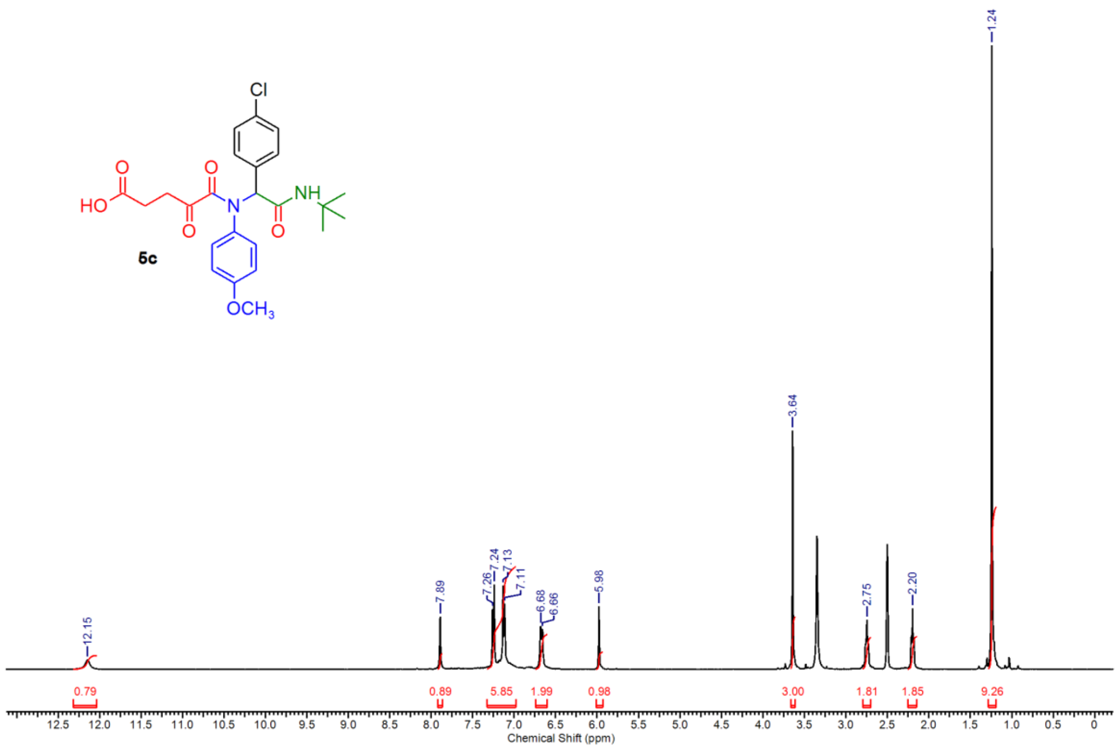

^{13}C NMR spectrum of compound **5c**

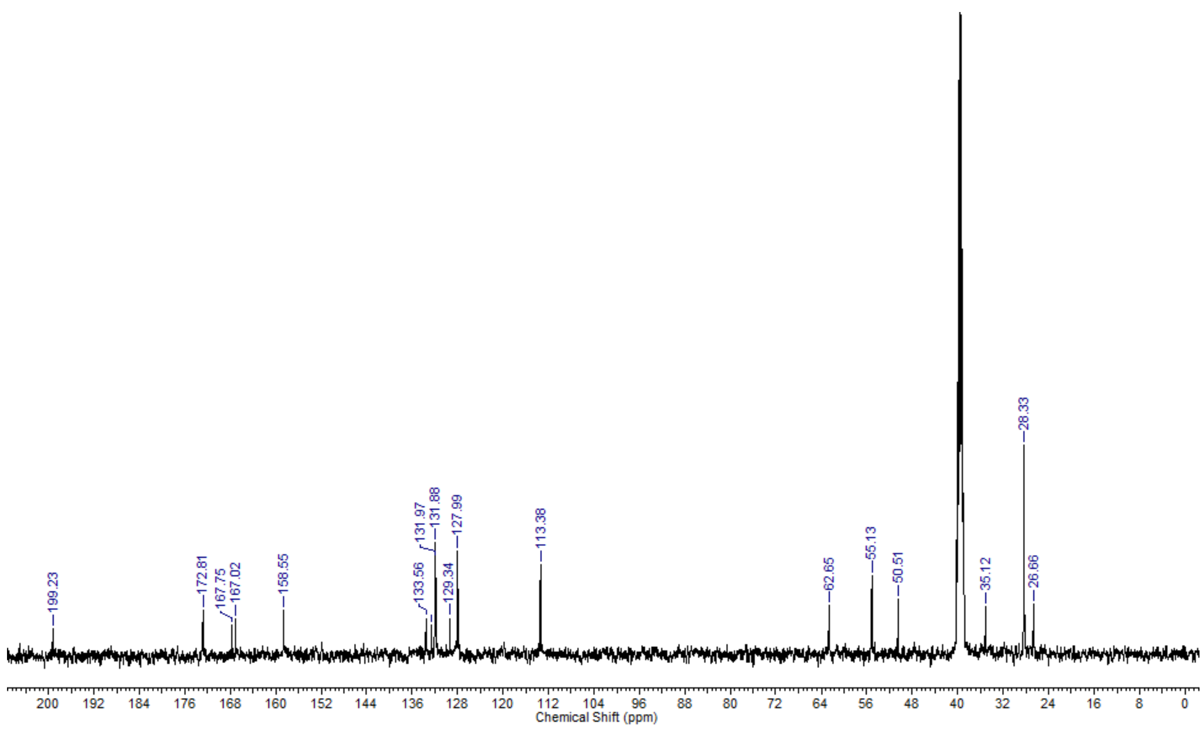

^{1}H NMR spectrum of compound **5d**

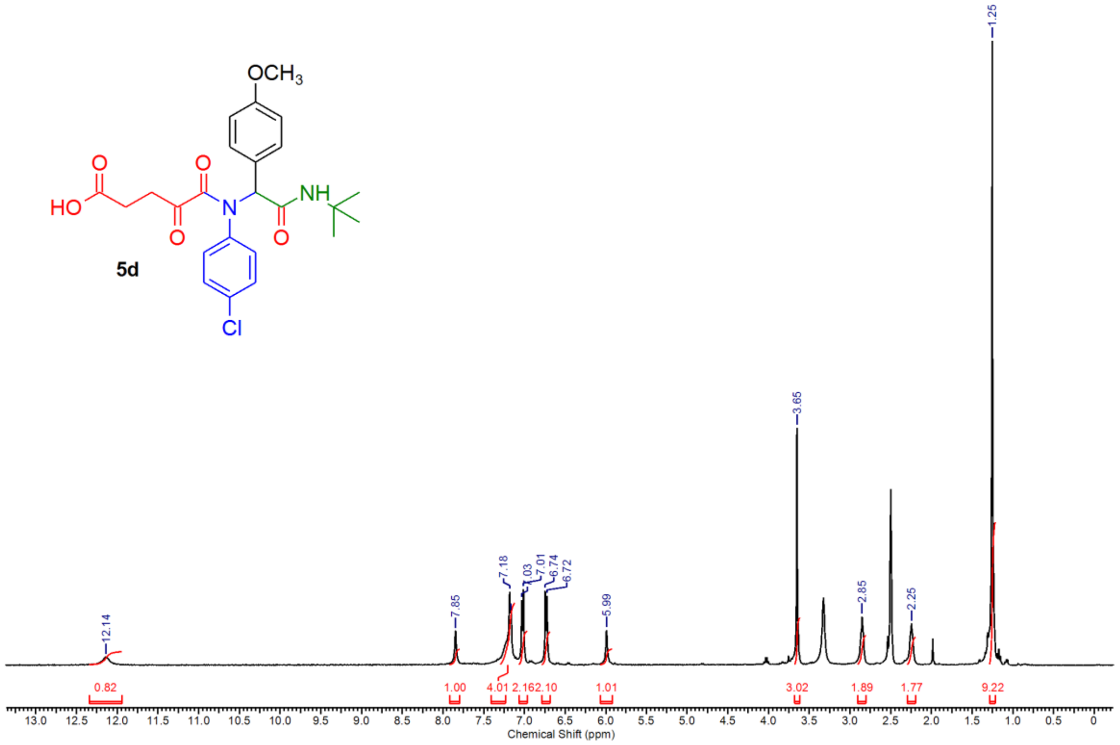

^{13}C NMR spectrum of compound **5d**

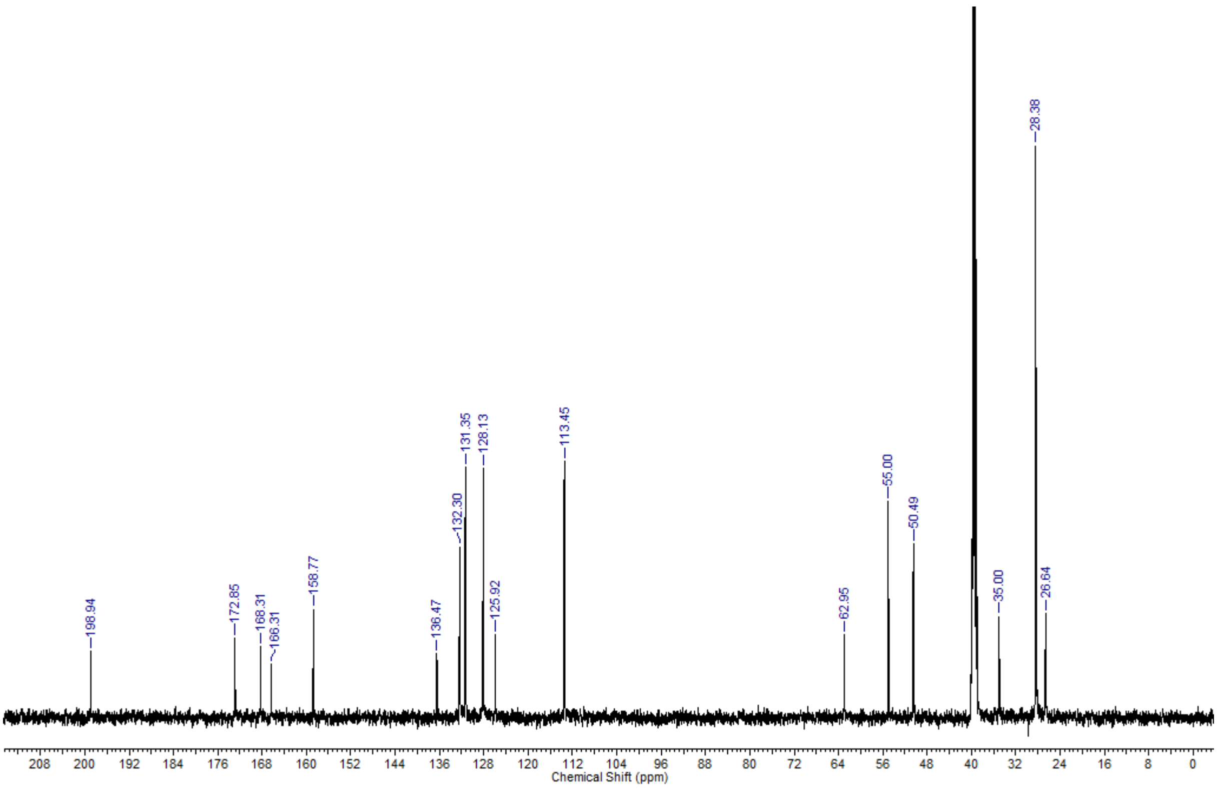

^{1}H NMR spectrum of compound **5e**

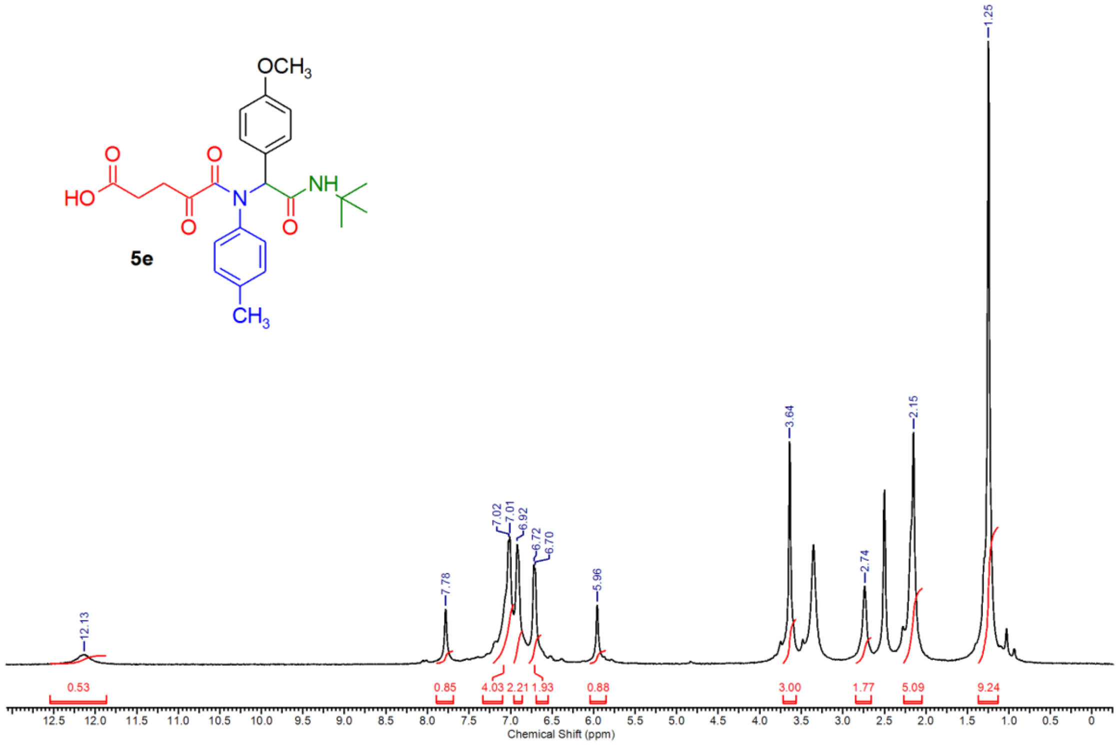

^{13}C NMR spectrum of compound **5e**

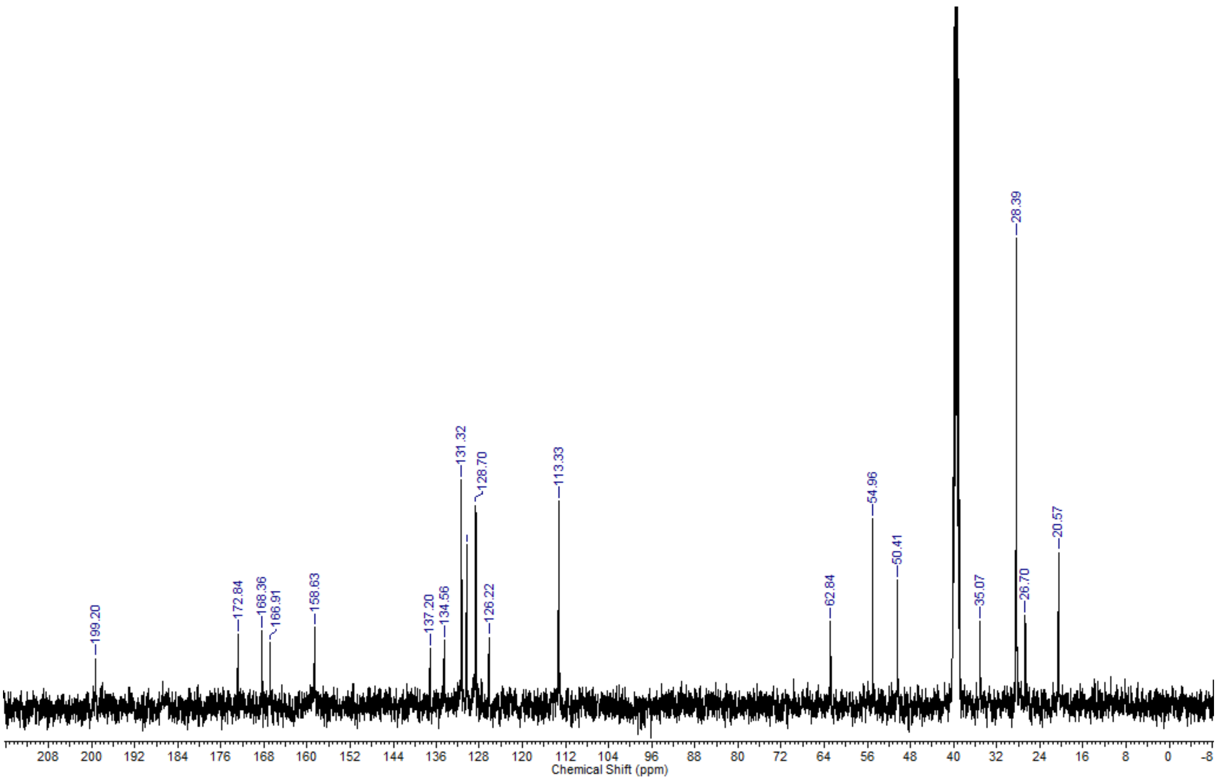

^{1}H NMR spectrum of compound **5f**

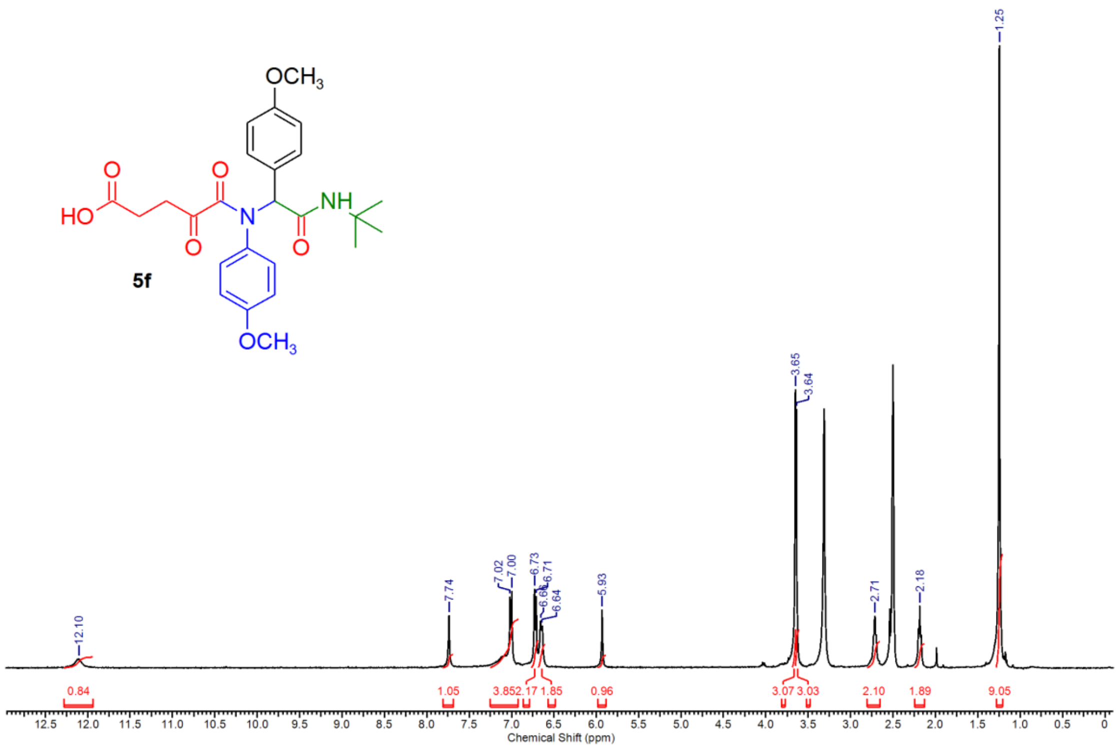

^{13}C NMR spectrum of compound **5f**

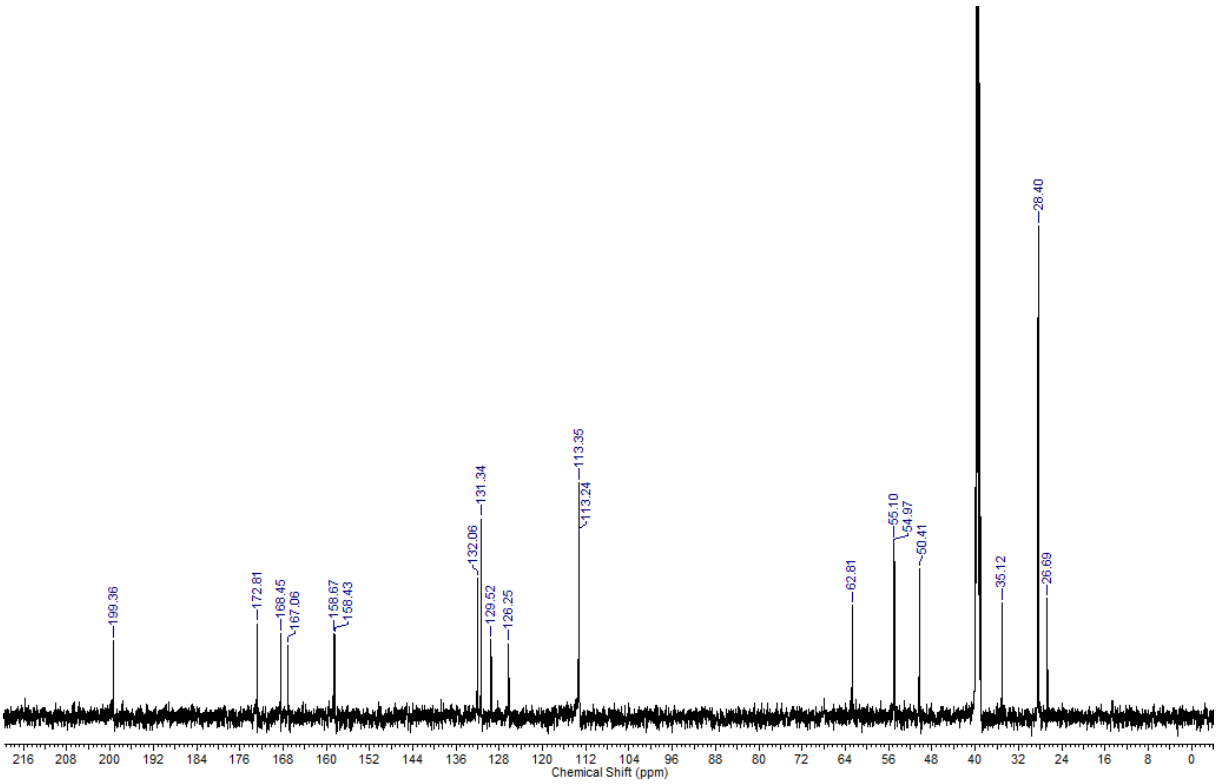

^{1}H NMR spectrum of compound **5g**

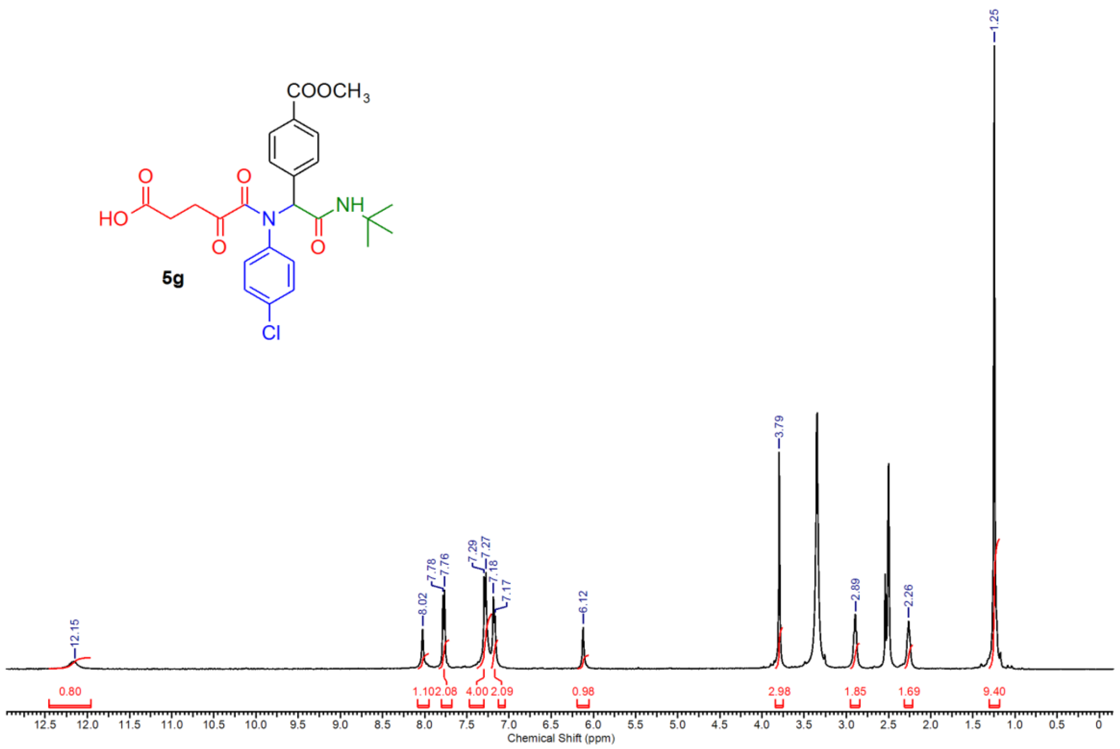

^{13}C NMR spectrum of compound **5g**

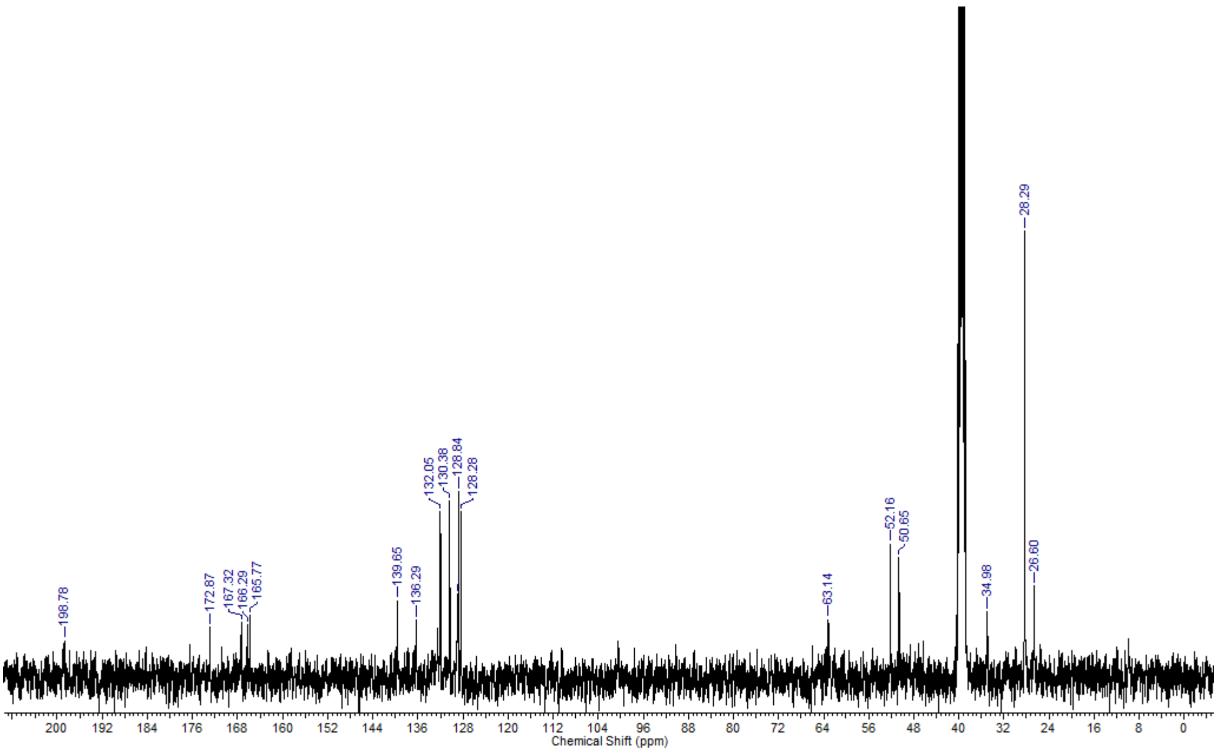

^{1}H NMR spectrum of compound **5h**

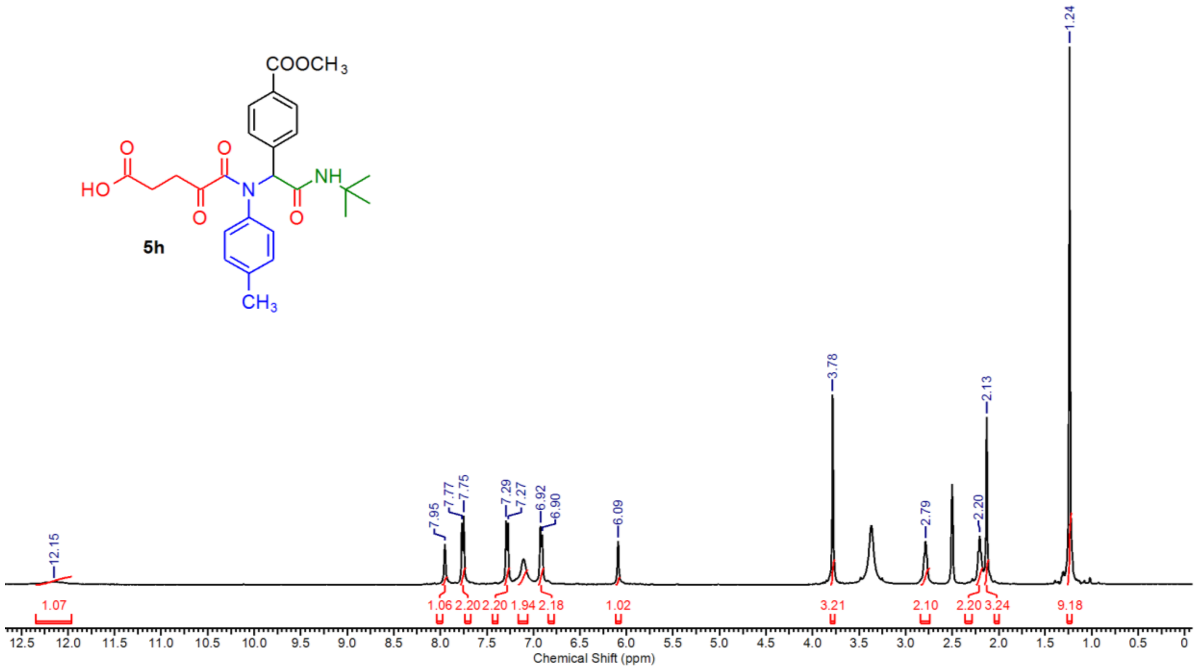

^{13}C NMR spectrum of compound **5h**

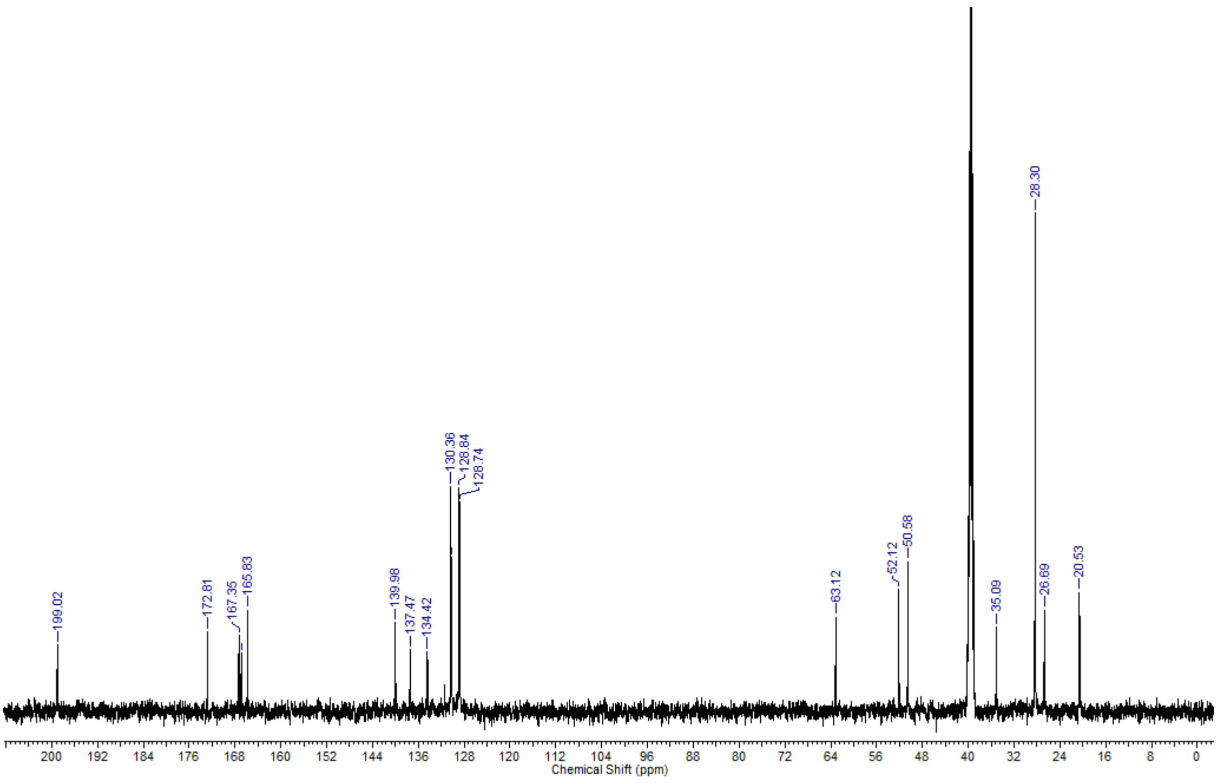

^{1}H NMR spectrum of compound **5i**

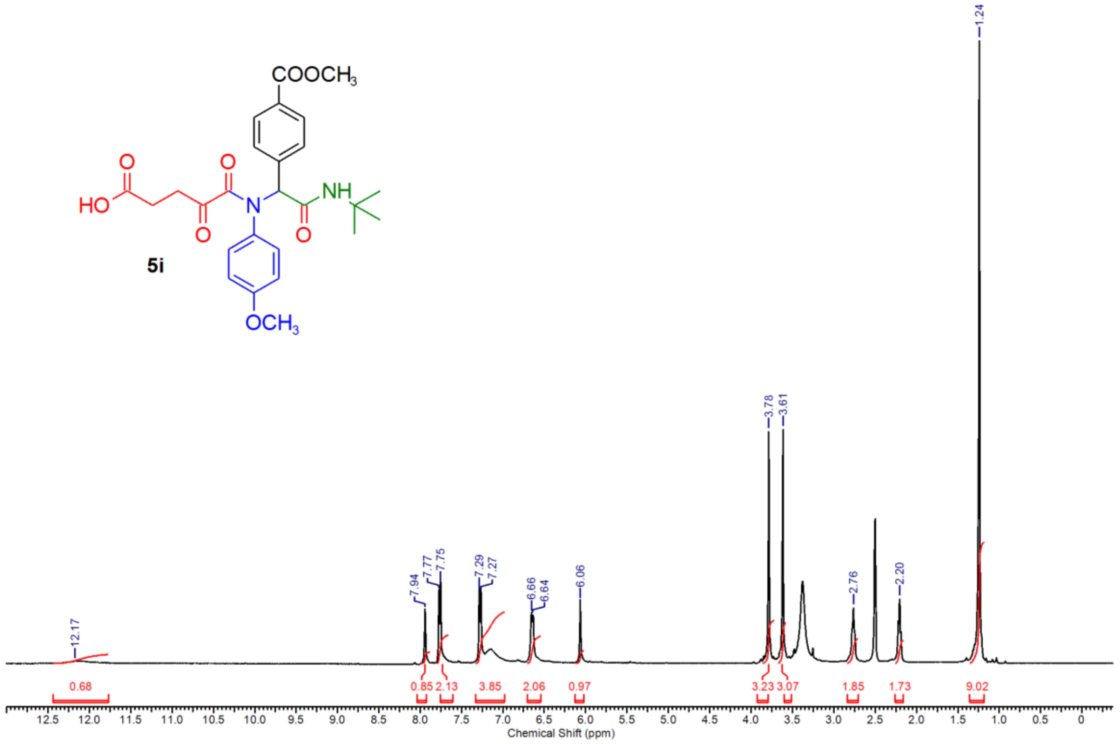

^{13}C NMR spectrum of compound **5i**

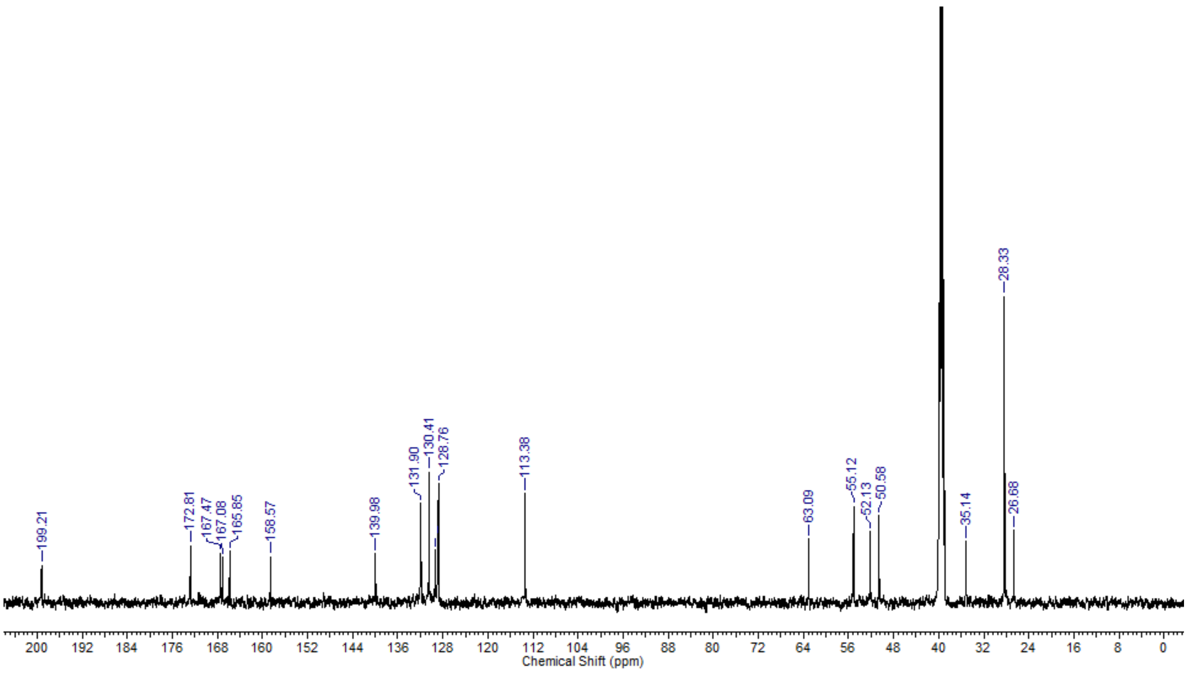

^{1}H NMR spectrum of compound **5j**

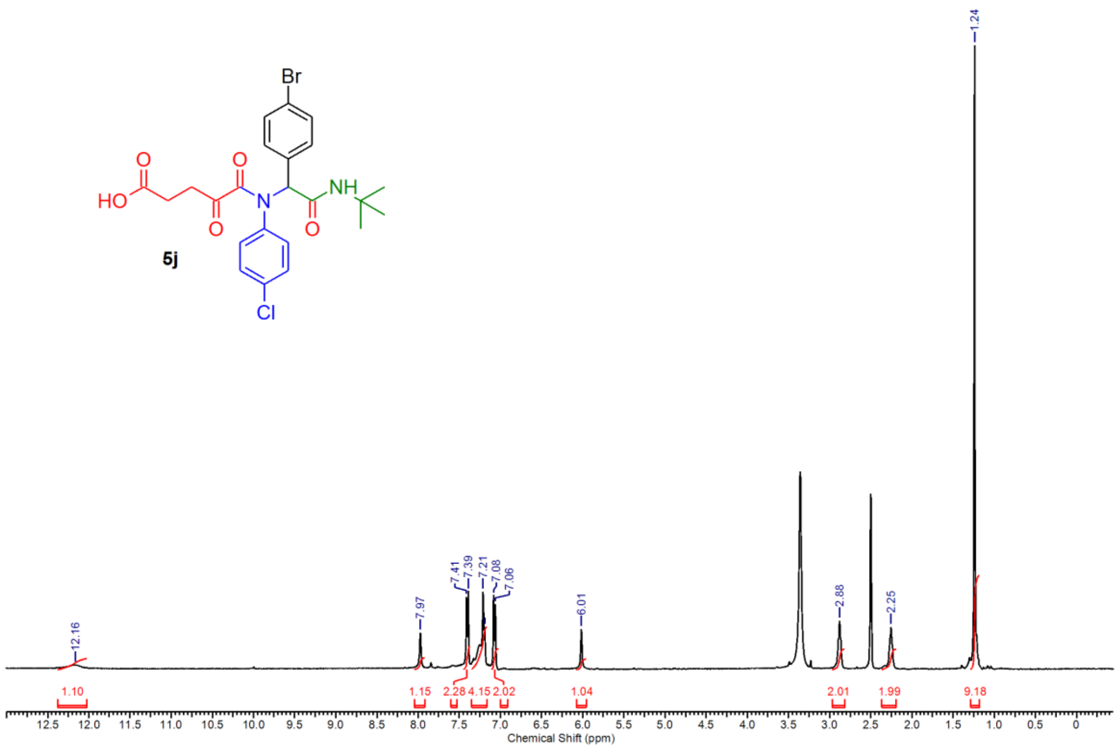

^{13}C NMR spectrum of compound **5j**

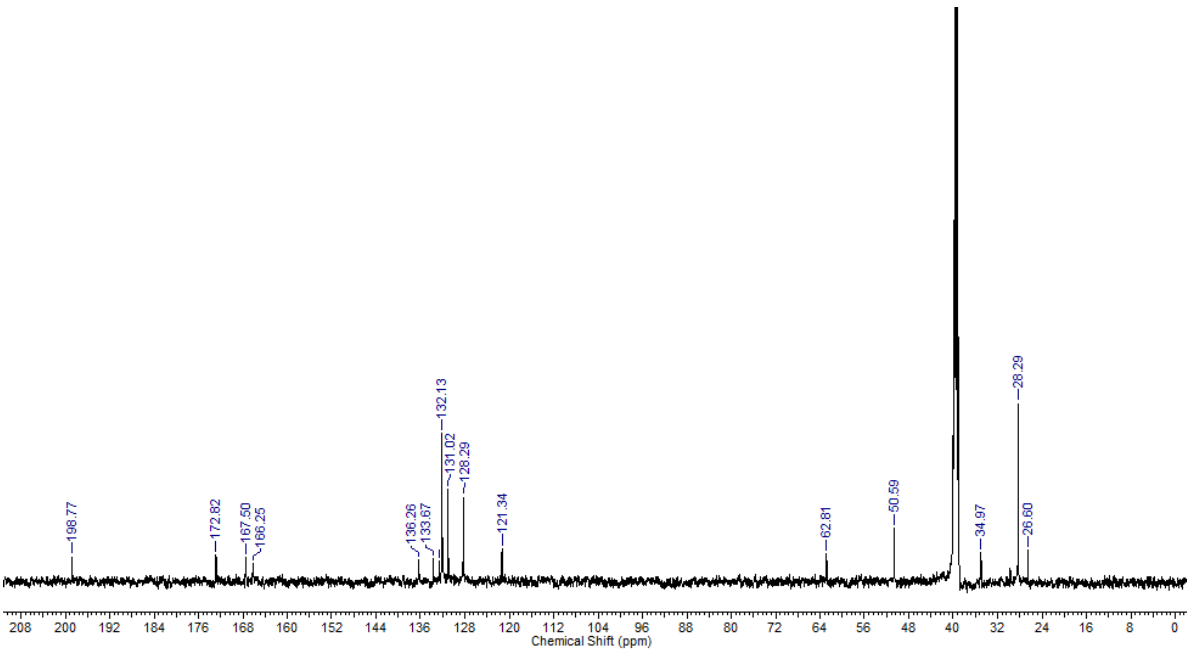

^{1}H NMR spectrum of compound **5k**

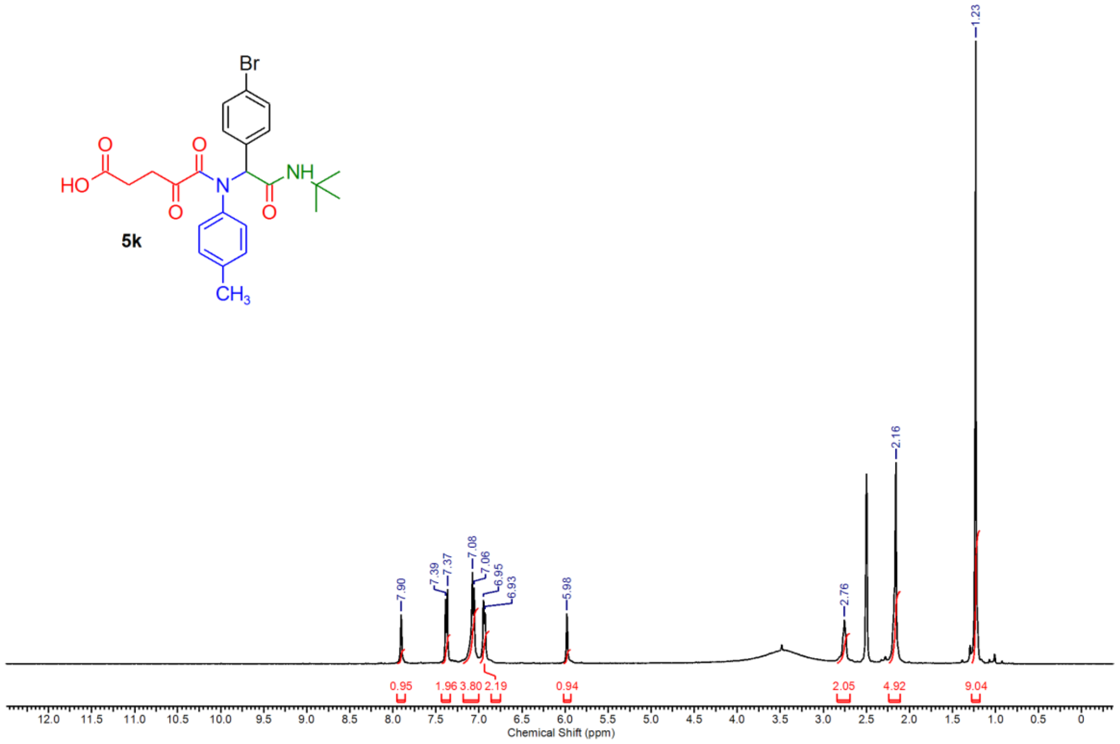

^{13}C NMR spectrum of compound **5k**

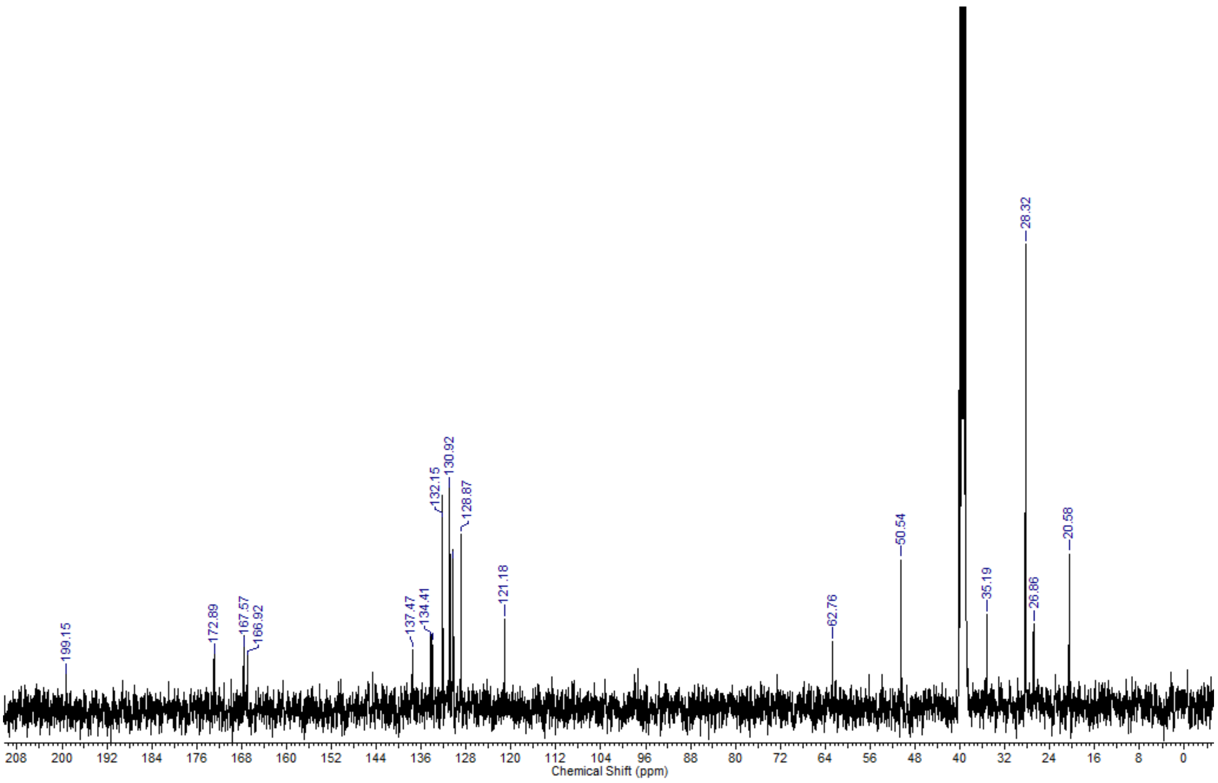

^{1}H NMR spectrum of compound **5l**

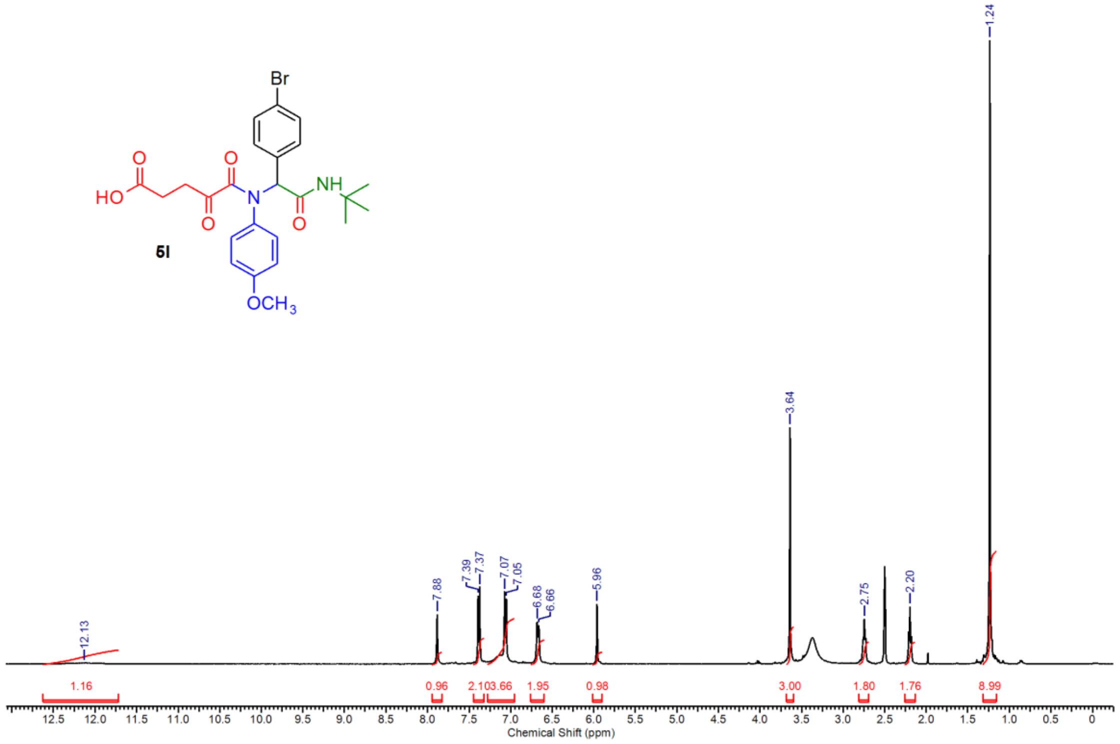

^{13}C NMR spectrum of compound **5l**

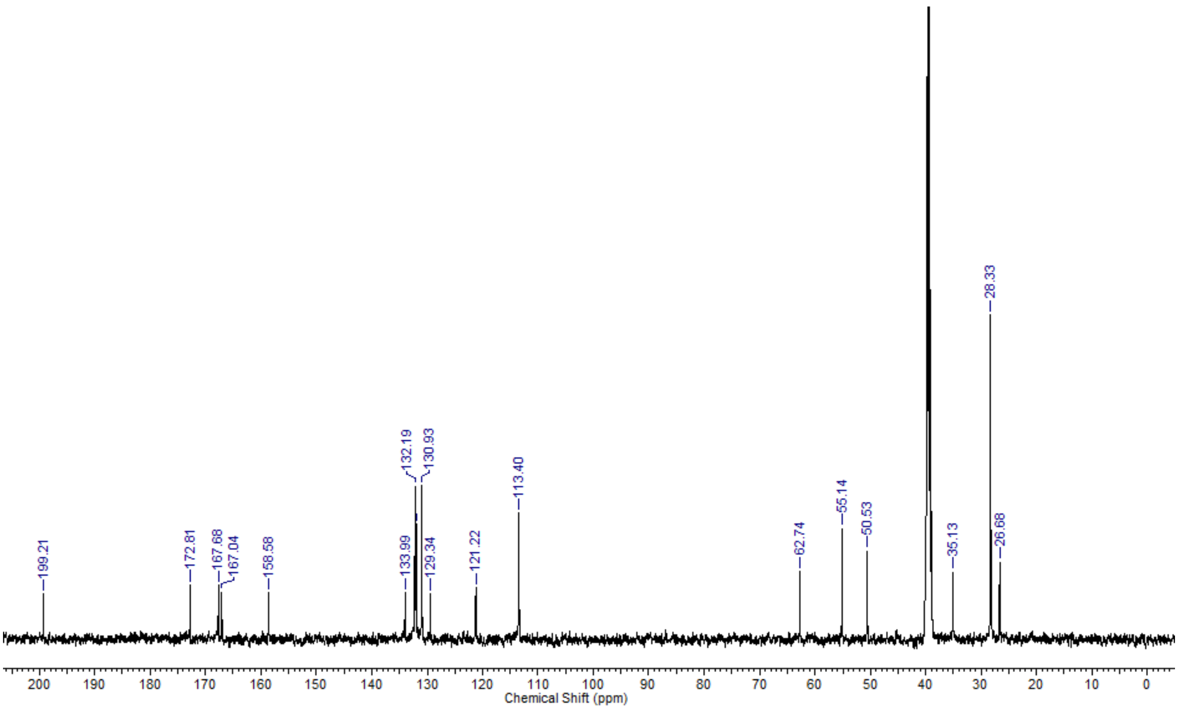

^{1}H NMR spectrum of compound **6a**

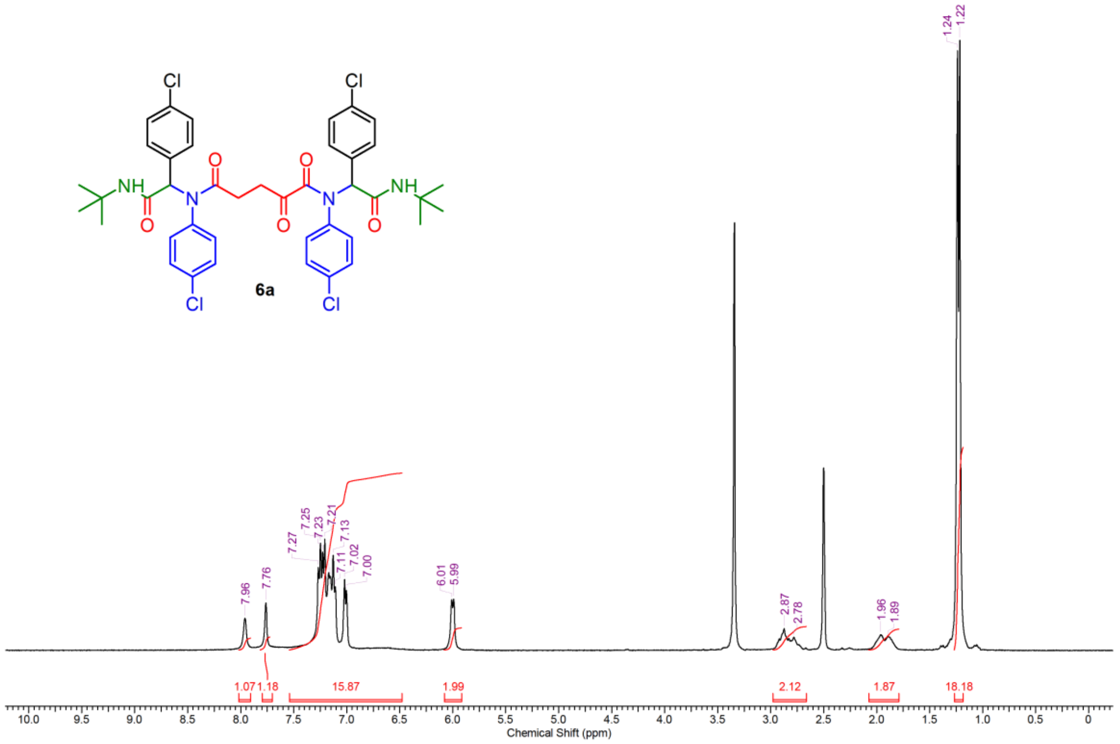

^{13}C NMR spectrum of compound **6a**

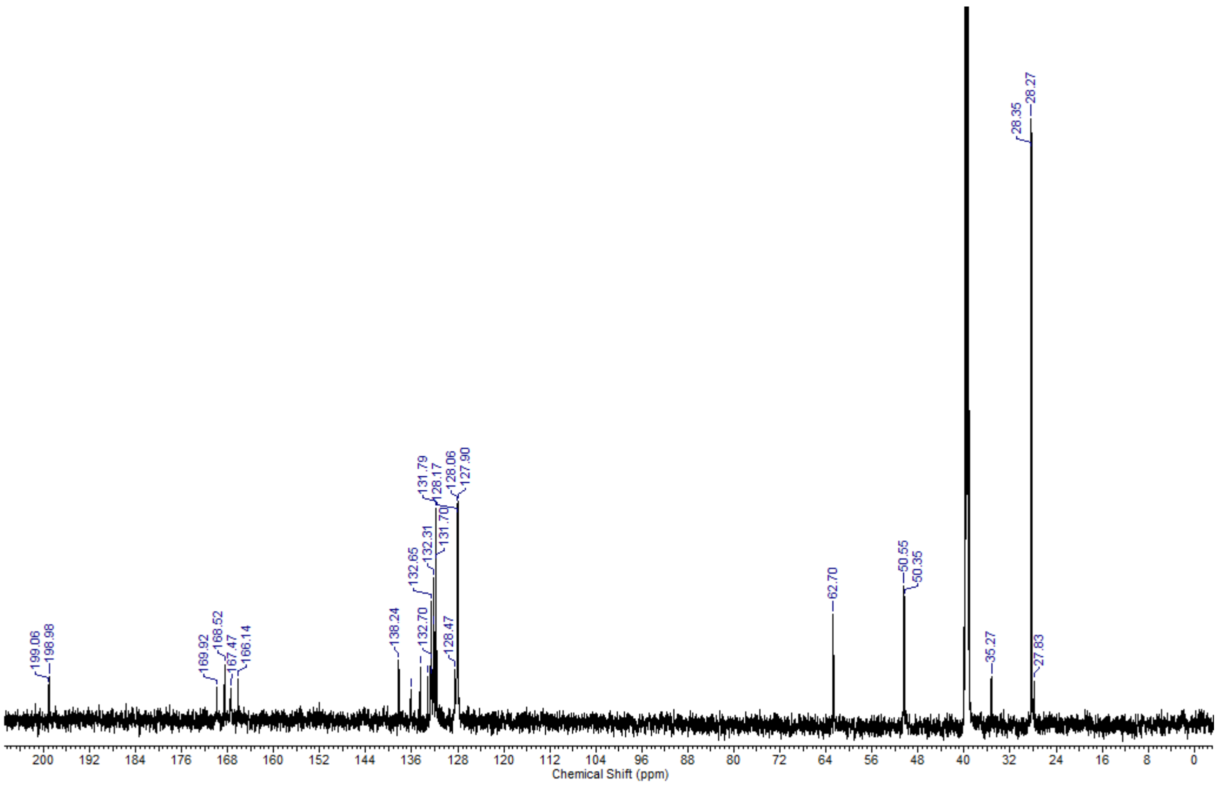

^{1}H NMR spectrum of compound **6b**

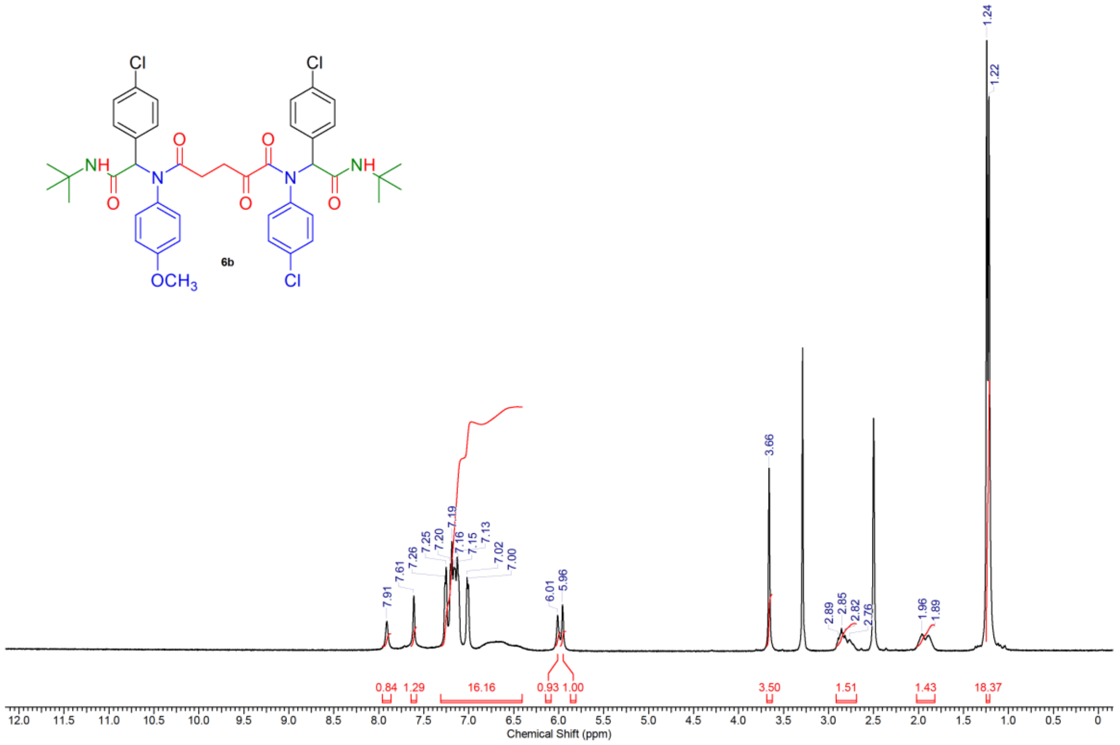

^{13}C NMR spectrum of compound **6b**

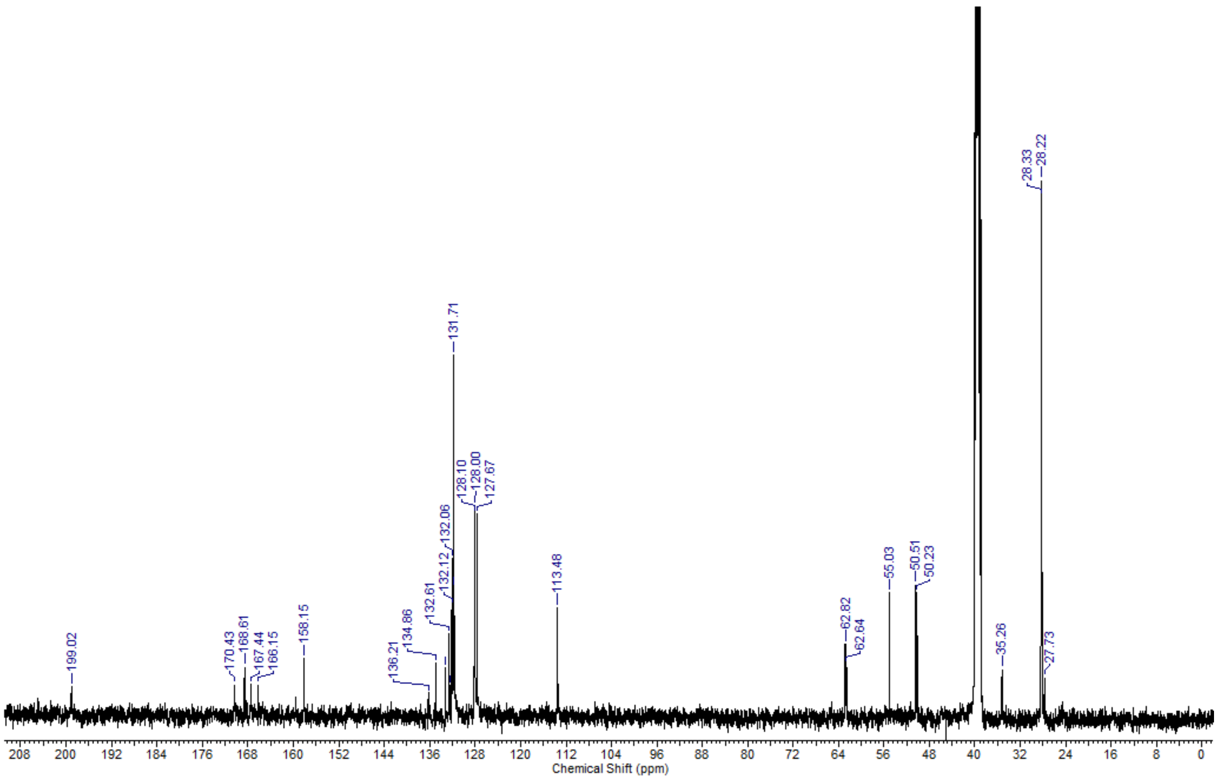

^{1}H NMR spectrum of compound **6c**

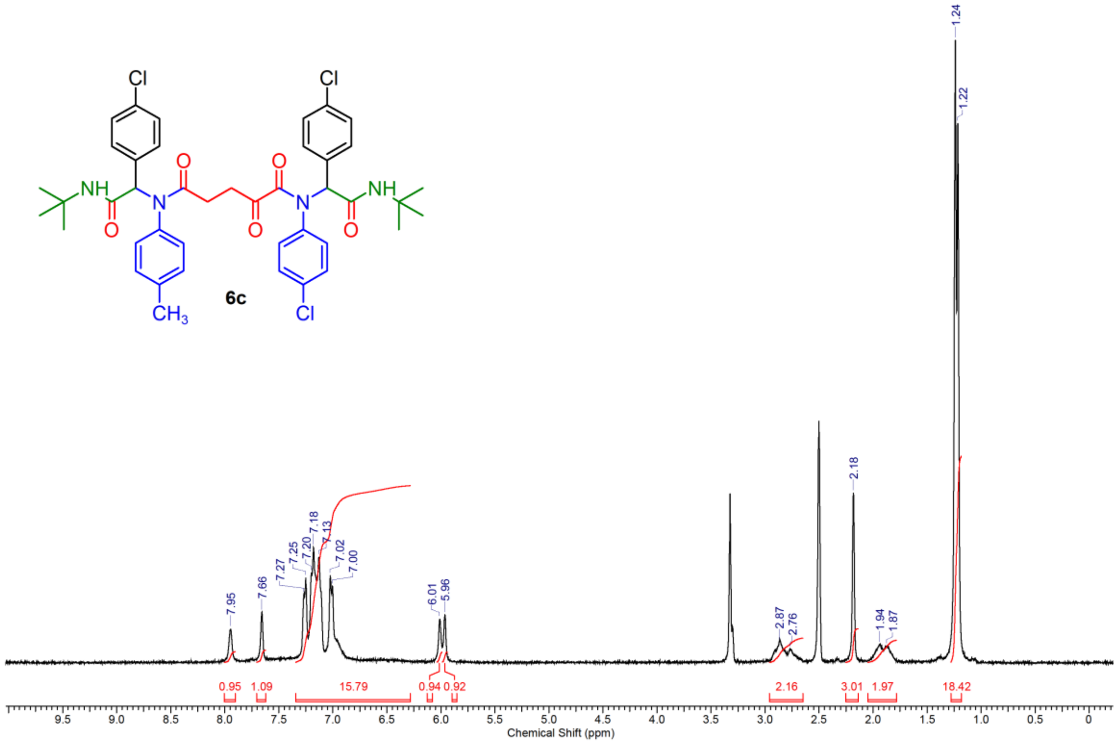

^{13}C NMR spectrum of compound **6c**

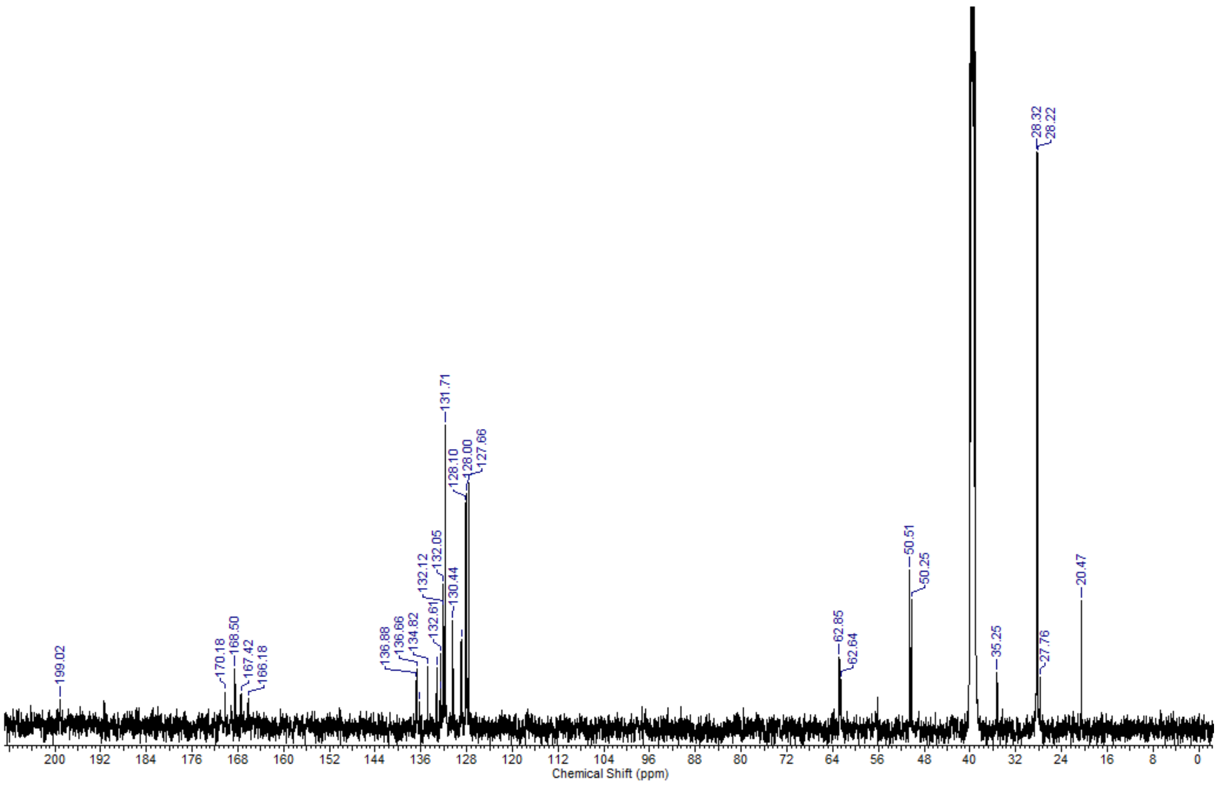

^{1}H NMR spectrum of compound **6d**

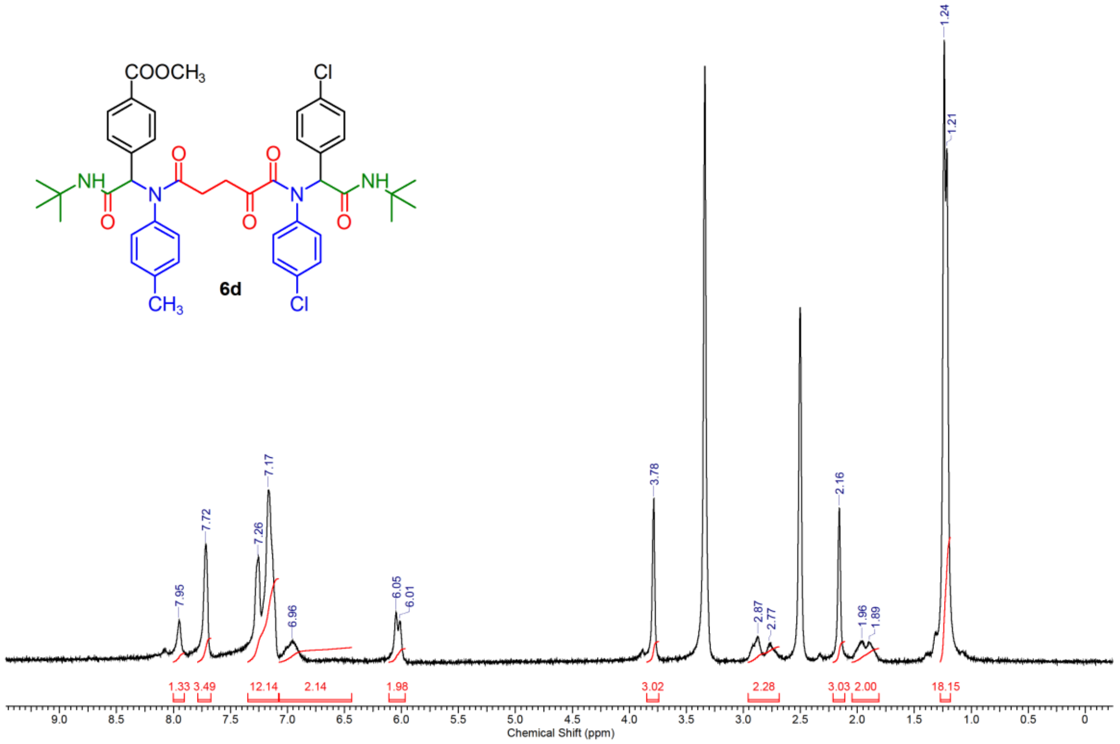

^{13}C NMR spectrum of compound **6d**

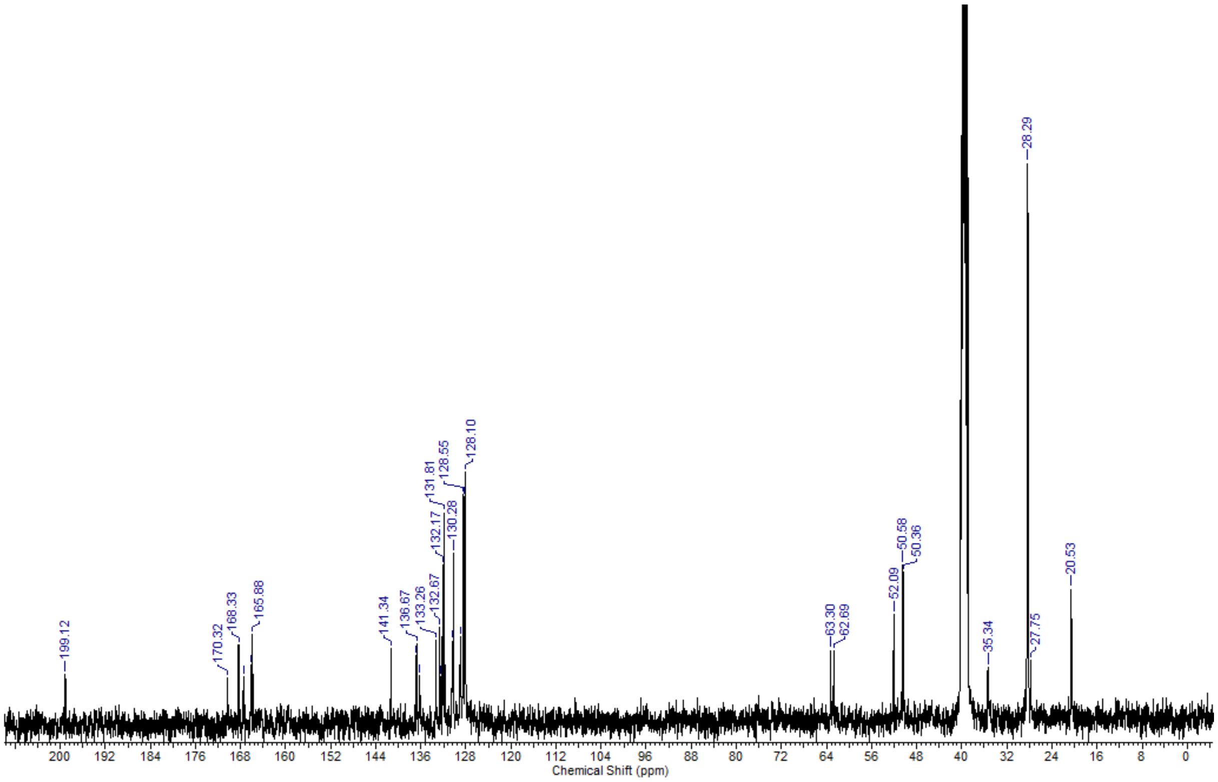

^{1}H NMR spectrum of compound **8a**

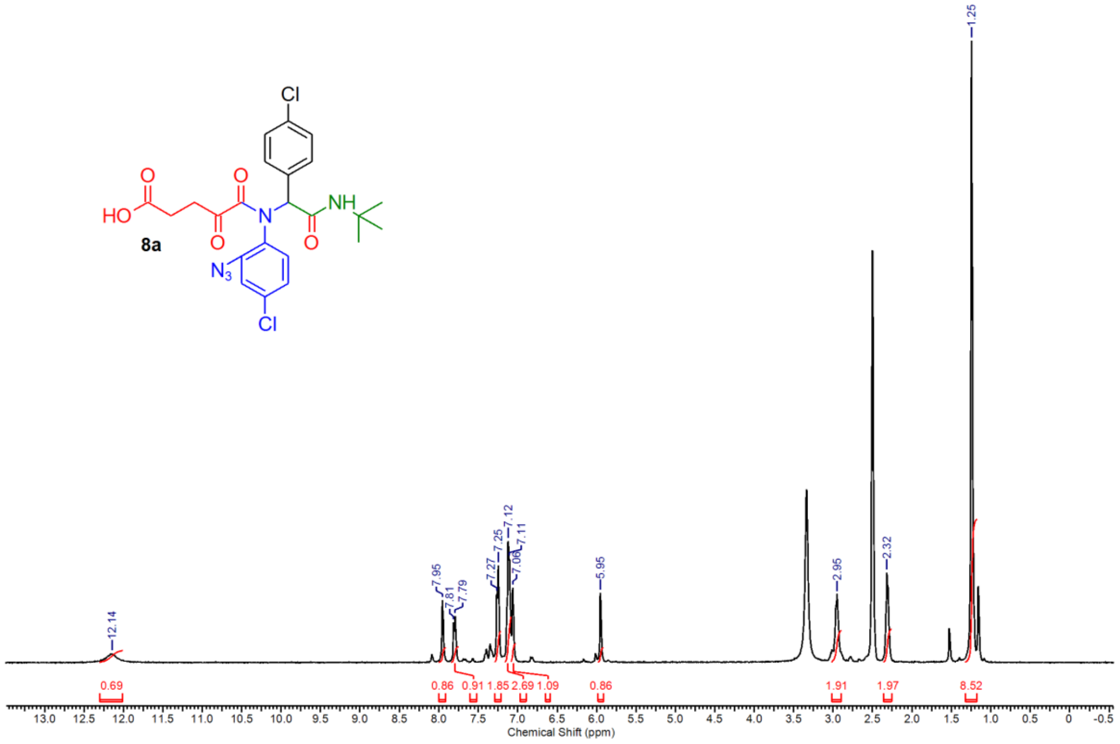

^{13}C NMR spectrum of compound **8a**

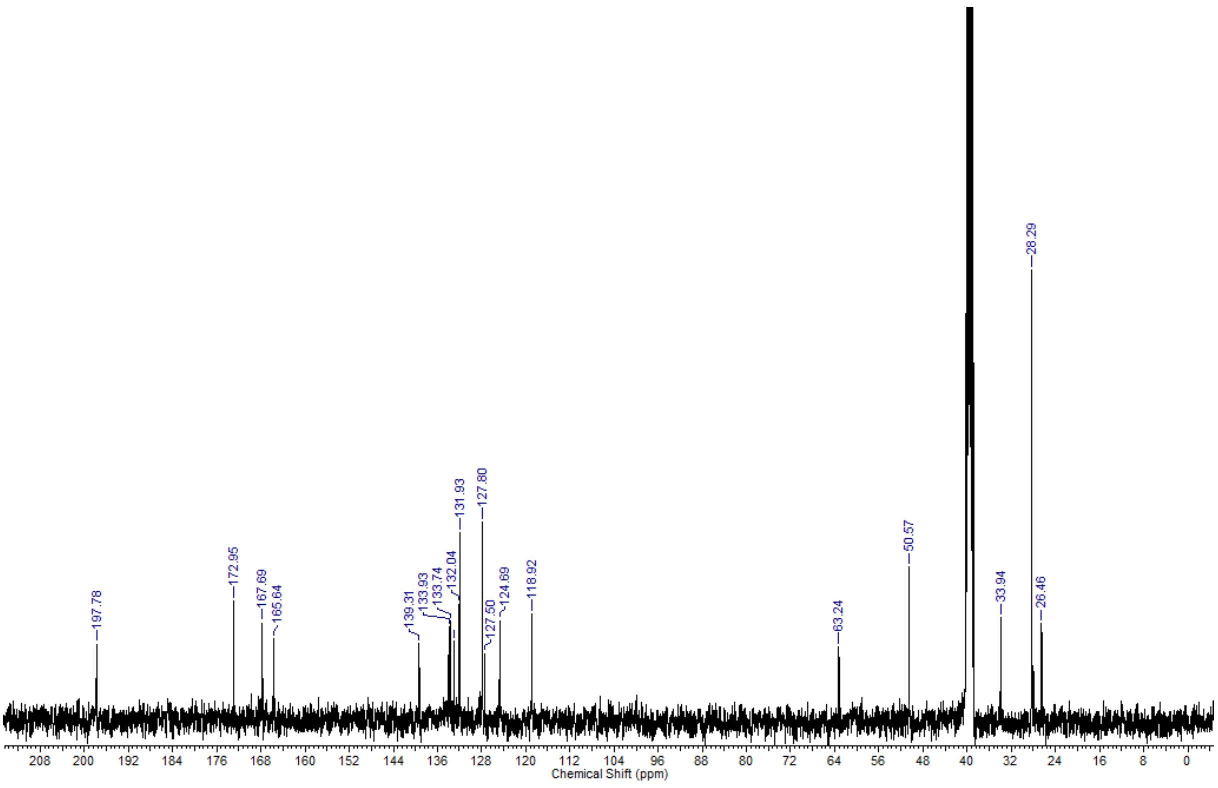

^{1}H NMR spectrum of compound **8b**

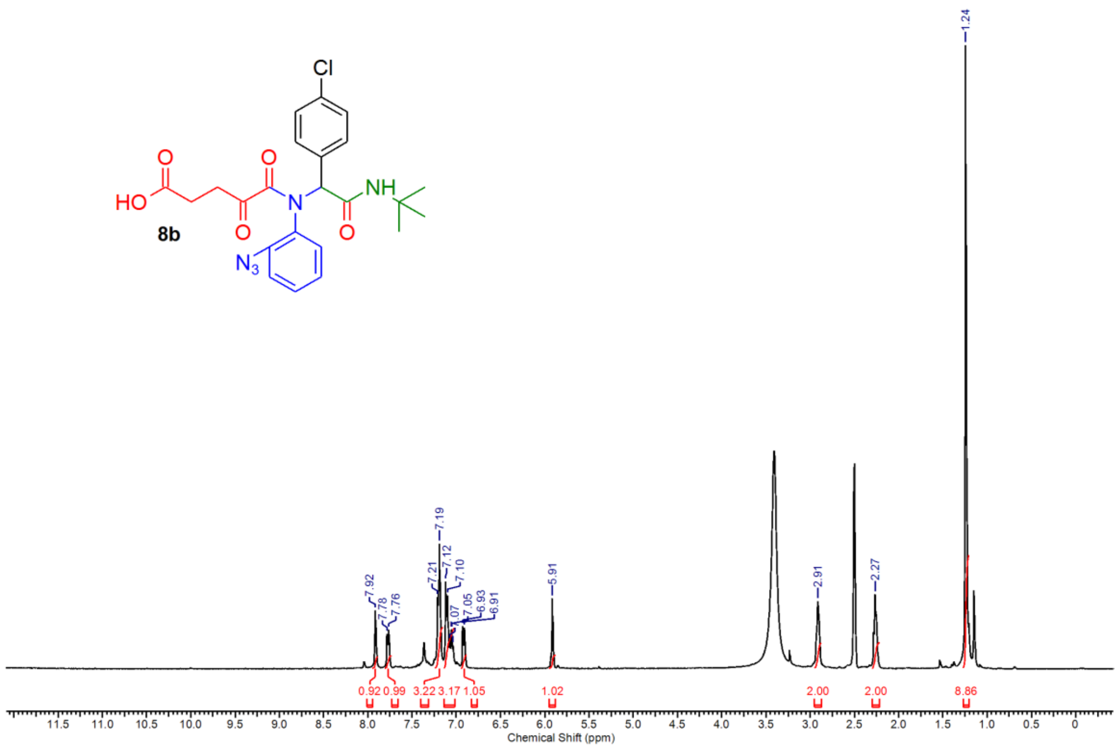

^{13}C NMR spectrum of compound **8b**

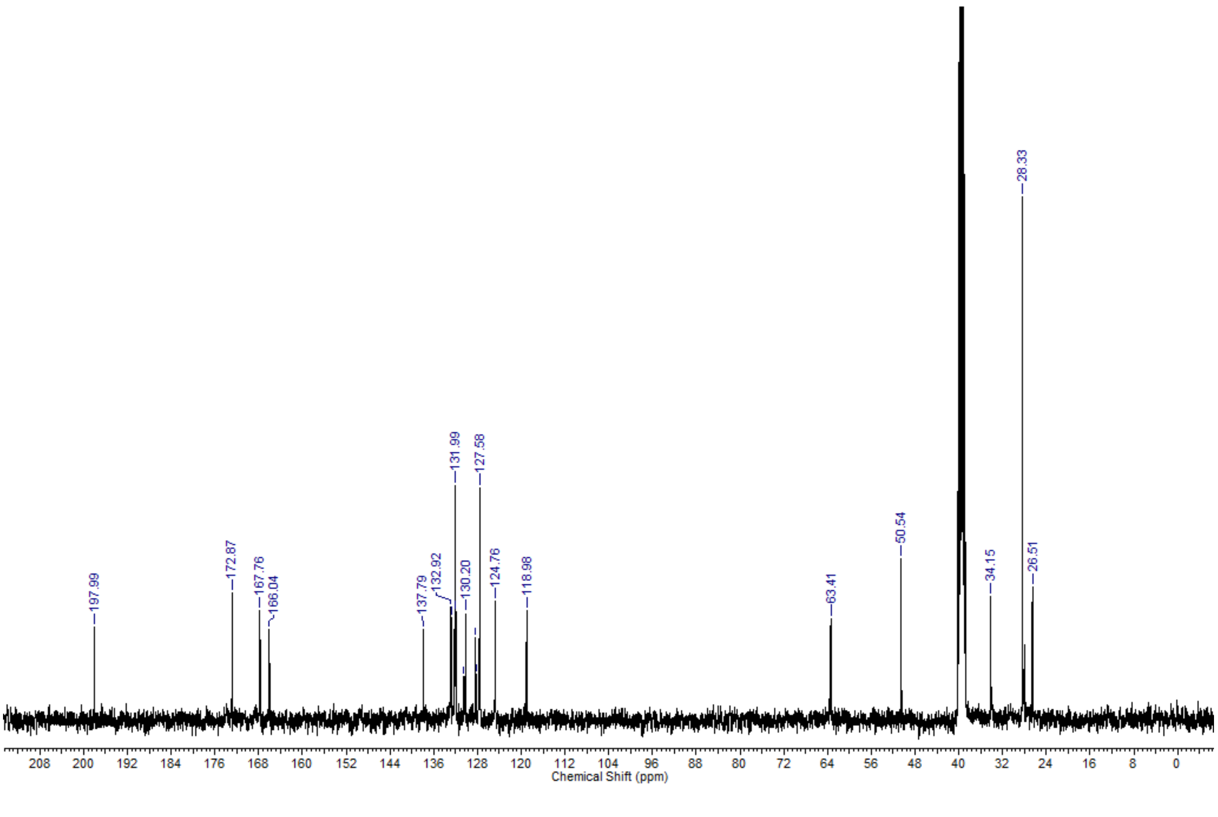

^{1}H NMR spectrum of compound **8d**

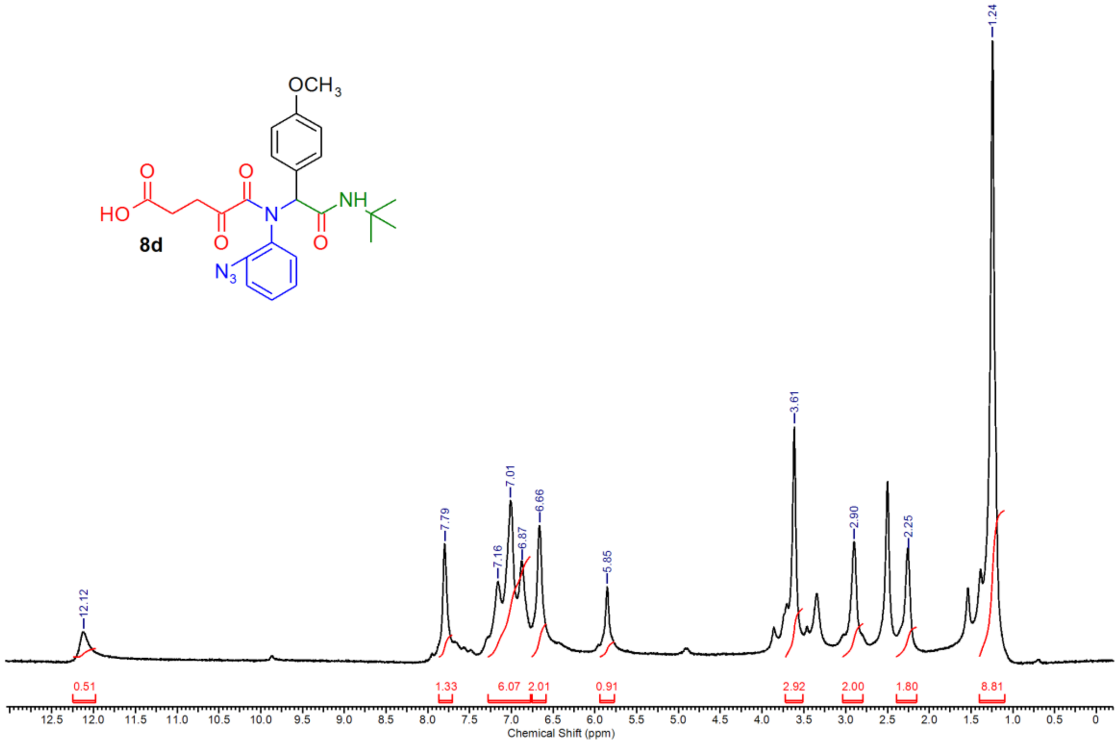

^{13}C NMR spectrum of compound **8d**

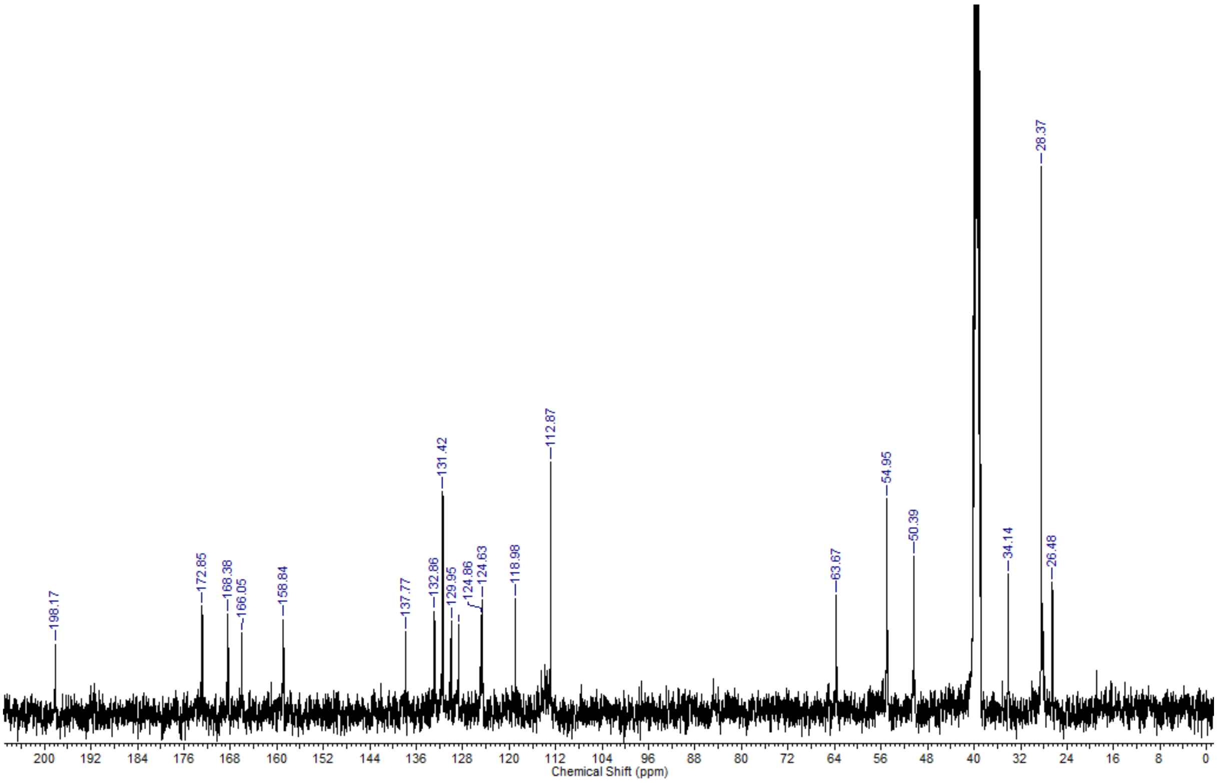

^{1}H NMR spectrum of compound **8f**

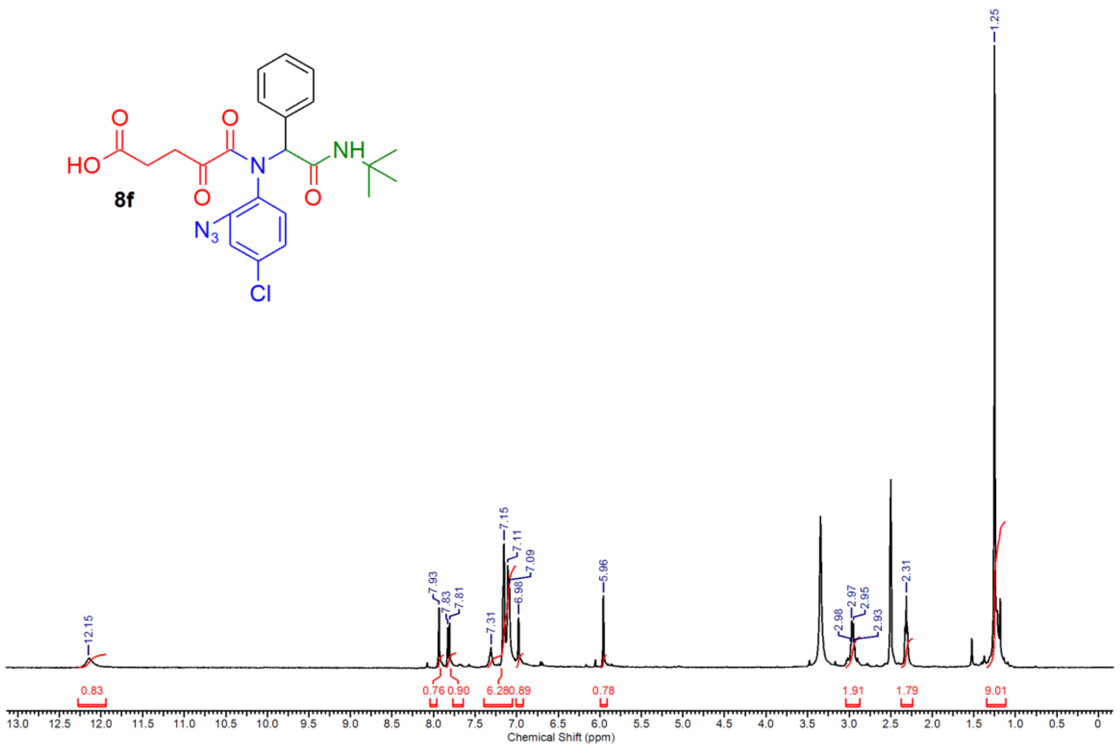

^{13}C NMR spectrum of compound **8f**

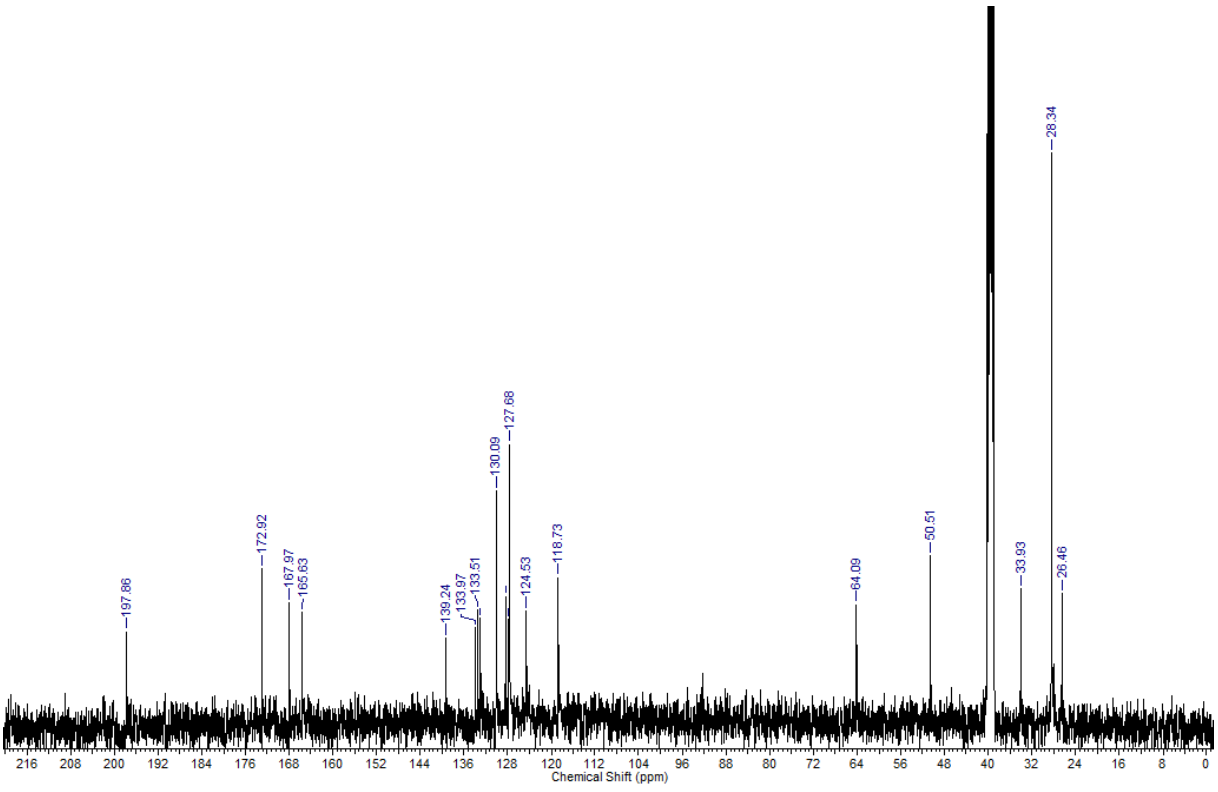

^{1}H NMR spectrum of compound **8g**

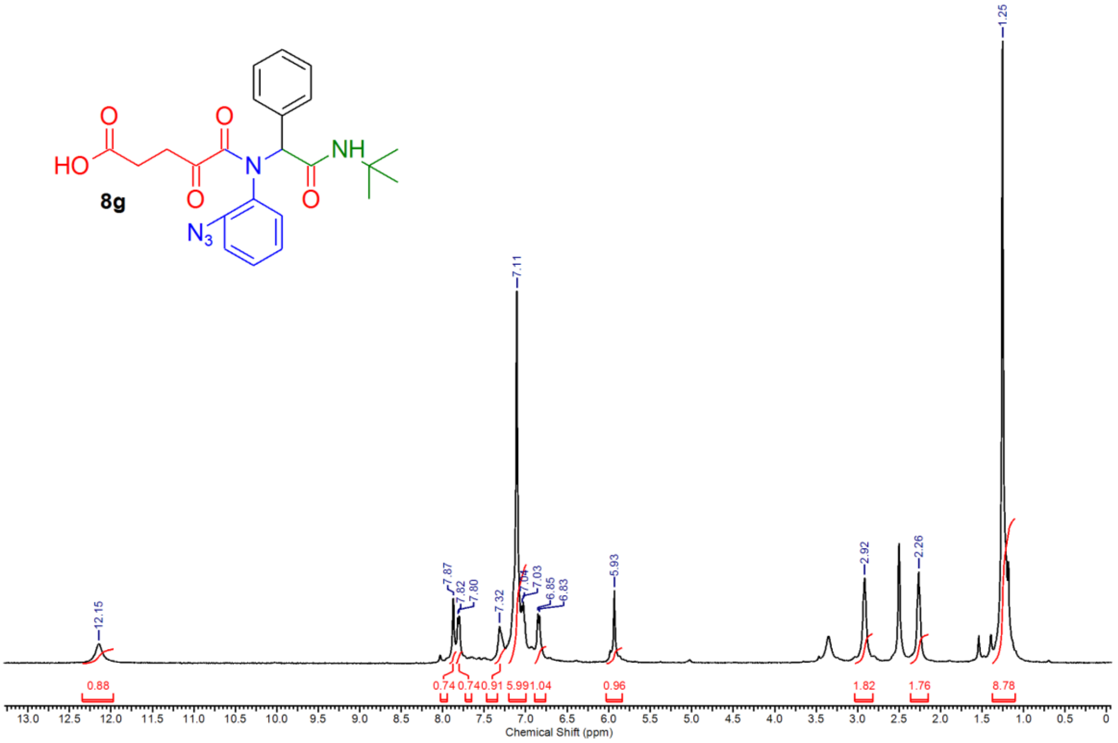

^{13}C NMR spectrum of compound **8g**

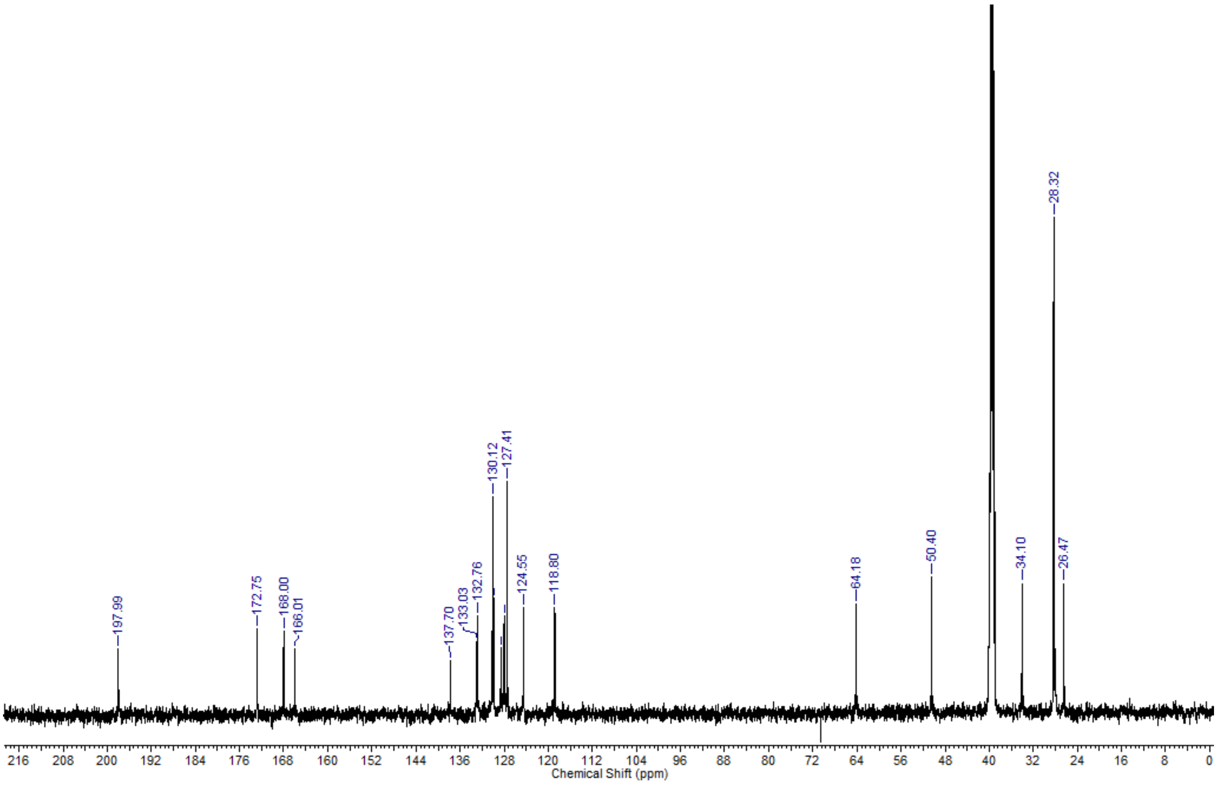

^{1}H NMR spectrum of compound **8h**

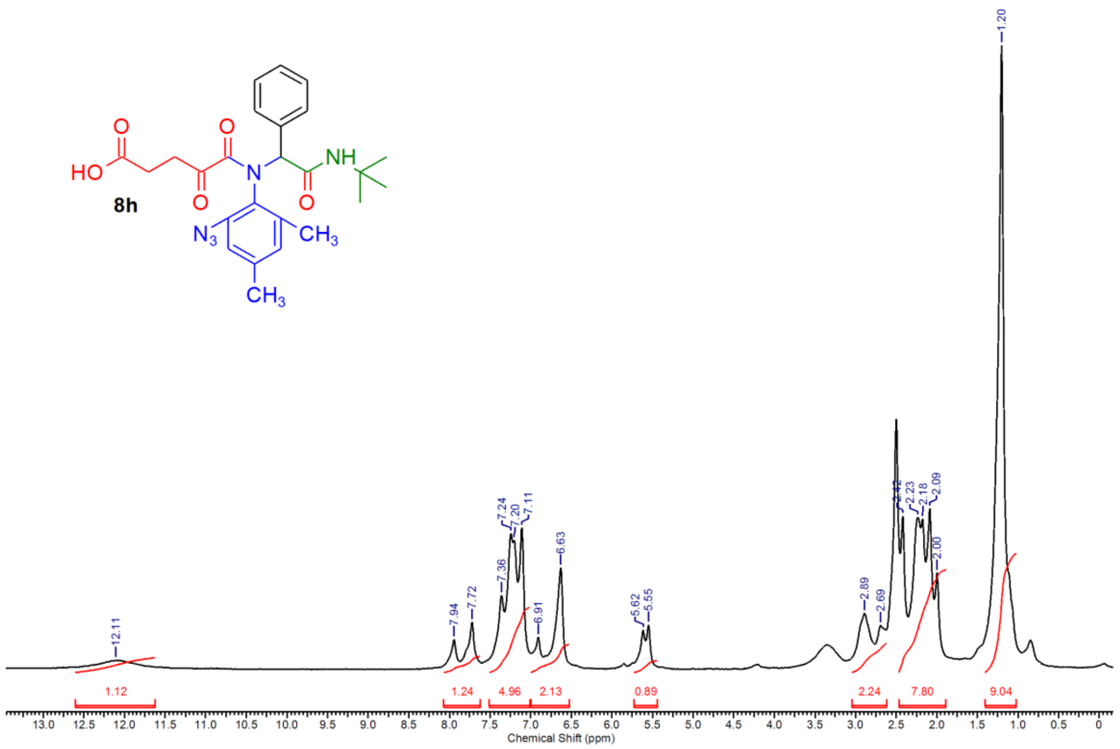

^{13}C NMR spectrum of compound **8h**

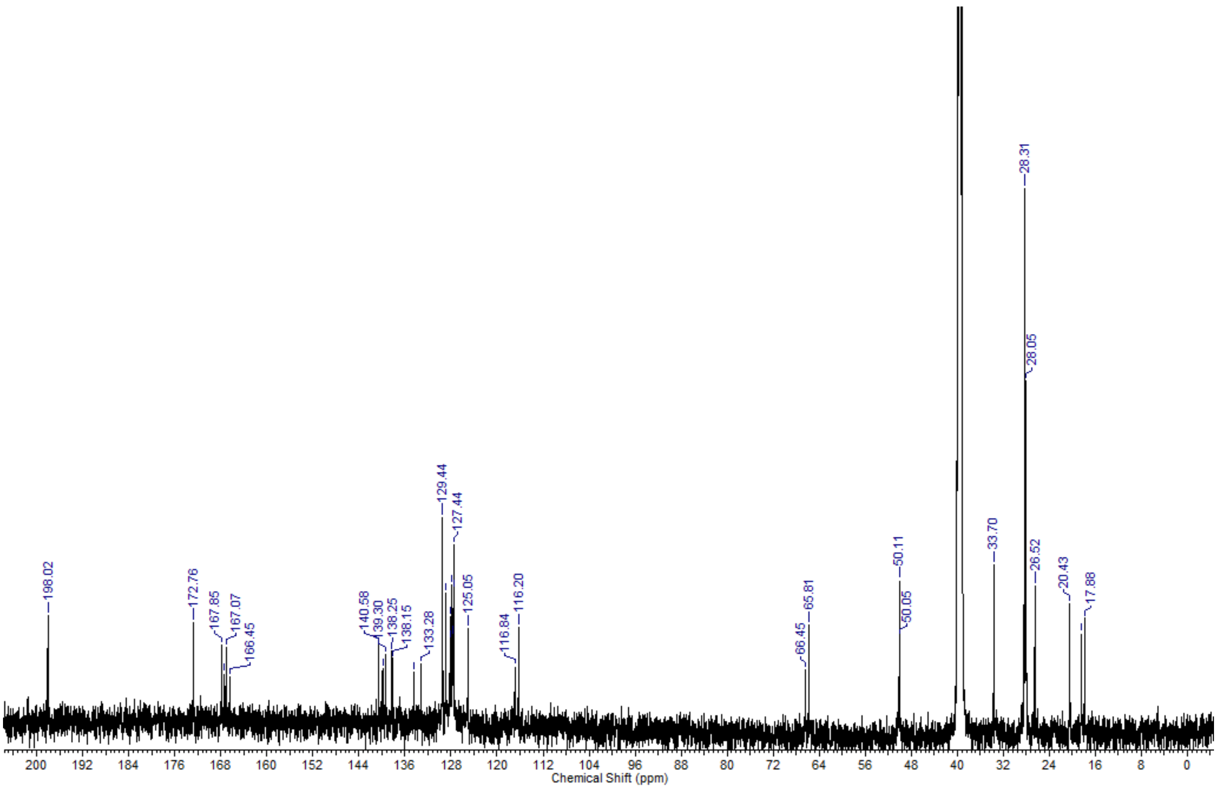

^{1}H NMR spectrum of compound **9a**

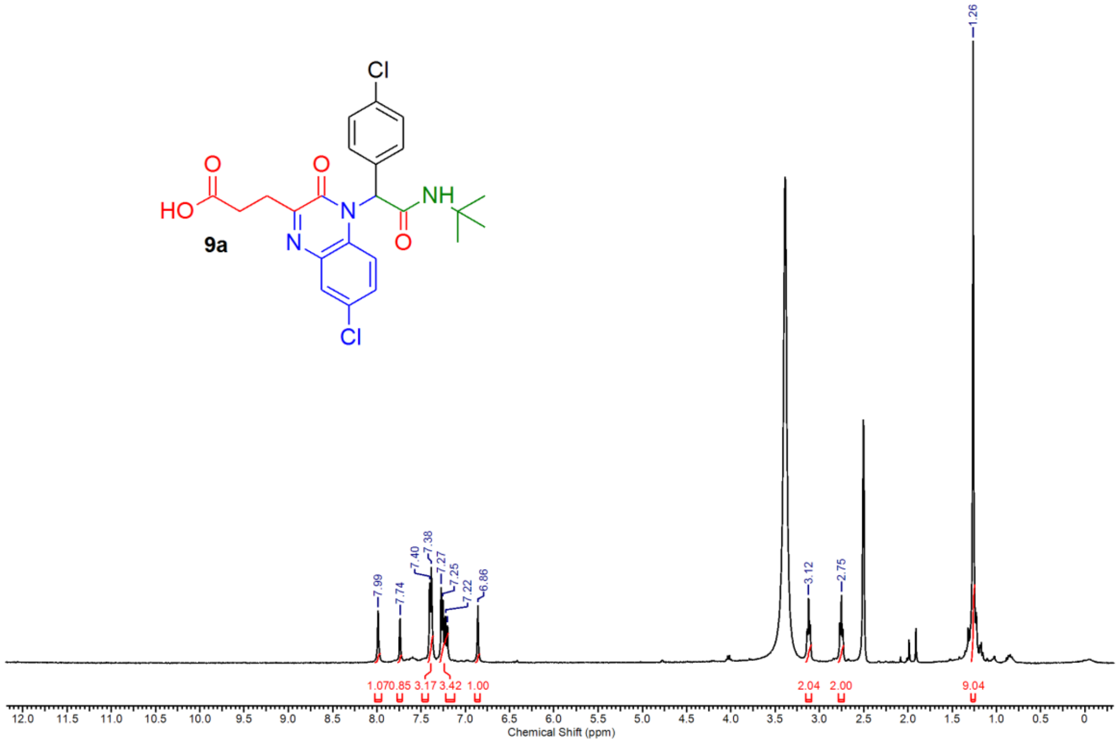

^{13}C NMR spectrum of compound **9a**

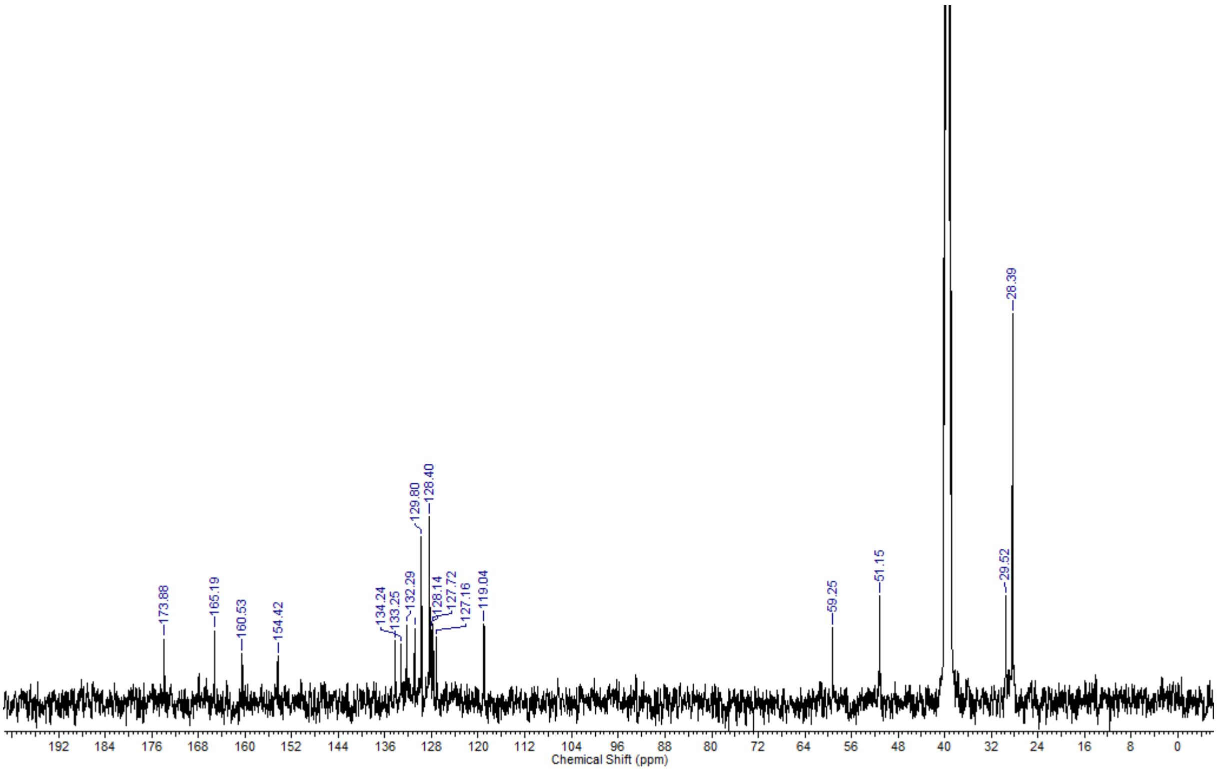

^{1}H NMR spectrum of compound **9b**

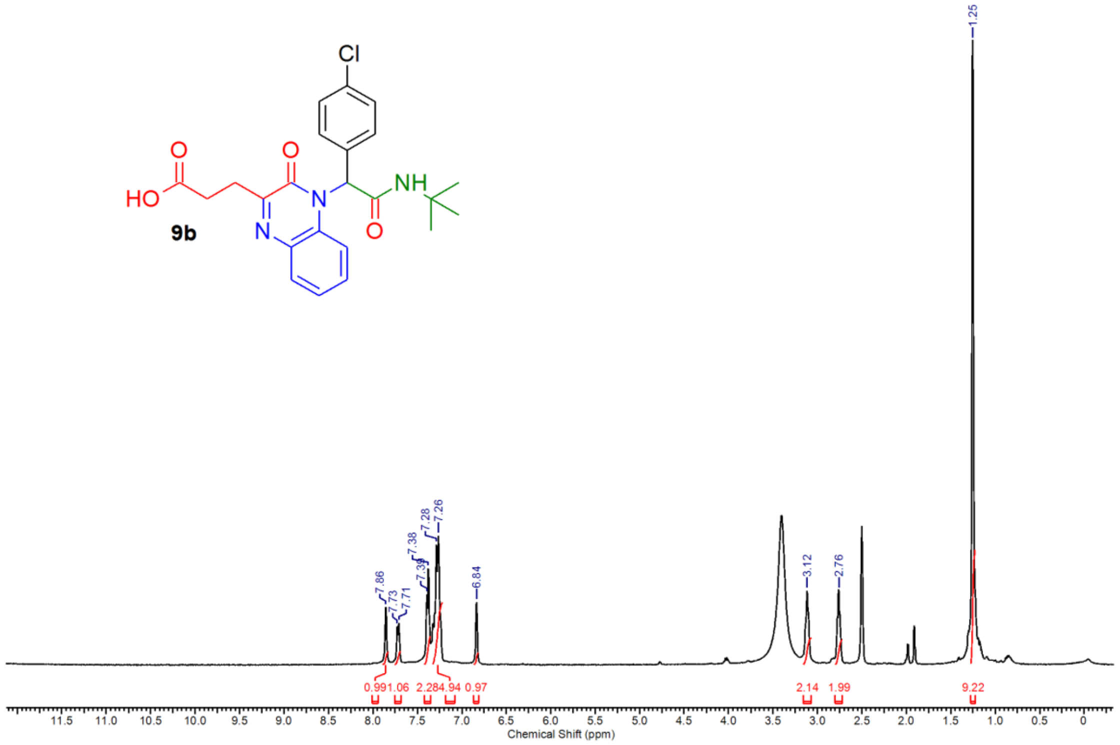

^{13}C NMR spectrum of compound **9b**

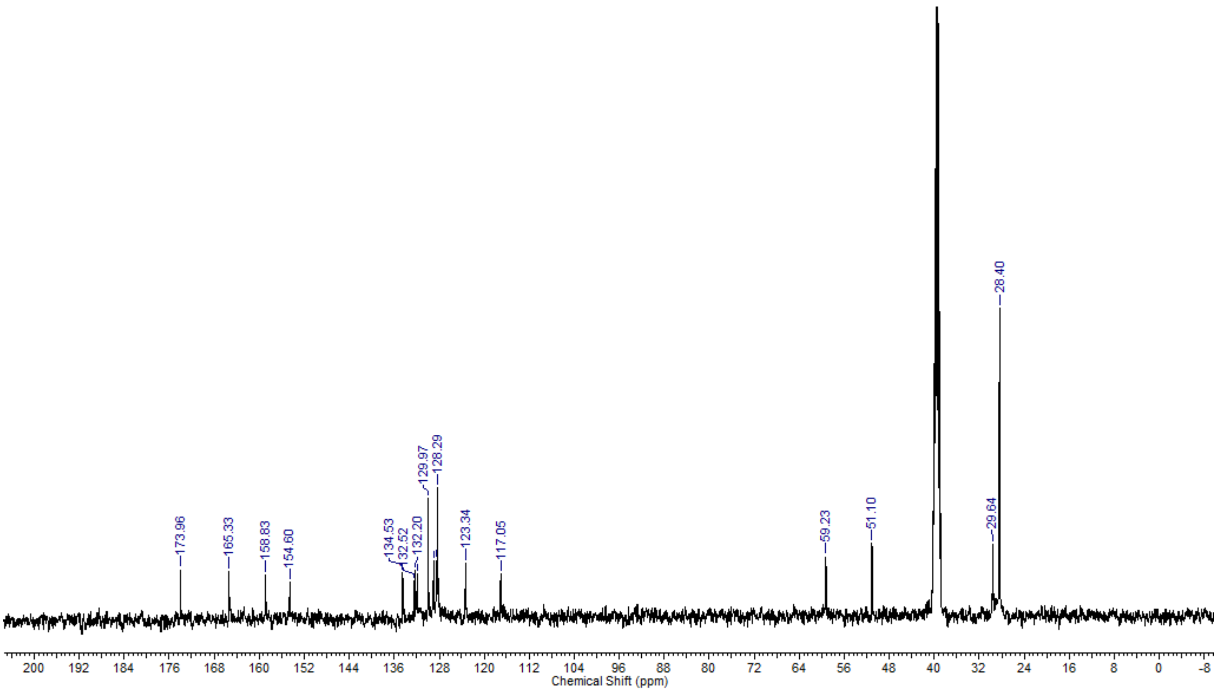

^{1}H NMR spectrum of compound **9c**

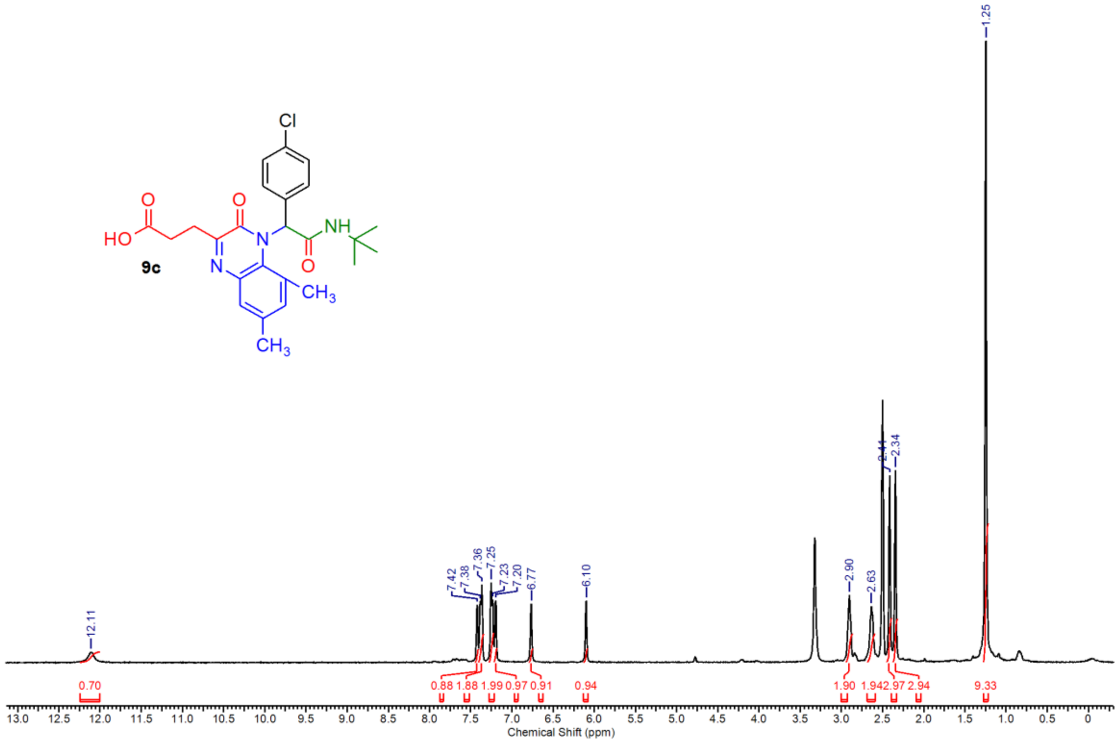

^{13}C NMR spectrum of compound **9c**

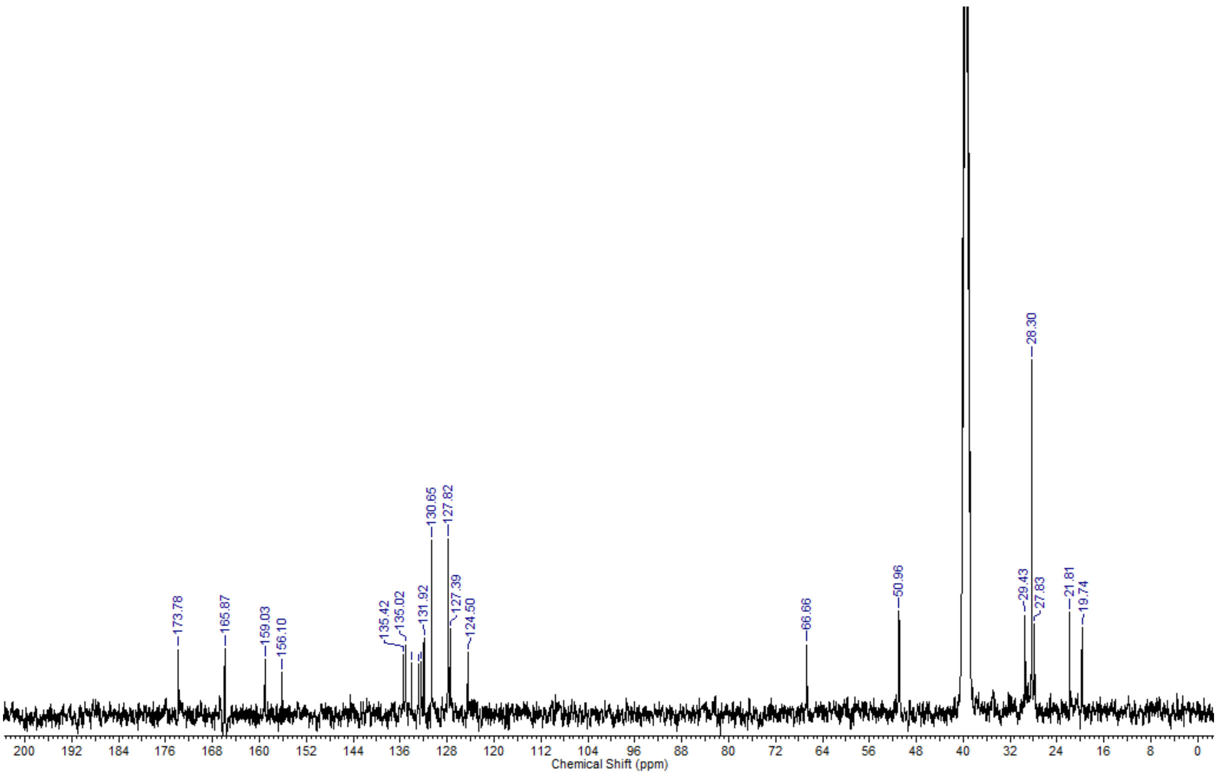

^{1}H NMR spectrum of compound **9d**

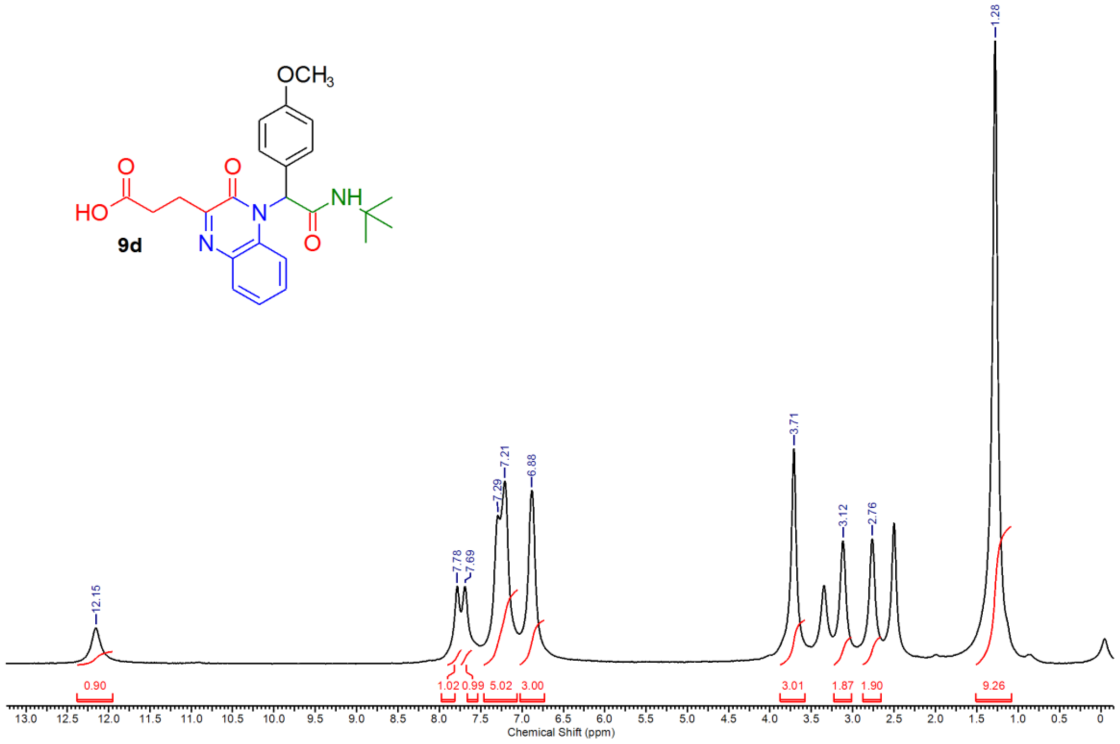

^{13}C NMR spectrum of compound **9d**

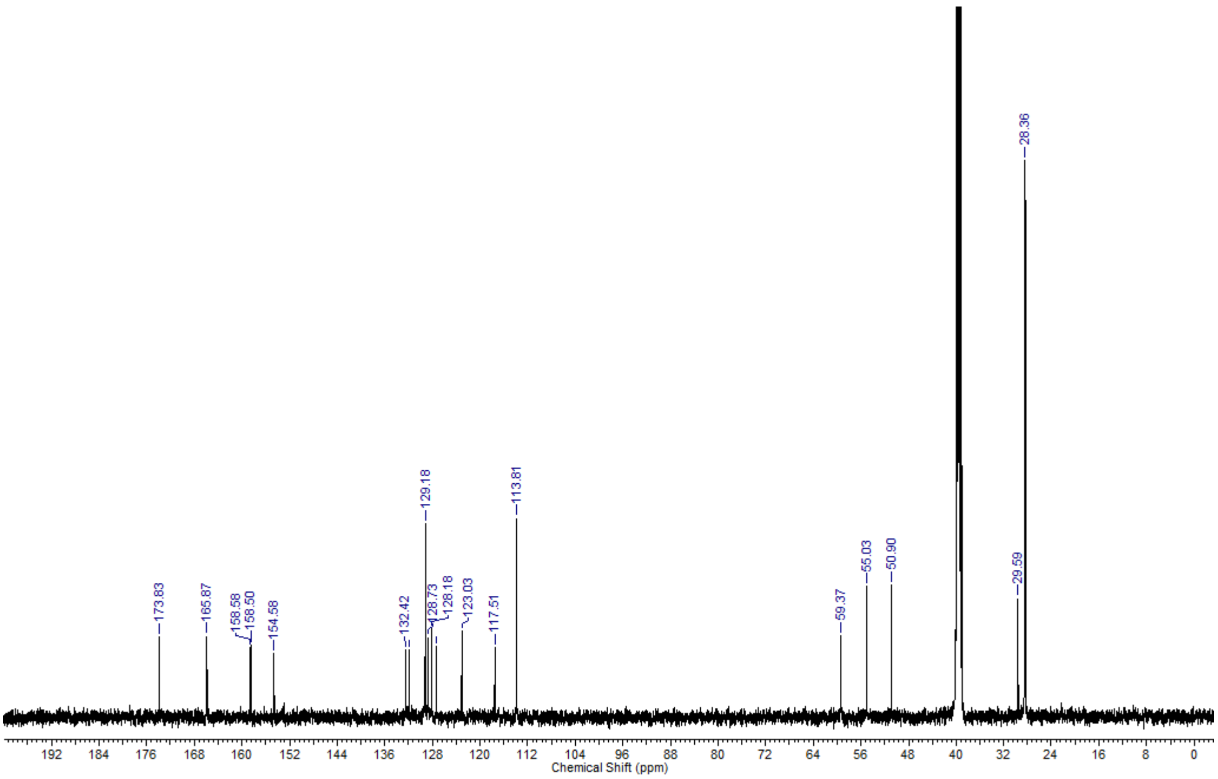

^{1}H NMR spectrum of compound **9e**

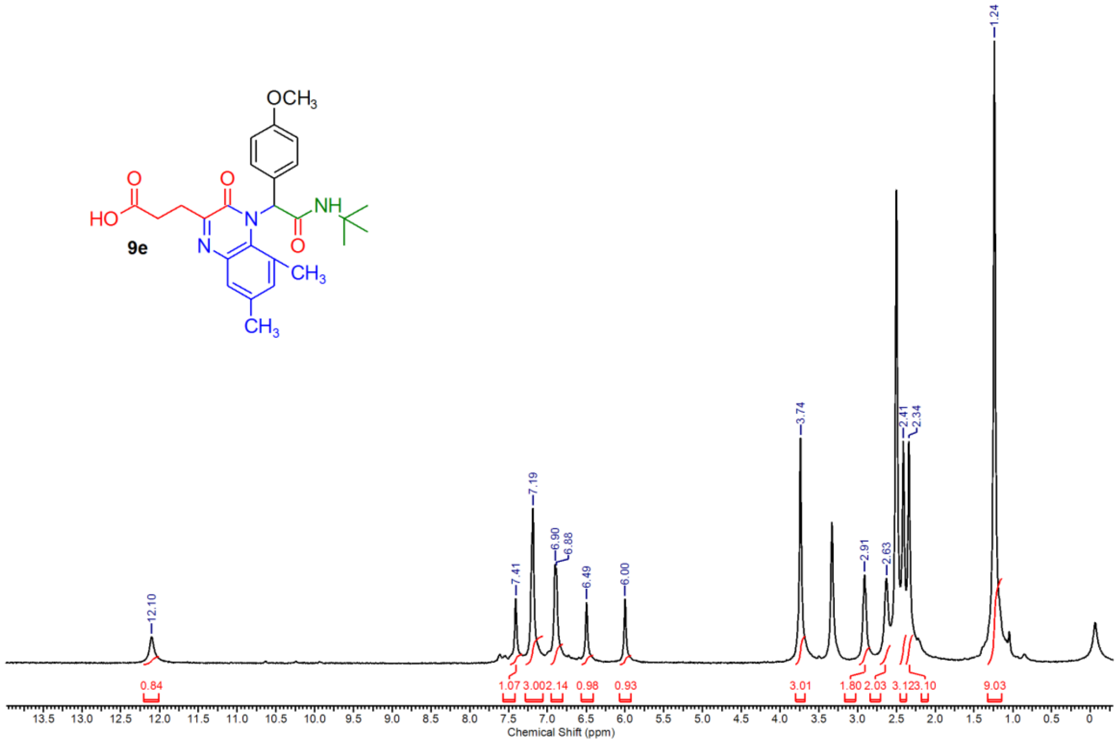

^{13}C NMR spectrum of compound **9e**

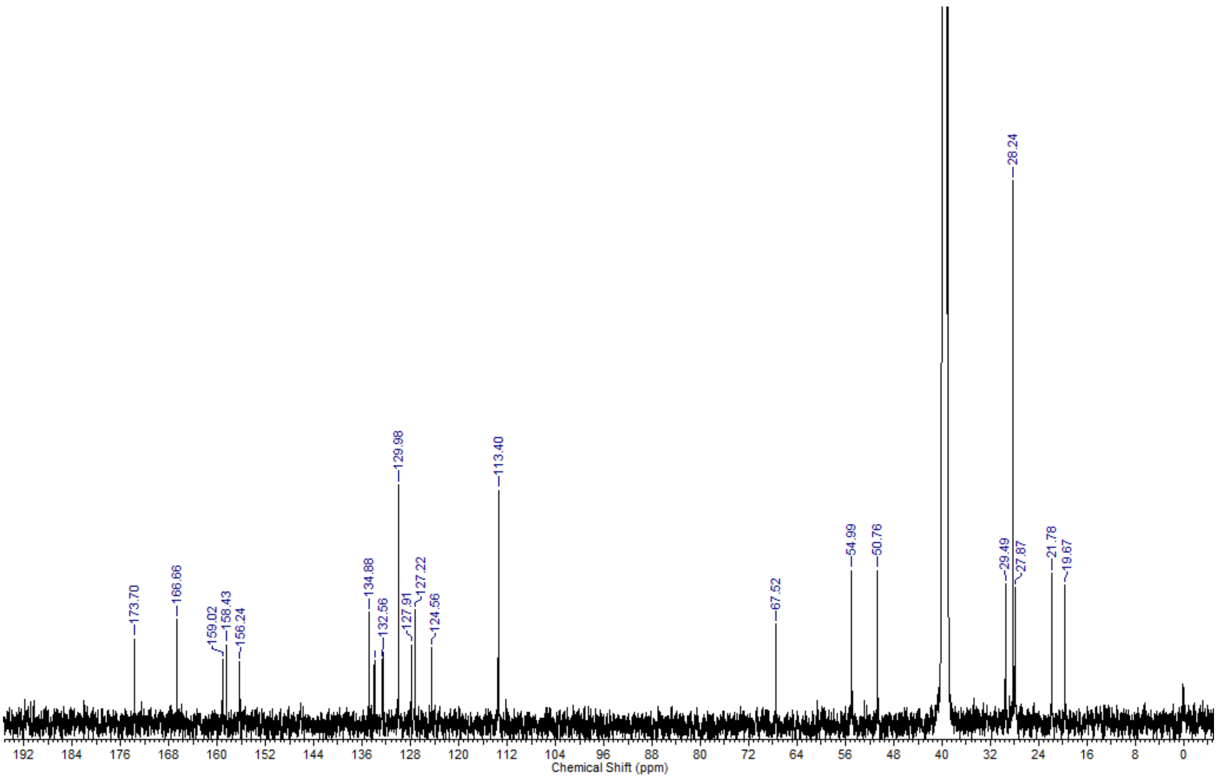

^{1}H NMR spectrum of compound **9f**

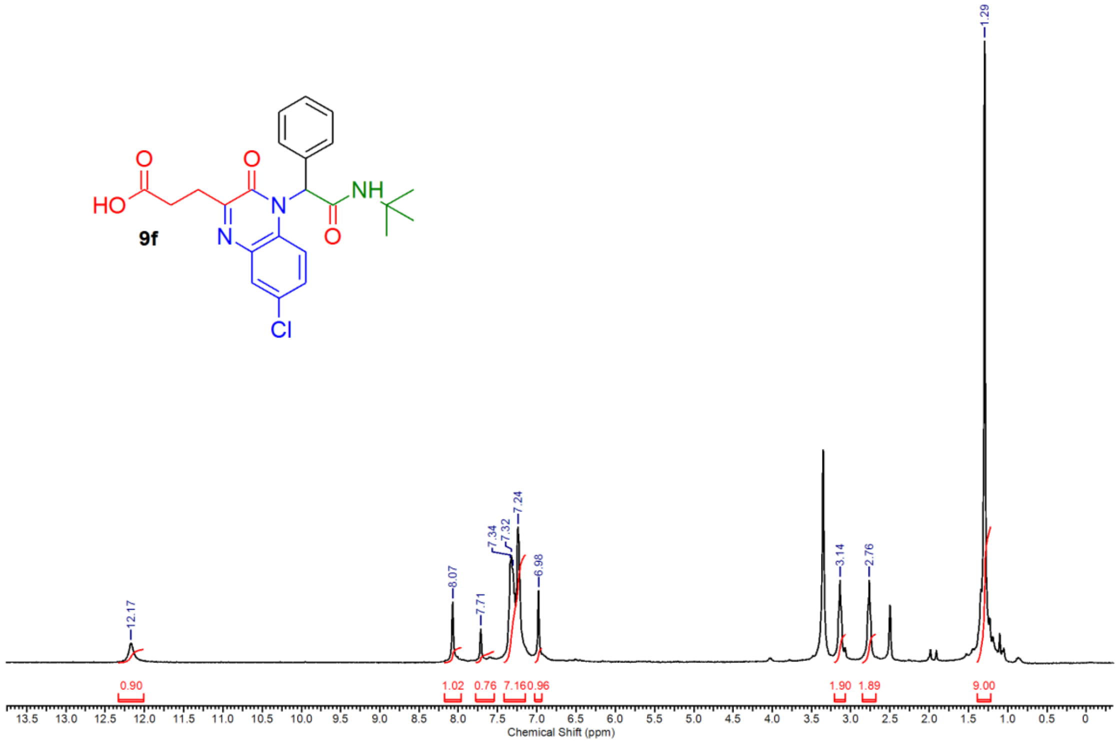

^{13}C NMR spectrum of compound **9f**

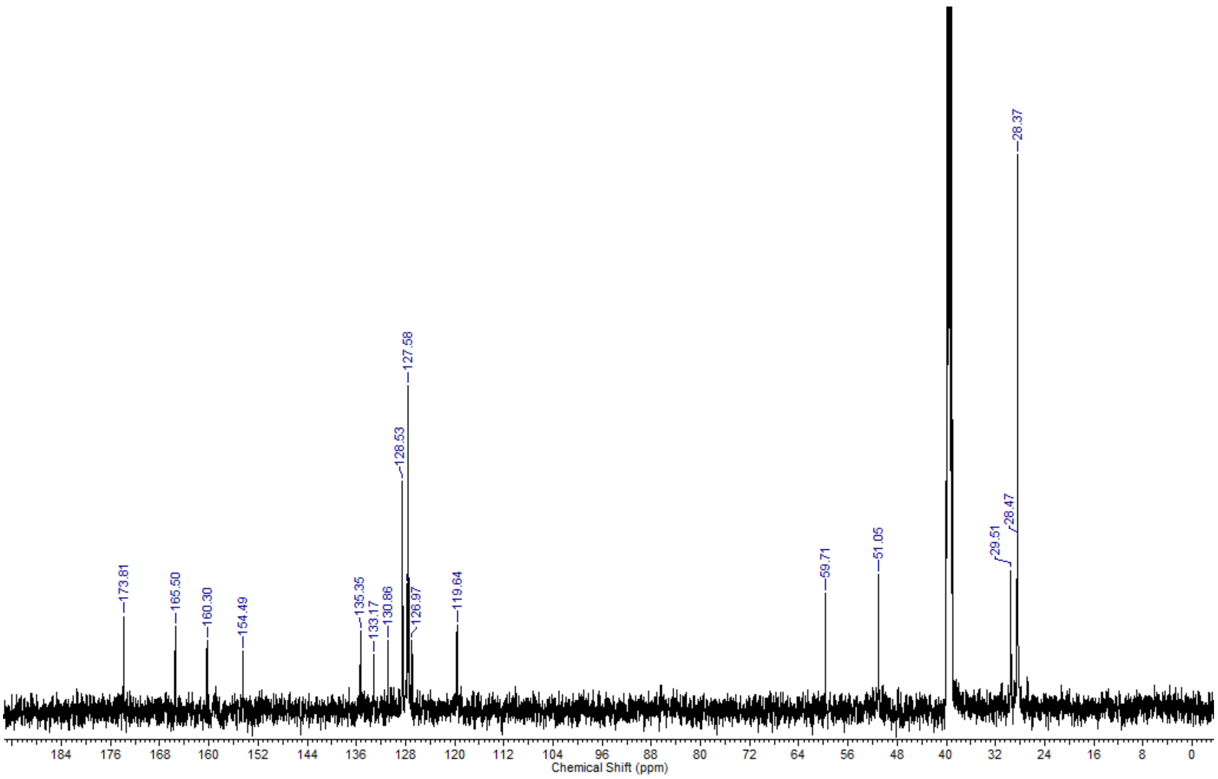

^{1}H NMR spectrum of compound **9g**

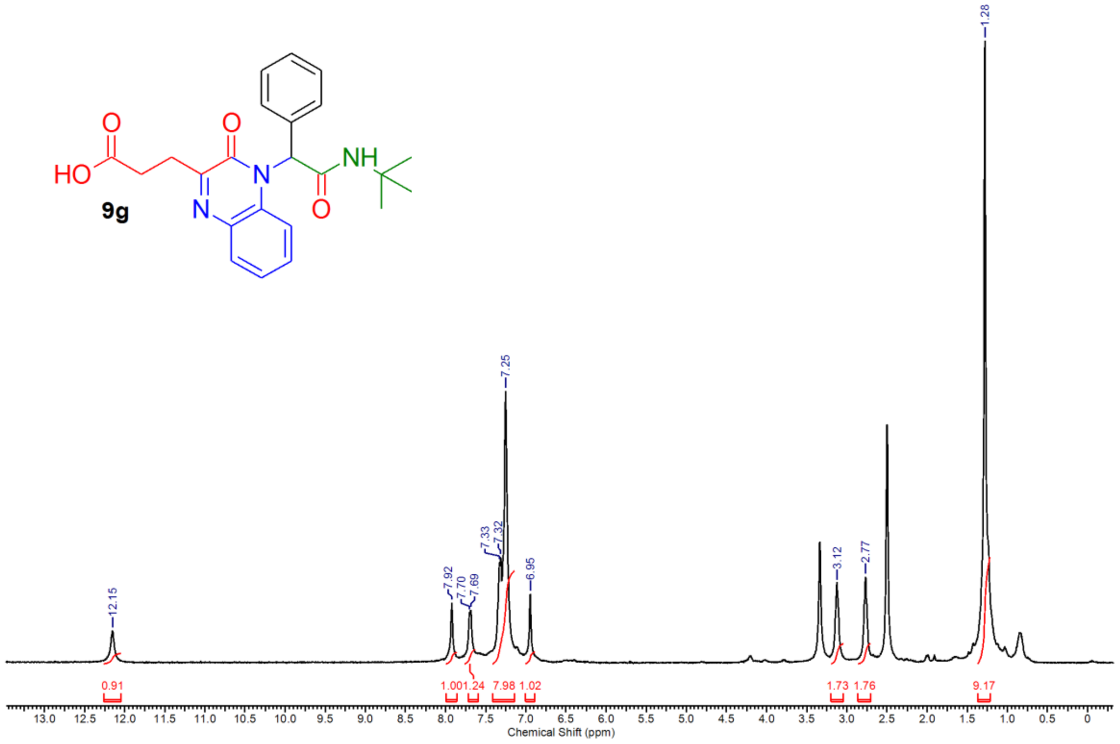

^{13}C NMR spectrum of compound **9g**

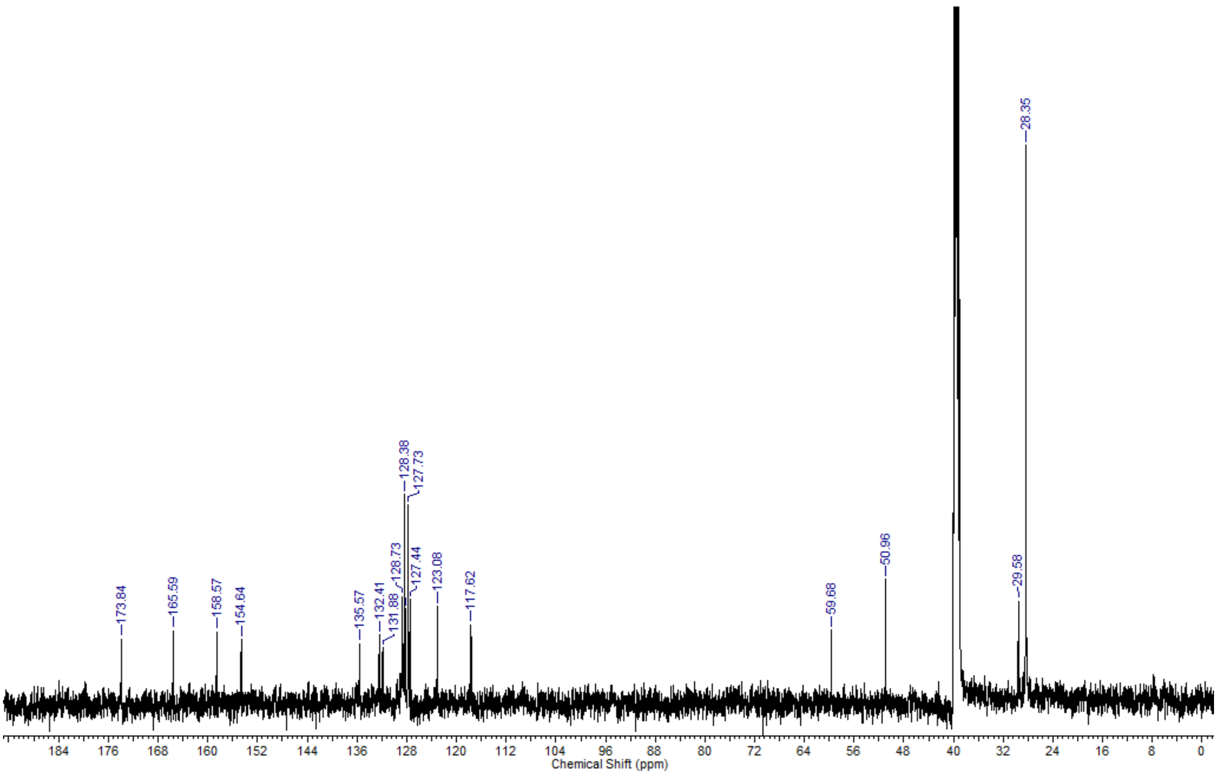

^{1}H NMR spectrum of compound **9h**

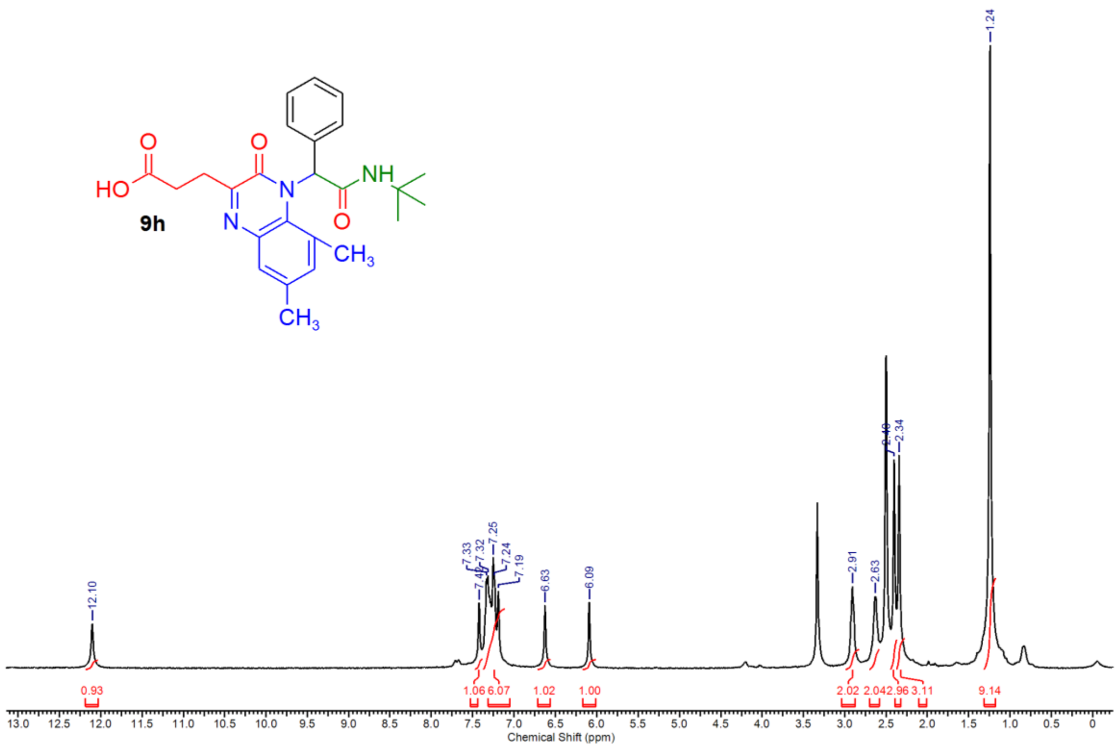

^{13}C NMR spectrum of compound **9h**

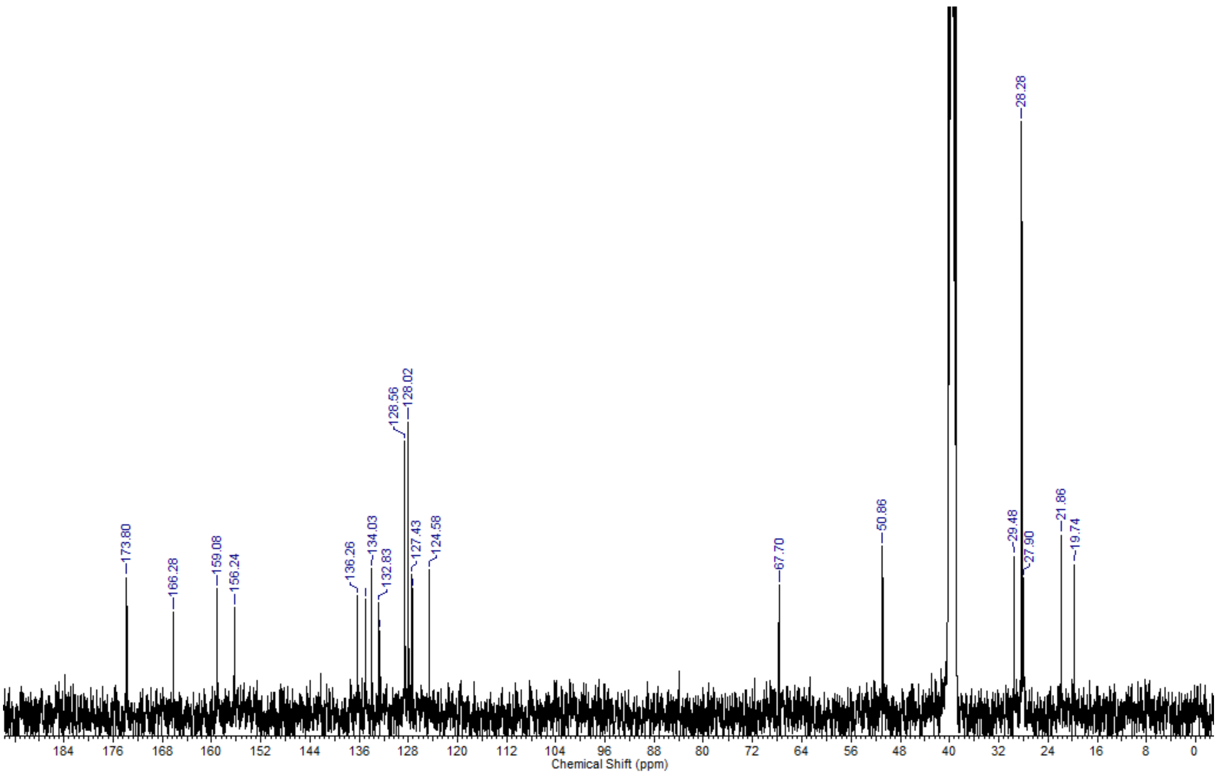

References

1. Seki, M.; Takahashi, Y. *J. Org. Chem.* **2021**, *86*, 7842–7848. doi:10.1021/acs.joc.1c00734
2. Fang, H.; Dou, Y.; Ge, J.; Chhabra, M.; Sun, H.; Zhang, P.; Zheng, Y.; Zhu, Q. *J. Org. Chem.* **2017**, *82*, 11212–11217. doi:10.1021/acs.joc.7b01594
3. Dolomanov, O. V.; Bourhis, L. J.; Gildea, R. J.; Howard, J. A. K.; Puschmann, H. *J. Appl. Crystallogr.* **2009**, *42*, 339–341. doi:10.1107/S0021889808042726
4. Sheldrick, G. M. *Acta Crystallogr. Sect. A Found. Adv.* **2015**, *71*, 3–8. doi:10.1107/S2053273314026370
5. Sheldrick, G. M. *Acta Crystallogr. Sect. C Struct. Chem.* **2015**, *71*, 3–8. doi:10.1107/S2053229614024218
